# 2,5-Diketopiperazines From a Sponge-Derived Fungus *Aspergillus sclerotiorum*

**DOI:** 10.3389/fmicb.2022.808532

**Published:** 2022-05-20

**Authors:** Chao-Yi Wang, Xiao-Han Liu, Yao-Yao Zheng, Xing-Yan Ning, Ya-Hui Zhang, Xiu-Mei Fu, Xin Li, Chang-Lun Shao, Chang-Yun Wang

**Affiliations:** ^1^Key Laboratory of Marine Drugs, The Ministry of Education of China, School of Medicine and Pharmacy, Ocean University of China, Qingdao, China; ^2^Laboratory for Marine Drugs and Bioproducts, Qingdao National Laboratory for Marine Science and Technology, Qingdao, China; ^3^Institute of Evolution and Marine Biodiversity, Ocean University of China, Qingdao, China

**Keywords:** *Aspergillus sclerotiorum*, 2, 5-diketopiperazines, sponge-derived fungus, bicyclo[2.2.2]diazaoctane, DNA topoisomerase I

## Abstract

Three new 2,5-diketopiperazines, speramide C (**1**), 3,21-*epi*-taichunamide F (**2**), and 2-*epi*-amoenamide C (**3**), along with four known analogs (**4**–**7**), were obtained from the sponge-derived fungus *Aspergillus sclerotiorum* GDST-2013-0501 collected from the South China Sea. The chemical structures of new compounds were elucidated by analyzing NMR and MS spectroscopy data, and their absolute configurations were determined by electronic circular dichroism (ECD) calculations. Compound **1** represents the first prenylated indole alkaloid with an ethylene oxide ring at the isopentenyl side chain. Compound **4** displayed DNA topoisomerase I inhibitory activity and antibacterial activity against *Staphylococcus epidermidis*. The low cytotoxic or non-cytotoxic compound **4** displayed DNA topoisomerase I inhibitory activity, which could provide a starting point for the development of antitumor agents.

## Introduction

**Figure 1:**
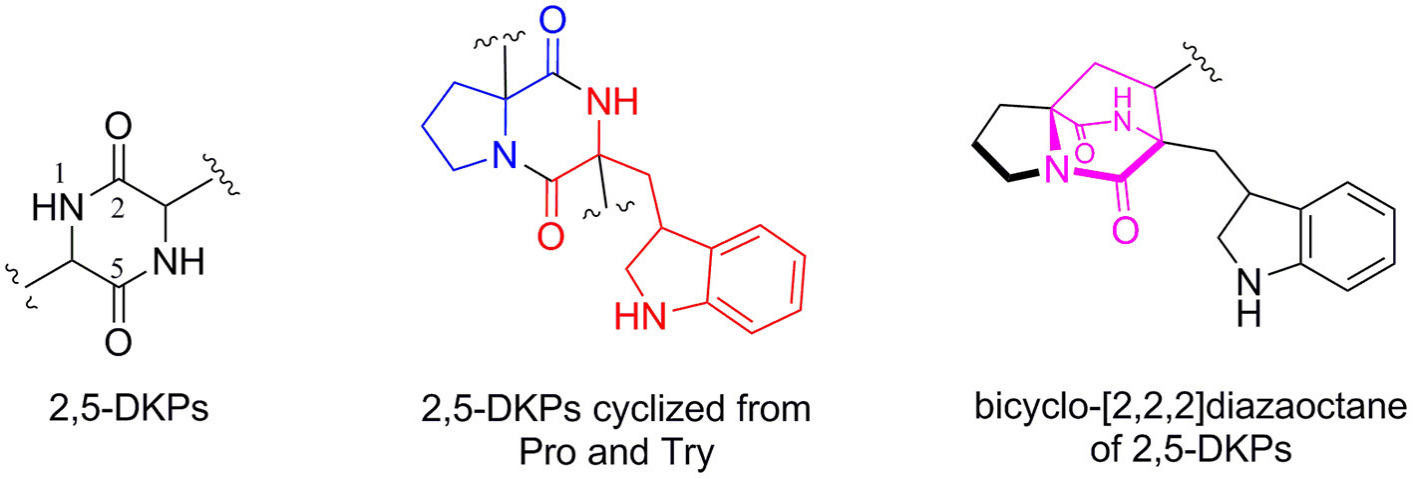
The core structure of 2,5-DKPs and the substructure of common 2,5-DKPs cyclized from Try and Pro.

2,5-diketopiperazines (2,5-DKPs) are important cyclodipeptides (piperazine-2,5-dione core) characterized by the dipeptide core derived from the “head to tail” cyclization of two amino acids (Li et al., 2009; Huang et al., 2014; Ma et al., 2016; Chekan and Moore, 2018). Structurally, these natural compounds usually arise from the oxidative condensation of several isoprene units, tryptophan, and other cyclic amino acid residues such as proline, phenylalanine, tryptophan, histidine, or leucine. Moreover, the prenylated 2,5-DKPs cyclized from tryptophan and proline (Figure 1) possessed a range of interesting structural and stereochemical features that were previously found in a variety of natural products from fungi, bacteria, marine invertebrates, plants, and mammals (Greshock et al., 2008; Li et al., 2009; Huang et al., 2014; Ma et al., 2016). It was reported that marine-derived fungi have been shown to be the rich sources of 2,5-DKP derivatives, in particular *Aspergillus* and *Penicillium* spp (Huang et al., 2014; Ma et al., 2016). Significantly, a myriad of biological activities, including antimicrobial, antitumor, antiviral, insecticidal activities, and anthroprotective effects, were displayed by members of this family (Borthwick, 2012; Ma et al., 2016; Borthwick and Costa, 2017; Chen et al., 2018).

**Figure 2:**
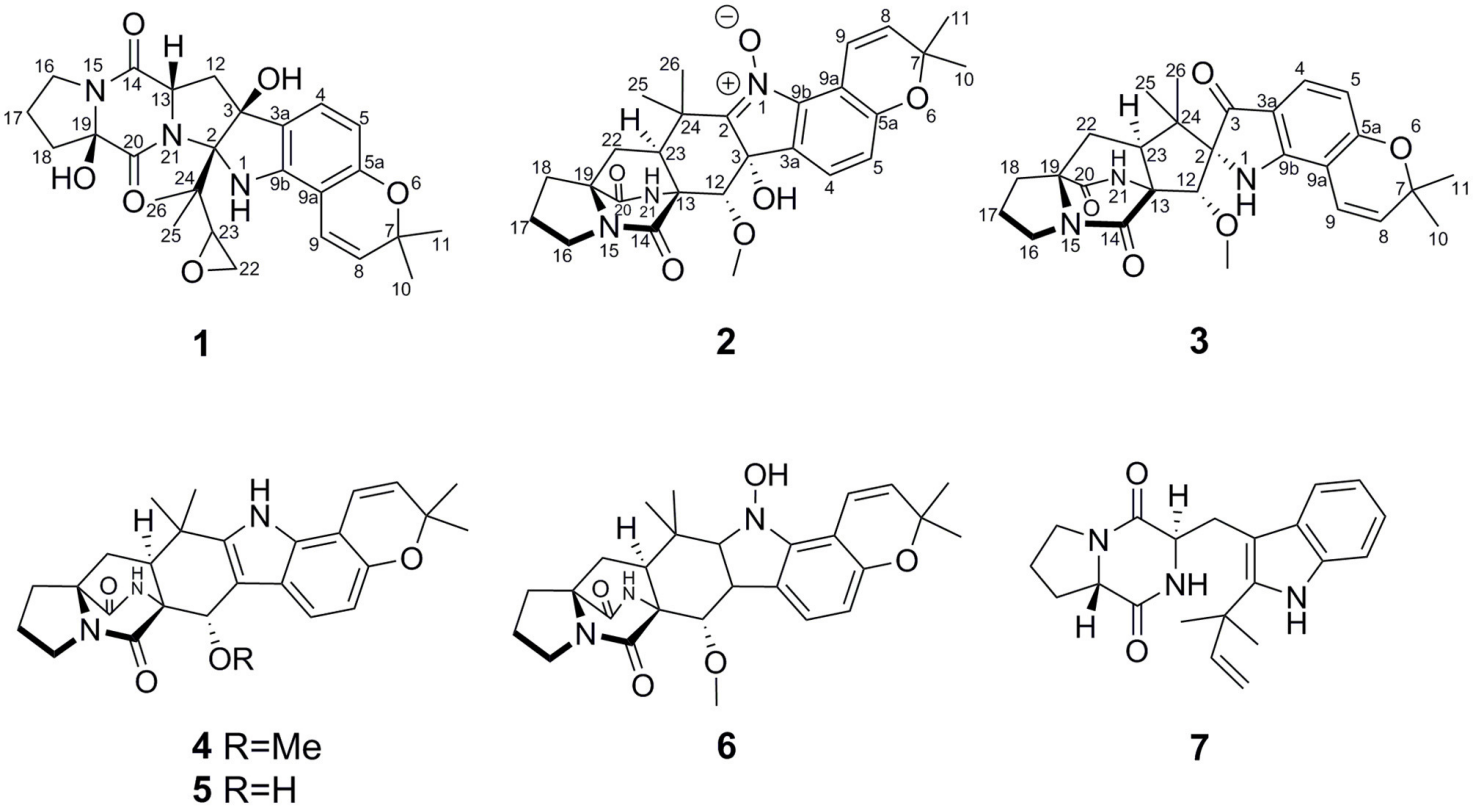
The structures of the isolated compounds.

During our ongoing search for new bioactive secondary metabolites from the marine-derived fungi *Aspergillus* in the South China Sea (Chen et al., 2014a,b; Liu et al., 2019; Wu et al., 2020; Chao et al., 2021), a sponge-derived fungal strain, *A. sclerotiorum* GDST-2013-0501, attracted our attention because the extract of the fungal culture showed antibacterial activity. Chemical investigation of the ethyl acetate extract led to the isolation of three new 2,5-DKP derivatives and four known analogs (Figure 2). Herein, we report the isolation, structure elucidation, and biological activities of these compounds.

## Materials and Methods

### General Experimental Procedure

Optical rotations were measured on a JASCO P-1020 digital polarimeter. UV spectra were recorded on a Beckman DU 640 spectrophotometer. ECD spectra were recorded on a Jasco J-815-150S circular dichroism spectrometer. IR spectra were recorded on a Nicolet-Nexus-470 spectrometer using KBr pellets. ^1^H, ^13^C, and 2D NMR spectra were recorded on an Agilent DD2 500 MHz NMR spectrometer (500 MHz for ^1^H and 125 MHz for ^13^C), using TMS as an internal standard. The HRESIMS and ESIMS spectra were obtained from a Micromass Q-TOF spectrometer. A semi-preparative HPLC was performed on a Hitachi L-2000 HPLC system coupled with a Hitachi L-2455 photodiode array detector. A Kromasil C_18_ semi-preparative HPLC column (250 mm × 10 mm, 5 μm) was used. Silica gel (Qing Dao Hai Yang Chemical Group Co.; 200–300 mesh), Sephadex LH-20 (Amersham Biosciences), and octadecylsilyl silica gel (Unicorn; 45–60 μm) were used for column chromatography (CC). Precoated silica gel GF_254_ plates (Yan Tai Zi Fu Chemical Group Co., Yantai, People's Republic of China) were used for analytical TLC.

### Fungal Material

The fungal strain *A. sclerotiorum* GDST-2013-0501 was isolated from the inner part of an unidentified sponge GDST-2013-05 collected from the South China Sea, in May 2013. The fungal identification was performed by analysis of its morphological characteristics and ITS region of the rDNA. The sequence data (Supplementary Figures S13, S14) were submitted to the Genbank with accession number MT534582. The strain was deposited in the Key Laboratory of Marine Drugs, the Ministry of Education of China, School of Medicine and Pharmacy, Ocean University of China, Qingdao, PR China.

### Extraction and Isolation

In total, forty 500 ml Erlenmeyer flasks with the fungal strain were cultivated in rice medium (3.6 g of natural sea salt from Yangkou saltern, China; 70 g of rice; 100 ml of H_2_O) for 25 days at room temperature. The ethyl acetate extracts were combined and concentrated to dryness under a vacuum to obtain an ethyl acetate extract (30.0 g). This extract was fractionated by silica gel VLC using a step gradient elution with ethyl acetate–petroleum ether (0–100%) and then with methanol–ethyl acetate (0–100%) to afford five fractions (Fr.1–Fr.5). Fr.4 was separated into four subfractions (W1–W4) by silica gel CC eluting with a step gradient of dichloromethane–methanol (50:1, *v*:*v*). W4 was further purified on an ODS column eluting with 40–60% methanol–H_2_O to produce **6** (8.2 mg) and two subfractions, W41 and W42. W41 was further purified by HPLC (20% methanol–H_2_O) to afford **3** (3.9 mg) and **4** (5.0 mg). W42 was purified by HPLC (20% methanol–H_2_O) to afford **1** (20.0 mg) and **2** (3.0 mg). W3 was further purified by HPLC (20% acetonitrile–H_2_O) to afford **5** (10 mg) and **7** (4.0 mg).

*Speramide C (**1**)*: yellow powder; ${\left[\alpha \right]}_{\text{D}}^{25}$ −29.1 (*c* 0.10, methanol); UV (methanol) λ_max_ (log ε) 206 (0.74), 236 (0.46) 288 (0.19), 336 (0.13) nm; IR (KBr) ν_*max*_ 3,904, 2,927, 2,361, 1,650, 1,522, 1,110 cm^−1^; ^1^H and ^13^C NMR data, Table 1; ESIMS *m/z* 482.2 [M+H]^+^; HRESIMS *m/z* 482.2291 [M+H]^+^ (calcd for C_26_H_32_N_3_O_6_, 482.2286).

**Table 1:** ^1^H (500 MHz) NMR data and ^13^C (125 MHz) NMR data for **1**–**3** in DMSO-*d*_6_.

**Position**	**1**		**2**		**3**	
	**δ_C_, type**	**δ_H_ (*J* in Hz)**	**δ_C_, type**	**δ_H_ (*J* in Hz)**	**δ_C_, type**	**δ_H_ (*J* in Hz)**
2	98.5, C		151.9, C		89.0, C	
3	96.4, C		77.5, C		197.1, C	
3a	119.5, C		128.9, C		113.4, C	
4	125.4, CH	6.98, d (8.0)	123.7, CH	7.36, d (8.0)	125.3, CH	7.28, d (8.3)
5	105.8, CH	6.04, d (8.0)	116.4, CH	6.88, d (8.0)	108.6, CH	6.32, d (8.3)
5a	154.7, C		153.9, C		161.8, C	
7	75.8, C		76.2, C		75.8, C	
8	127.8, CH	5.55, d (9.5)	133.0, CH	5.93, d (10.2)	127.6, CH	5.77, d (10.0)
9	118.6, CH	6.85, d (9.5)	115.4, CH	7.76, d (10.2)	119.5, CH	7.25, d (10.0)
9a	102.8, C		111.4, C		104.7, C	
9b	148.4, C		139.6, C		154.9, C	
10	28.3, CH_3_	1.33, s or 1.34, s	27.5, CH_3_	1.42, s	27.1, CH_3_	1.46, s
11	28.1, CH_3_	1.34, s or 1.33, s	27.4, CH_3_	1.40, s	25.7, CH_3_	1.34, s
12	39.3, CH_2_	2.35, t (12.3) 2.67, dd (12.6, 7.0)	75.6, CH	4.12, s	80.7, CH	6.02, s
13	63.2, CH	4.63, dd (11.8, 7.0)	61.8, C		60.4, C	
14	166.8, C		168.0, C		167.9, C	
16	44.9, CH_2_	3.38, m	43.9, CH_2_	3.40, t (6.4)	43.4, CH_2_	3.20, m; 3.33, m
17	20.9, CH_2_	1.86, m 2.06, m	24.0, CH_2_	2.03, m; 1.84, m	24.1, CH_2_	1.74, m; 1.96, m
18	36.6, CH_2_	2.06, m	28.7, CH_2_	2.55,m; 1.84, m	28.5, CH_2_	2.45, m
19	89.0, C		66.3, C		66.4, C	
20	166.0, C		171.6, C		172.1, C	
22	60.8, CH_2_	3.41, m	30.0, CH_2_	2.03, m; 1.84, m	29.4, CH_2_	1.78, m; 1.98, m
23	91.7, CH	3.57, dd (7.3, 3.0)	49.5, CH	3.53, dd (9.9, 8.0)	41.3, CH	2.88, dd (10.5, 5.9)
24	46.9, C		36.1, C		40.2, C	
25	17.8, CH_3_	0.75, s	13.1, CH_3_	1.15, s	14.7, CH_3_	0.50, s
26	21.8, CH_3_	1.30, s	22.4, CH_3_	1.30, s	20.3, CH_3_	1.06, s
12-OMe			59.5, CH_3_	3.31, s	54.4, CH_3_	3.29, s
1-NH		6.85, s				6.24, s
21-NH				7.85, s		8.08, s
3-OH		4.59, br s		6.26, s		
19-OH		6.74, s				

*3,21-epi-Taichunamide F (**2**)*: yellow powder; ${\left[\alpha \right]}_{\text{D}}^{25}$ −18.3 (*c* 0.10, methanol); UV (methanol) λ_max_ (log ε) 210 (2.28), 258 (0.85) nm; ECD (1.01 mM, methanol) λ_max_ (Δε) 225 (+41.13), 242 (−8.37), 262 (+9.71), 270 (+6.78), 299 (+7.31), 340 (−3.01) nm; IR (KBr) ν_*max*_ 3,821, 2,960, 2,361, 1,699, 1,539, 1,160 cm^−1^; ^1^H and ^13^C NMR data, Table 1; ESIMS *m/z* 494.3 [M+H]^+^; HRESIMS *m/z* 494.2281 [M +H]^+^ (calcd for C_27_H_32_N_3_O_6_, 494.2286).

*2-epi-Amoenamide C (**3**)*: yellow powder; ${\left[\alpha \right]}_{\text{D}}^{20}$ −26.4 (*c* 0.10, methanol); UV (methanol) λ_max_ (log ε) 208 (3.67), 338 (3.23) nm; ECD (1.04 mM, methanol) λ_max_ (Δε) 226 (+37.37), 240 (−19.30), 269 (+26.31), 305 (−4.15), 341 (+13.71) nm; IR (KBr) ν_*max*_ 3,633, 2,922, 2,362, 1,701, 1,540, 1,383, 1,187 cm^−1^; ^1^H and ^13^C NMR data, Table 1; ESIMS *m/z* 478.1159 [M+H]^+^; HRESIMS *m/z* 478.2332 [M +H]^+^ (calcd for C_27_H_32_N_3_O_5_, 478.2336).

### Antibacterial Activity Assays

The antibacterial activity was evaluated by the conventional broth dilution assay (Appendino et al., 2008). Three pathogenic bacterial strains, *Staphyloccocus aureus, S. epidermidis*, and *Escherichia coli*, were used, and ciprofloxacin was used as a positive control.

### DNA Topo I Inhibitory Activity Assay

The Topo I inhibitory activity was measured by assessing the relaxation of supercoiled pBR322 plasmid DNA (Bogurcu et al., 2011; Xin et al., 2017). The gel was stained with GelRed, visualized under UV illumination, and then photographed with a gel imaging system. Camptothecin (CPT) was used as a positive control.

### Cytotoxicity Assays

The cytotoxicity against human promyelocytic leukemia HL-60, human erythroleukemia K562 cell lines, and one human normal liver cell line HL7702 (L-02) was evaluated using the MTT method (Mosmann, 1983). The cytotoxicity against human lung carcinoma A549 cell lines was evaluated using the SRB method (Skehan et al., 1990). Adriamycin was used as a positive control.

### Acetylcholinesterase Inhibitory Assays

The enzyme acetylcholinesterase (AChE) was from *Electrophorus electricus* (electric eel). AChE inhibitory activity was evaluated using the method described by Ellman et al. (1961). Galanthamine hydrobromide was used as a positive control.

## Results

### The Sponge-Derived Fungal Strain *A. sclerotiorum* GDST-2013-0501

The sponge-derived fungal strain, *A. sclerotiorum* GDST-2013-0501, was cultured with rice medium in 30 Erlenmeyer flasks at room temperature for 30 days. The fermented rice medium was extracted three times with ethyl acetate. The combined ethyl acetate layers were evaporated under reduced pressure to give the ethyl acetate extract (30 g). By repeating column chromatography (CC) over silica gel, octadecylsilyl (ODS), Sephadex LH-20, and semi-preparative HPLC, seven compounds were obtained, including speramide C (**1**), 3,21-*epi*-taichunamide F (**2**), and 2-*epi*-amoenamide C (**3**), together with four known compounds, notoamide F (**4**) (Tsukamoto et al., 2008), 6-*epi*-notoamide R (**5**) (Cai et al., 2013), notoamide G (**6**) (Tsukamoto et al., 2008), and *epi*-deoxybrevianamide E (**7**) (Sobolevskaya et al., 2013) (Figure 2).

### Structure Elucidation

**Figure 3:**
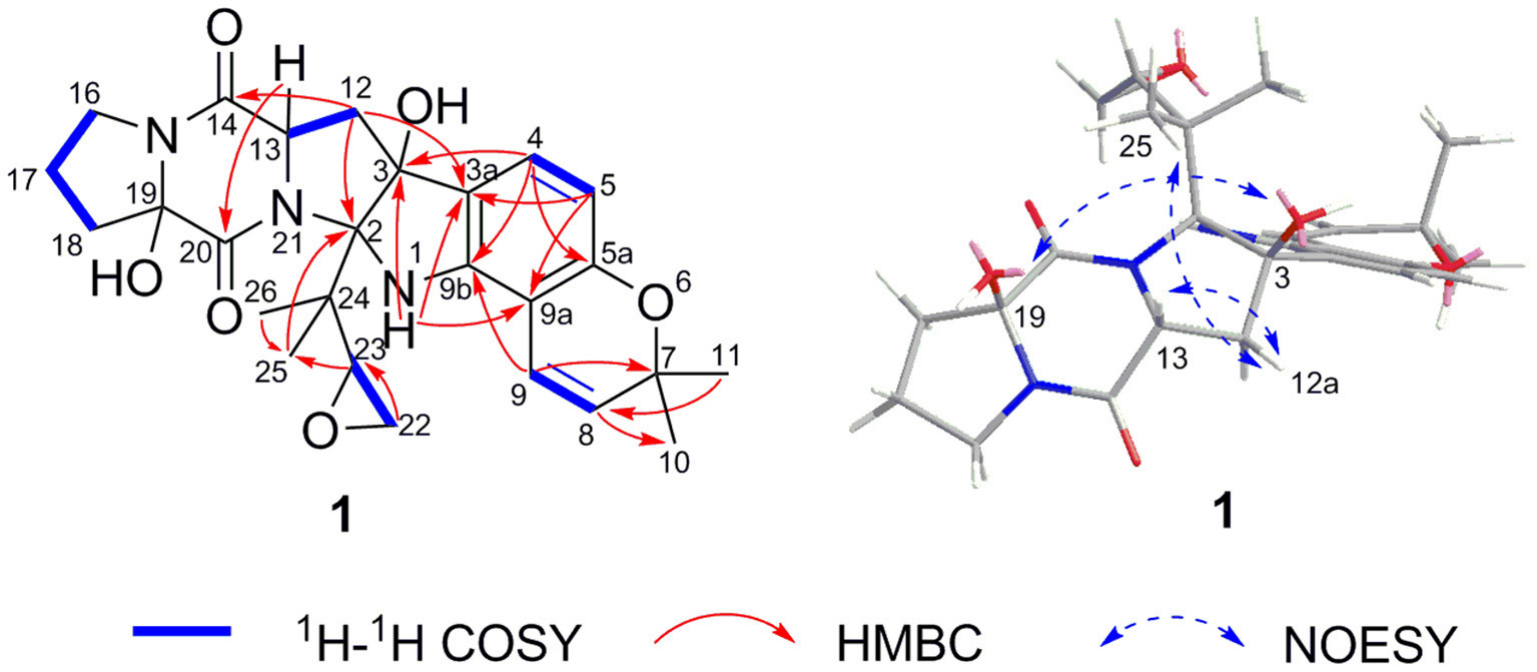
Key 2D NMR correlations for **1**.

Speramide C (**1**), a yellow amorphous powder, was established to have a molecular formula of C_26_H_31_N_3_O_6_ by HRESIMS from the [M + H]^+^ ion at *m*/*z* 482.2291 (482.2286, calcd for C_26_H_32_N_3_O_6_) with 13 degrees of unsaturation. The ^1^H NMR data of **1** (Table 1) included the resonances of two aromatic protons (δ_H_ 6.98, d, *J* = 8.0 Hz; 6.04, d, *J* = 8.0 Hz), two olefinic protons (δ_H_ 6.85, d, *J* = 9.5 Hz and 5.55, d, *J* = 9.5 Hz), and four methyl groups (δ_H_ 1.34, s; 1.33, s; 1.30, s and 0.75, s) as well as signals attributable to five methylene and two methine groups. The ^13^C NMR data (Table 1) displayed 26 carbon resonances, including two amide carbonyl carbons (at δ_C_ 166.8 and 166.0), six aromatic carbons (four quaternary), two olefinic carbons, four methyl groups, five methylenes, two methines, and five sp^3^ quaternary carbons. The ^1^H and ^13^C NMR data suggested that compound **1** is a 2,5-DKP, structurally closely related to speramide B, which was previously isolated from the fungus *A. ochraceus*. Further analyses of the ^1^H and ^13^C NMR data (Supplementary Table S1) found that **1** differed from speramide B mainly at the isopentenyl side chain. The difference between these two compounds was that an ethylene oxide group (one methylene group at δ_C_ 60.8 and one methine group at δ_C_ 91.7) in **1** instead of a double bond (two olefinic carbons at δ_C_ 110.8 and 145.6) in speramide B (Supplementary Table S1) (Chang et al., 2016). The ethylene oxide group linked to the isopentenyl side chain (C-23) was assigned by the HMBC correlations from Me-25 to C-2, from Me-26 to C-25, and from H-23 to C-25, along with the COSY correlation of H_2_-22/H-23 (Figure 3). Therefore, the planar structure of **1** was elucidated.

**Figure 4:**
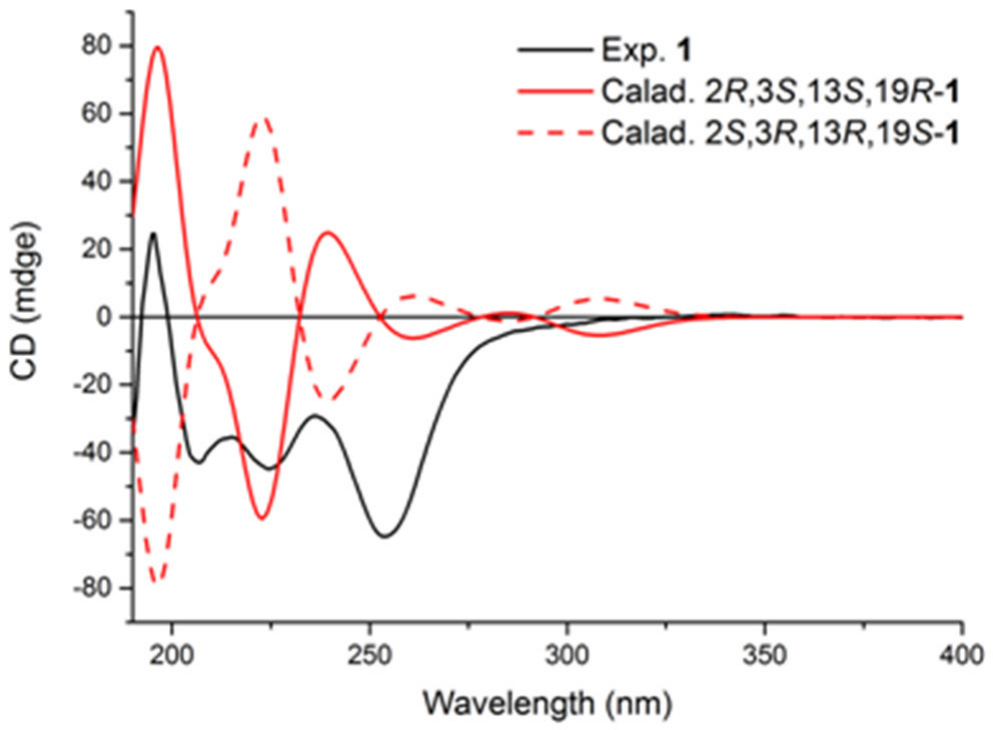
Experimental and calculated ECD spectra of compound **1**.

The relative configurations of C-2, C-3, C-13, and C-19 of **1** were determined by NOESY correlations (Figure 3). The NOESY correlations of 3-OH/19-OH, H-13/H-12a, and Me-25/H-12a revealed these protons were cofacial. However, the relative configuration of C-23 was left unassigned due to the flexibility of the isopentenyl side chain. To define the absolute configurations of C-2, C-3, C-13, and C-19, an ECD calculation was performed at the B3LYP/6-31G(d,p) level using a time-dependent density functional theory (TD–DFT) method by the SpecDis 1.6 program. Finally, the absolute configuration of (2*R*,3*S*,13*S*,19*R*)-**1** was defined on account of the good agreement between the calculated ECD curve and the experimental one (Figure 4).

**Figure 5:**
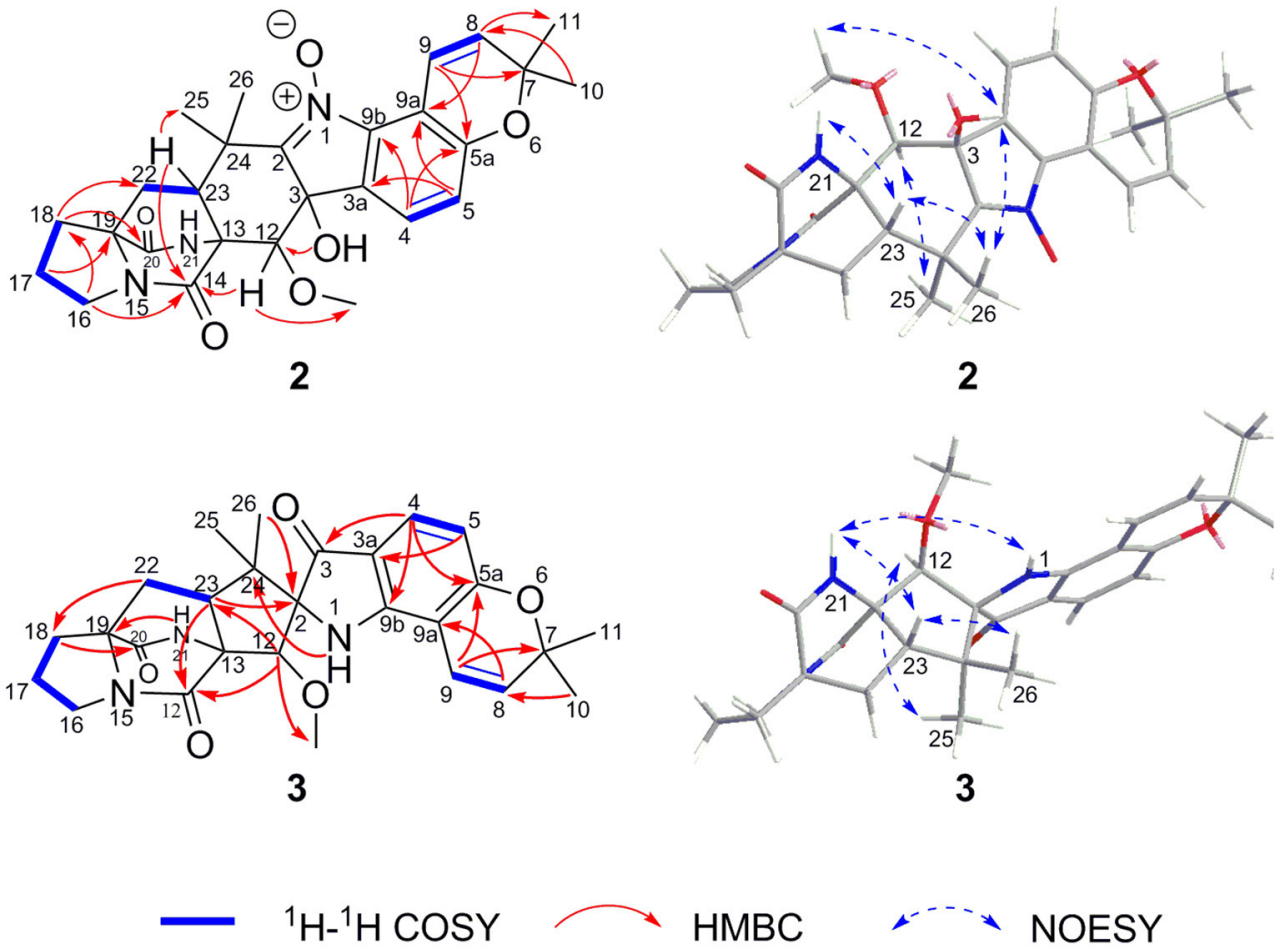
Key 2D NMR correlations for **2** and **3**.

3,21-*Epi*-taichunamide F (**2**), obtained as a yellow powder, was found to have the chemical formula C_27_H_31_N_3_O_6_, which indicates 14 degrees of unsaturation. The ^1^H NMR data of **2** (Table 1) displayed the typical pattern of a prenylated indole alkaloid skeleton with the presence of one 1,2,3,4-tetrasubstituted benzene unit (H-4, δ_H_ 7.36, *J* = 8.0 Hz; H-5, δ_H_ 6.88, *J* = 8.0 Hz), one *Z*-configured double bond (H-9, δ_H_ 7.76, d, *J* = 10.2 Hz and H-8, δ_H_ 5.93, d, *J* = 10.2 Hz), four methyl groups (δ_H_ 1.15, 1.30, 1.40, and 1.42), one methoxy group (δ_H_ 3.31), and four sp^3^-hybridized methylene groups. Detailed analysis of the 1D, 2D NMR (Table 1 and Figure 5), and MS data revealed that the planar structure of **2** is the same as that of the prenylated 2,5-DKP taichunamide F, which was previously isolated from the fungus *A. taichungensis* (Kagiyama et al., 2016). The most obvious differences between **2** and taichunamide F were the ^1^H and ^13^C NMR chemical shifts of 2, 12, and 23-positions (Supplemtary Table S2), indicating that their absolute configurations of them might be different.

**Figure 6:**
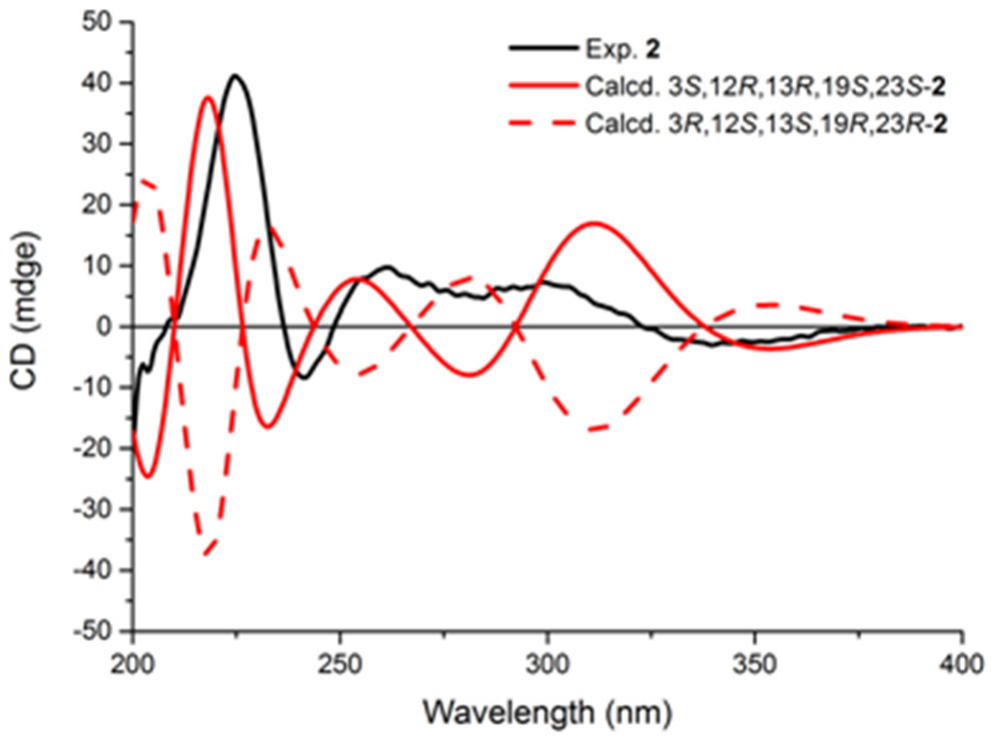
Experimental and calculated ECD spectra of compound **2**.

The stereochemistry of **2** was established by NOESY experiments in conjunction with the ECD spectra and ECD calculations. The relative configuration of **2** was elucidated by a NOESY experiment (Figure 5). The NOESY correlations of 21-NH/H-23, H-23/Me-26, Me-26/3-OH, and 3-OH/12-OMe along with the interaction between H-12 and Me-25 indicated the α-orientation of 21-NH, H-23, and 3-OH, and β-orientation of H-12. The absolute configurations of the bridgehead carbons C-13 and C-19 of **2** were established by a CD exciton chirality method as reported by Williams et al. (1989). The Cotton effects (CEs) at 200–250 nm, which arises from an n–π^*^ transition of the amide bonds, is a reliable diagnostic method to discern the absolute stereochemistry of bicyclo[2.2.2]diazaoctane diketopiperazine (Herscheid et al., 1979; Williams et al., 1989; Kato et al., 2007). The closely resembled CEs at 200−250 nm of compound **2** (Figure 6) (positive CEs: Δε +41.13 at 225 nm and negative CEs: Δε −8.37 at 242 nm) to taichunamide F indicated that the absolute configurations of bicyclo[2.2.2]-diazaoctane of **2** were 13*R*,19*S*. According to the relative configurations elucidated earlier, the absolute configuration of **2** was ascertained as 3*S*, 12*R*, 13*R*, 19*S*, and 23*S*, which was different from the reported taichunamide F. To further confirm the absolute configuration of **2**, the calculated ECD was performed using TD–DFT at the B3LYP/6-311+G (d, p) level in methanol by the CPCM polarizable conductor calculation model. Conformational searches were carried out using the Merck Molecular Force Field (MMFF) by means of the Spartan's 10 software to acquire meaningful conformers for 3*S*,12*R*,13*R*,19*S*,23*S*-**2**. The theoretical ECD spectrum for 3*R*,12*S*,13*S*,19*R*, and 23*R*-**2** was obtained by directly reversing the spectrum of 3*S*, 12*R*, 13*R*, 19*S*, and 23*S*-**2**. The calculated curve of 3*S*, 12*R*, 13*R*, 19*S*, and 23*S*-**2** matched with its experimental ECD spectrum (Figure 6). As a result, we confirmed that **2** is the C-3 epimer of taichunamide F, and the absolute configuration of **2** is 3*S*, 12*R*, 13*R*, 19*S*, and 23*S*.

2-*epi*-Amoenamide C (**3**) was also obtained as a yellow powder and its molecular formula was determined to be C_27_H_31_N_3_O_5_ based on HRESIMS, requiring an index of hydrogen deficiency of 14. The ^1^H NMR data of **3** (Table 1) displayed two aromatic protons (δ_H_ 6.32, H-5, d, *J* = 8.3 Hz; 7.28, H-4, d, *J* = 8.3 Hz) and typical signals of one *cis-*1,2-disubstituted double bond (δ_H_ 7.25, H-9, d, *J* = 10.0 Hz; 5.77, H-8, d, *J* = 10.0 Hz). Detailed analysis of ^1^H NMR data indicated that **3** also belongs to the prenylated indole alkaloid. The ^13^C NMR (Table 1) along with its HSQC spectrum of **3** exhibited 27 carbon resonances, including three carbonyls (one ketocarbonyl carbon at δ_C_ 197.1 and two amide carbonyl carbons at δ_C_ 172.1 and 167.9) corresponding with ν_max_ 1,701 cm^−1^ in the IR spectrum, six aromatic carbons, two olefinic carbons, one methoxy group, four methyl groups, four methylene groups, two methine carbons, and five sp^3^ quaternary carbons. Further analyses of the ^1^H–^1^H COSY and HMBC spectra of **3** confirmed the presence of a bicyclo[2.2.2]diazaoctane core including a proline (Figure 5). Moreover, these spectroscopic features of **3** were very similar to those of amoenamide C, previously isolated from the fungus *Fusarium sambucinum* (Zhang et al., 2019). The most obvious differences in ^1^H and ^13^C NMR spectra between **3** and aminoamide C were the chemical shifts of C-2, C-12, and C-23-positions (Supplementary Table S3), indicating that the absolute configurations of them might be different. The aforementioned data, as well as the HMBC correlations from H-12 to C-14 and C-23, from H-23 to C-2 and C-14, from NH-1 to C-24, and from H-4 to C-3 and C-9b (Figure 5), indicated the presence of an aza-spiro structure fused to the bicyclo-[2,2,2]diazaoctane system *via* C-13 and C-23. Therefore, the planar structure of **3** was elucidated.

The relative configuration of **3** was ascertained by the examination of its NOESY spectrum (Figure 5). The obvious NOESY correlations between 21-NH/H-23, H-23/Me-26, and 21-NH/1-NH suggested that these protons should be cofacial and α-oriented, which established the relative configurations of C-23 and the spiro-carbon C-2. Correspondingly, the NOESY correlations between Me-25/H-12 indicated that these protons should be β-oriented. Thus, the relative configurations of **3** were determined, which differed from those of aminoamide C.

**Figure 7:**
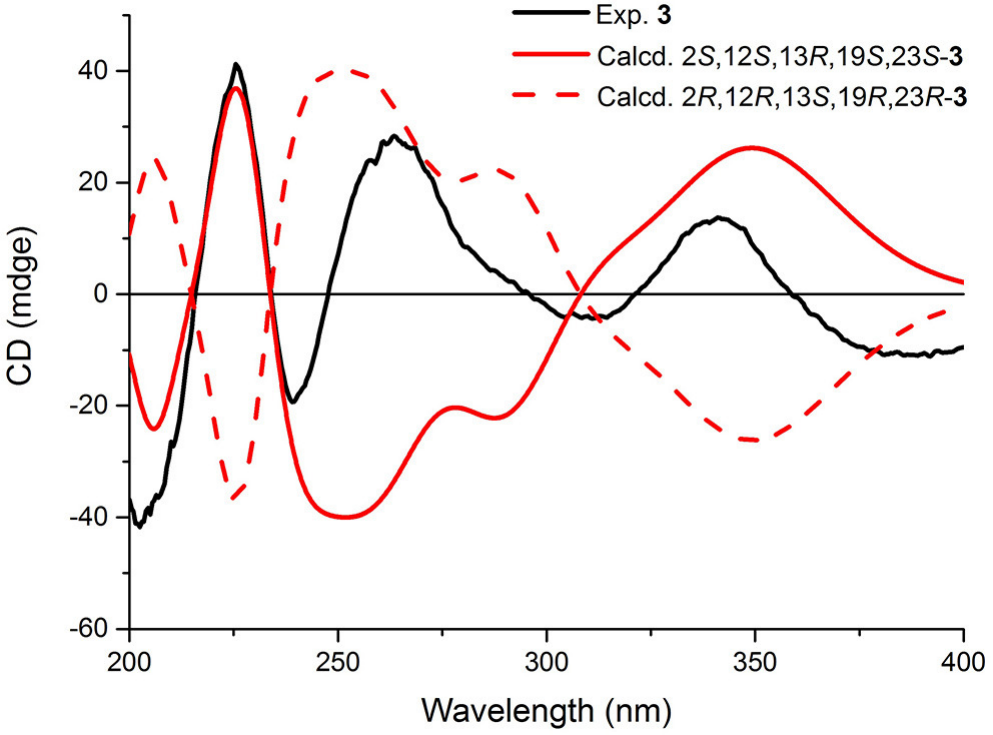
Experimental and calculated ECD spectra of compound **3**.

In the experimental ECD spectrum of **3**, the positive CEs at 226 nm (Δε +37.37) and negative CEs at 240 nm (Δε −19.30) (Figure 7) compared well with the relevant regions in that of (+)-brevianamide B (Williams et al., 1989). These results combined the relative configurations allowed the assignment of the absolute configuration of **3** as 2*S*, 12*S*, 13*R*, 19*S*, and 23*S*, which was a C-2 epimer of the reported aminoamide C (Zhang et al., 2019). This result was further confirmed by ECD calculation with the calculated ECD of 2*S*, 12*S*, 13*R*, 19*S*, and 23*S*-**3** matched with that of the experimental spectrum (Figure 7).

The known compounds notoamide F (**4**) (Tsukamoto et al., 2008), 6-*epi*-notoamide R (**5**) (Cai et al., 2013), notoamide G (**6**) (Tsukamoto et al., 2008), and *epi*-deoxybrevianamide E (**7**) (Sobolevskaya et al., 2013) were identified by the comparison of their spectroscopic data, ECD and specific optical rotation data with those in the literature.

### Bioassays of Compounds

All of the isolated compounds were subjected to a panel of bioassays to evaluate their potential activities. These included evaluation of antibacterial activity toward *Staphyloccocus aureus, S. epidermidis*, and *Escherichia coli*, cytotoxic activity against three human tumor cell lines, including human promyelocytic leukemia HL-60, human lung carcinoma A-549, chronic leukemia K562 cell lines and one human normal liver cell line HL7702 (L-02), DNA topoisomerase I (Topo I), and acetylcholinesterase (AChE) inhibitory activities. Compound **4** displayed moderate Topo I inhibitory activity with the MIC value of 100.0 μM (the positive control CPT with the MIC value of 40.0 μM) (Supplementary Figure S26), whereas **4** showed no significant cytotoxic activity against all of the human tumor cell lines and even the normal cell lines. The aforementioned results suggested that the low cytotoxic or non-cytotoxic compound **4** might possess Topo I inhibitory activity and deserves to be further studied for the rational drug design of antitumor agents. Besides, **4** also showed moderate antibacterial activity against *S. epidermidis* with the MIC value of 12.5 μM (the positive control ciprofloxacin with the MIC value of 3.13 μM). These data suggest that the antibacterial activity of **4** against *S. epidermidis* may be mediated through Topo I inhibition, which should be further confirmed by testing for bacterial DNA topoisomerase I activity.

## Conclusion

The 2,5-DKP derivatives cyclized from tryptophan and proline could mostly be classified as prenylated indole alkaloids possessing a simple piperazine-2,5-dione core or a complicated bicyclo[2.2.2]diazaoctane ring and several isoprene units (Greshock et al., 2008). The present study revealed that the new compound **1** and the known analog **7** belong to prenylated indole alkaloids, possessing a simple piperazine-2,5-dione core and one or two isoprene units, while the new compounds **2** and **3** along with **three** known analogs (**4**–**6**) belong to the substructure type of bicyclo[2.2.2]diazaoctane ring-containing prenylated indole alkaloids, which usually have two or three isoprene units (Borthwick and Costa, 2017). Compound **1** is the first reported prenylated indole alkaloid with an ethylene oxide ring at the isopentenyl side chain.

## Data Availability Statement

The original contributions presented in the study are included in the article/Supplementary Material, further inquiries can be directed to the corresponding author/s.

## Author Contributions

C-YuW and C-LS participated in conceiving the idea and revising the manuscript. C-YiW contributed to the fermentation, extraction, isolation, and manuscript preparation. X-HL, Y-YZ, X-YN, and Y-HZ contributed to the bioactivity test. C-YiW, X-MF, and XL contributed to the data analysis, writing, revising, and proofreading of the manuscript. All the authors read and approved the final version of the manuscript.

## Funding

This work was supported by the National Key Research and Development Program of China (No. 2018YFC0310900); the National Natural Science Foundation of China (Nos. 41830535 and U1706210); the Program of Open Studio for Druggability Research of Marine Natural Products, Pilot National Laboratory for Marine Science and Technology (Qingdao, China) directed by Kai-Xian Chen and Yue-Wei Guo; and the Taishan Scholars Program, China.

## Conflict of Interest

The authors declare that the research was conducted in the absence of any commercial or financial relationships that could be construed as a potential conflict of interest.

## Publisher's Note

All claims expressed in this article are solely those of the authors and do not necessarily represent those of their affiliated organizations, or those of the publisher, the editors and the reviewers. Any product that may be evaluated in this article, or claim that may be made by its manufacturer, is not guaranteed or endorsed by the publisher.

## Supplementary Material

^1^H NMR, ^13^C NMR, HMQC, COSY, HMBC, NOESY, and MS spectra of **1**, **2**, and **3**; ECD calculation details of **1** and **2**; and DNA Topo I inhibitory activity of the isolated compounds.
