# Supplementary material for: 2,5-Diketopiperazines From a Sponge-Derived Fungus Aspergillus sclerotiorum
Source: Front Microbiol. 2022 May 20;13:808532. doi: 10.3389/fmicb.2022.808532 (PMC9164150; doi:10.3389/fmicb.2022.808532)
Supplement: Supplementary file 1 [file Data_Sheet_1.docx]

**2,5-Diketopiperazines from a Sponge-Derived Strain of the Fungus *Aspergillus sclerotiorum***

Chao-Yi Wang ^1,2^, Xiao-Han Liu^1,2^, Yao-Yao Zheng ^1,2^, Xing-Yan Ning^1,2^, Ya-Hui Zhang^1,2^, Xiu-Mei Fu ^1,2^, Xin Li ^1,2^, Chang-Lun Shao ^1,2^* and Chang-Yun Wang ^1,2,3^*

^1^ Key Laboratory of Marine Drugs, The Ministry of Education of China, School of Medicine and Pharmacy, Ocean University of China, Qingdao 266003, People’s Republic of China

^2^ Laboratory for Marine Drugs and Bioproducts, Qingdao National Laboratory for Marine Science and Technology, Qingdao 266237, People’s Republic of China

^3^ Institute of Evolution & Marine Biodiversity, Ocean University of China, Qingdao 266003, People’s Republic of China

*** Correspondence:**

Chang-Yun Wang

changyun@ouc.edu.cn

Chang-Lun Shao

shaochanglun@ouc.edu.cn

**List of Supporting Information**

**Figure S1.** ^1^H NMR (500 MHz, DMSO-*d*_6_) spectrum of compound **1**

**Figure S2.** Partial ^1^H NMR (500 MHz, DMSO-*d*_6_) spectrum of compound **1**

**Figure S3.** ^13^C NMR (125 MHz, DMSO-*d*_6_) spectrum of compound **1**

**Figure S4.** HSQC (DMSO-*d*_6_) spectrum of compound **1**

**Figure S5.** ^1^H−^1^H COSY (DMSO-*d*_6_) spectrum of compound **1**

**Figure S6.** HMBC (DMSO-*d*_6_) spectrum of compound **1**

**Figure S7.** NOESY (DMSO-*d*_6_) spectrum of compound **1**

**Figure S8.** HRESIMS spectrum of compound **1**

**Figure S9.** ^1^H NMR (500 MHz, DMSO-*d*_6_) spectrum of compound **2**

**Figure S10.** ^13^C NMR (125 MHz, DMSO-*d*_6_) spectrum of compound **2**

**Figure S11.** HSQC (DMSO-*d*_6_) spectrum of compound **2**

**Figure S12.** ^1^H−^1^H COSY (DMSO-*d*_6_) spectrum of compound **2**

**Figure S13.** HMBC (DMSO-*d*_6_) spectrum of compound **2**

**Figure S14.** NOESY (DMSO-*d*_6_) spectrum of compound **2**

**Figure S15.** Partial NOESY (DMSO-*d*_6_) spectrum of compound **2**

**Figure S16.** HRESIMS spectrum of compound **2**

**Figure S17.** ^1^H NMR (500 MHz, DMSO-*d*_6_) spectrum of compound **3**

**Figure S18.** ^13^C NMR (125 MHz, DMSO-*d*_6_) spectrum of compound **3**

**Figure S19.** HSQC (DMSO-*d*_6_) spectrum of compound **3**

**Figure S20.** ^1^H−^1^H COSY (DMSO-*d*_6_) spectrum of compound **3**

**Figure S21.** HMBC (DMSO-*d*_6_) spectrum of compound **3**

**Figure S22.** NOESY (DMSO-*d*_6_) spectrum of compound **3**

**Figure S23.** Partial NOESY (DMSO-*d*_6_) spectrum of compound **3**

**Figure S24.** HRESIMS spectrum of compound **3**

**Figure S25.** ECD spectrum of compound **7**.

**Figure S26.** DNA Topo I inhibitory activity of the isolated compounds

**Table S1.** Comparison of chemical shift differences between **1** and the reported speramide B

**Table S2.** Comparison of chemical shift differences between **2** and the reported taichunamide F

**Table S3.** Comparison of chemical shift differences between **3** and the reported amoenamide C

**ECD calculation details of compound 1**

**ECD calculation details of compound 2**

**ECD calculation details of compound 3**

**The rDNA-ITS sequence of the fungal strain *Aspergillus sclerotiorum* GDST-2013-0501**

**

**

**Figure S1.** ^1^H NMR (500 MHz, DMSO-*d*_6_) spectrum of compound **1**

**

**

**Figure S2.** Partial ^1^H NMR (500 MHz, DMSO-*d*_6_) spectrum of compound **1**

**

**

**Figure S3.** ^13^C NMR (125 MHz, DMSO-*d*_6_) spectrum of compound **1**

**

**

**Figure S4.** HSQC (DMSO-*d*_6_) spectrum of compound **1**

**

**

**Figure S5.** ^1^H−^1^H COSY (DMSO-*d*_6_) spectrum of compound **1**

**

**

**Figure S6.** HMBC (DMSO-*d*_6_) spectrum of compound **1**

**

**

**Figure S7.** NOESY (DMSO-*d*_6_) spectrum of compound **1**

**

**

**Figure S8.** HRESIMS spectrum of compound **1**


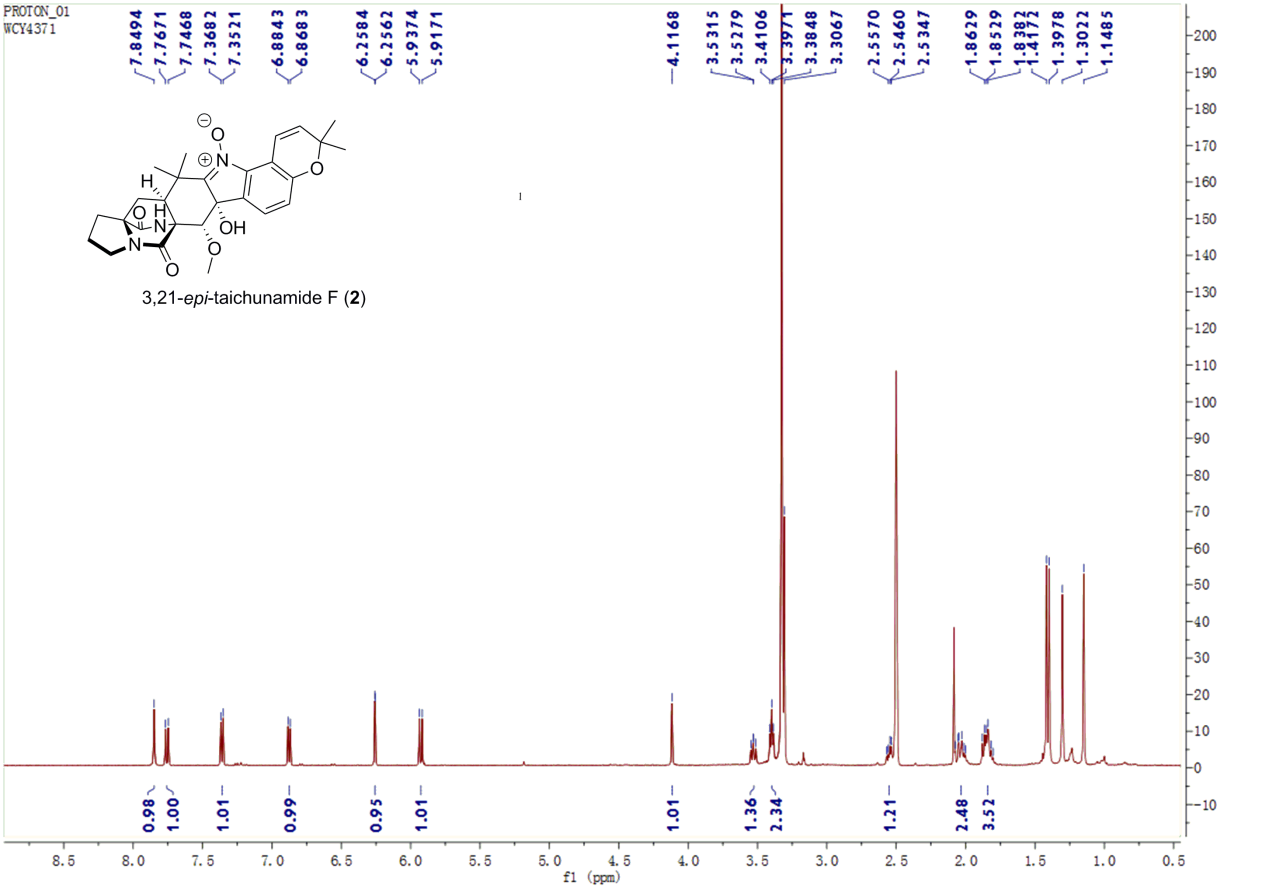


**Figure S9.** ^1^H NMR (500 MHz, DMSO-*d*_6_) spectrum of compound **2**

**
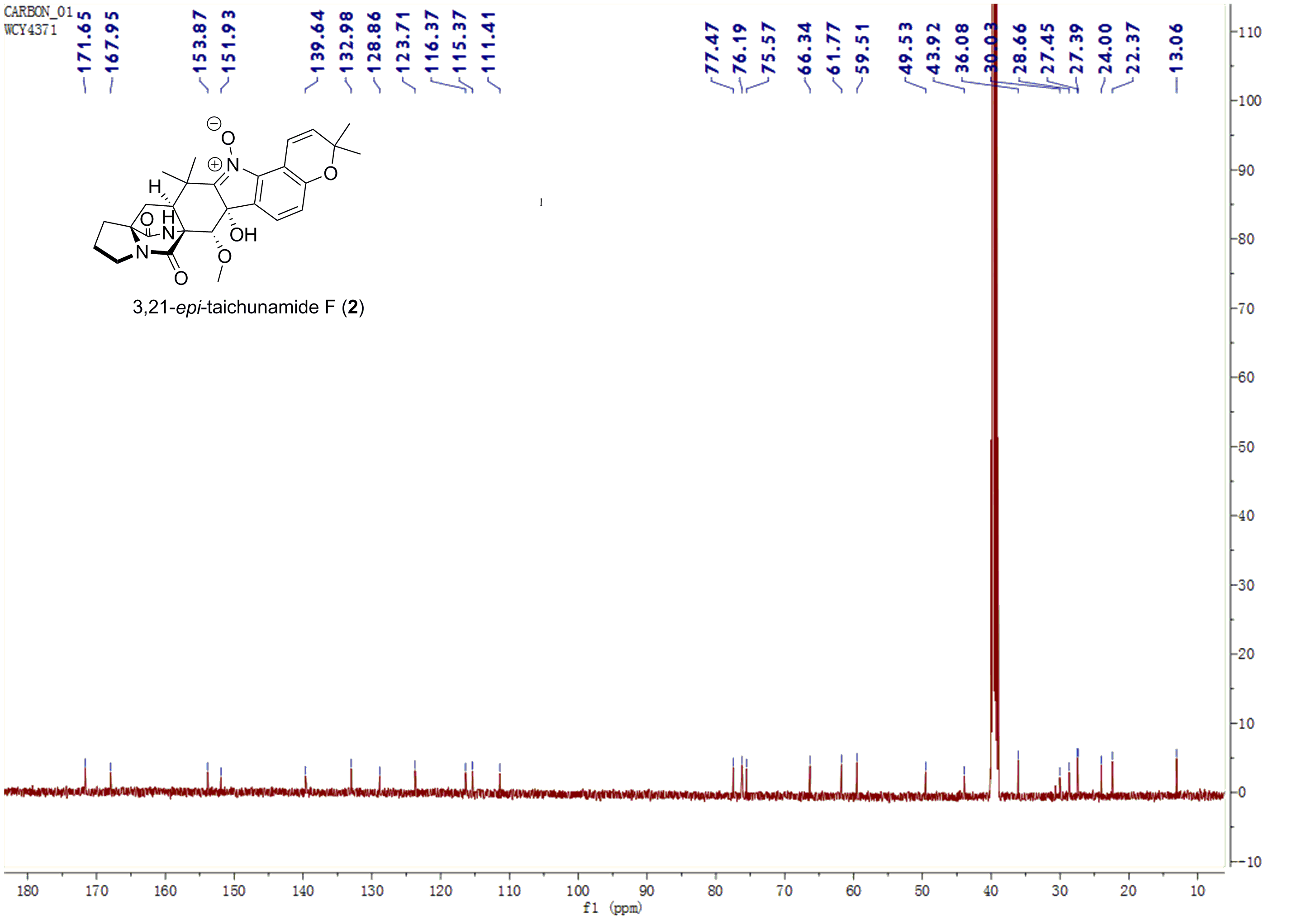
**

**Figure S10.** ^13^C NMR (500 MHz, DMSO-*d*_6_) spectrum of compound **2**

**
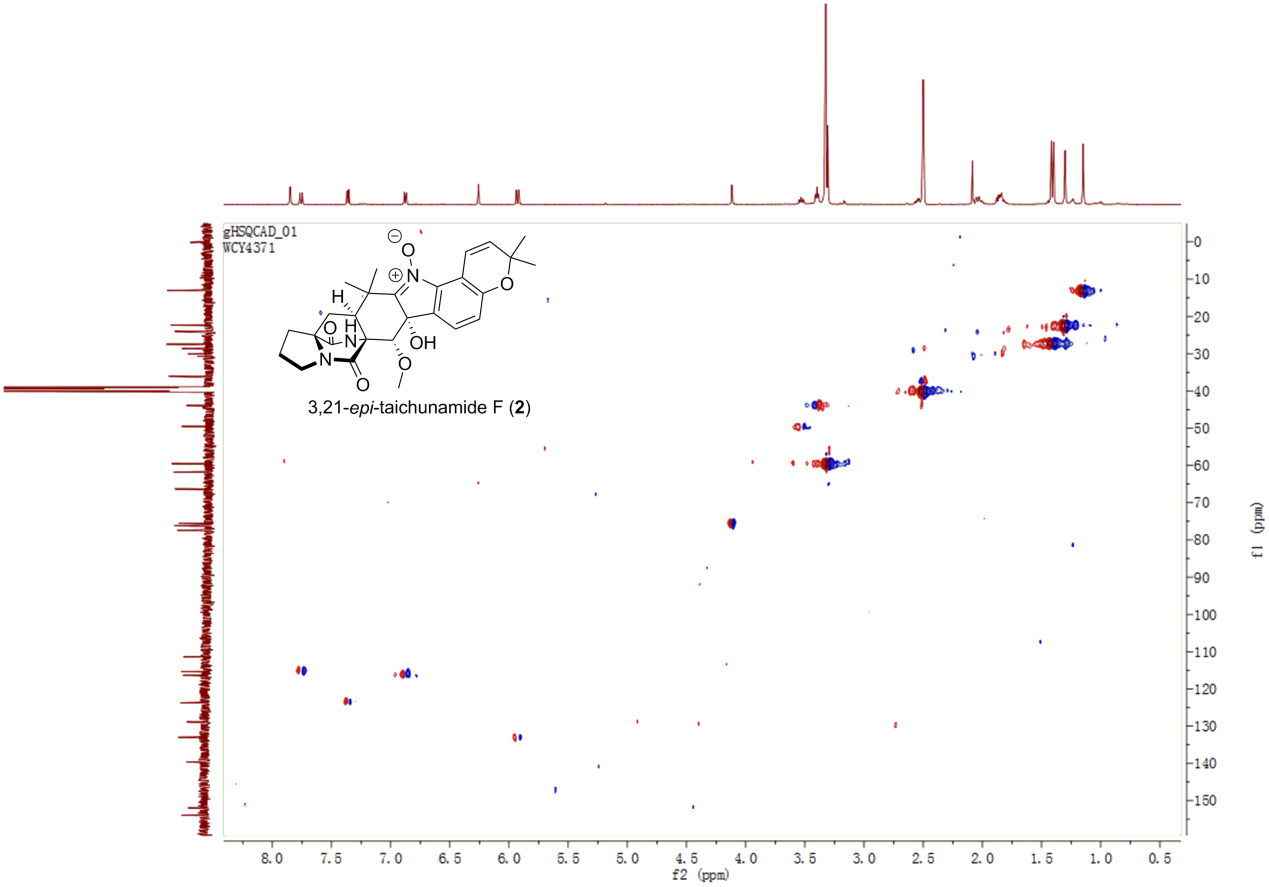
**

**Figure S11.** HSQC (DMSO-*d*_6_) spectrum of compound **2**


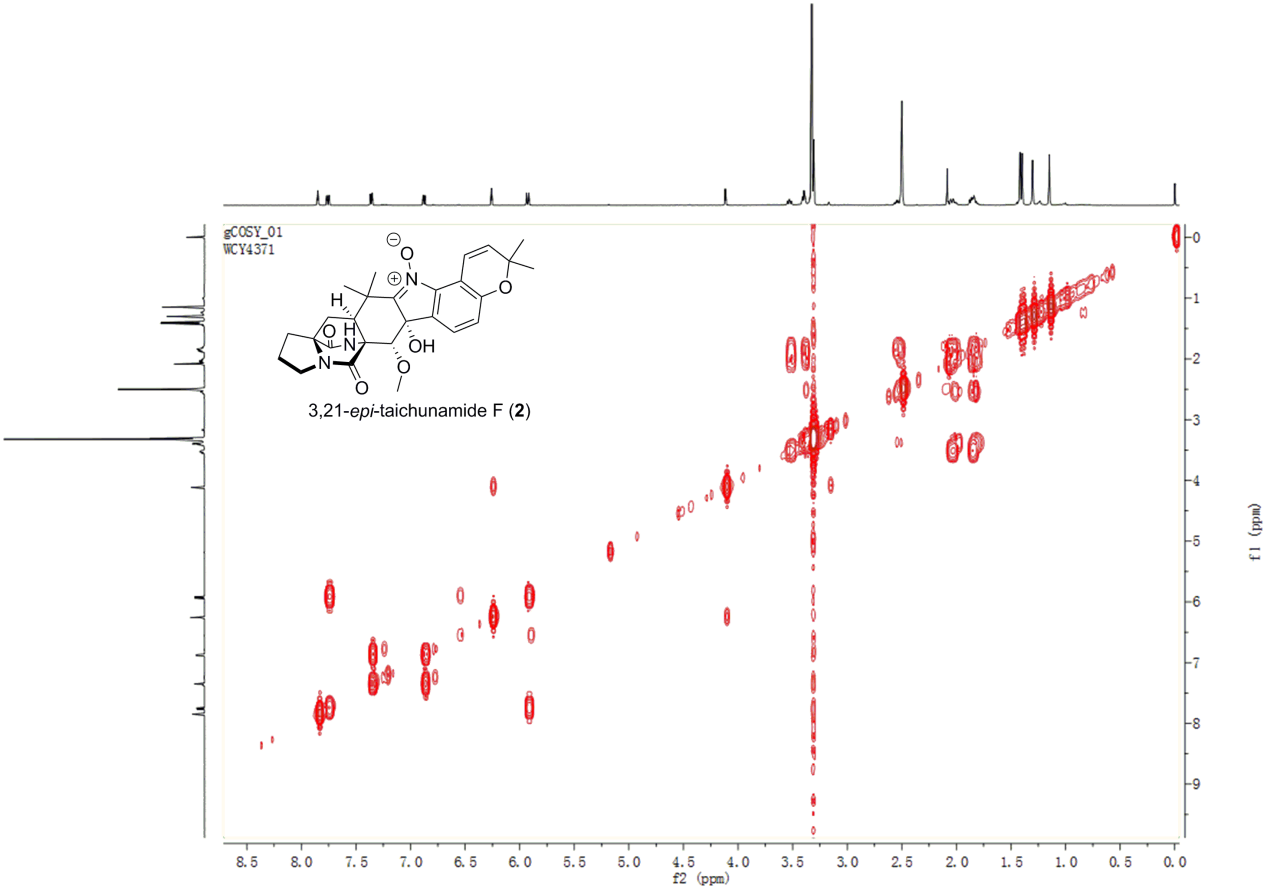


**Figure S12.** ^1^H−^1^H COSY (DMSO-*d*_6_) spectrum of compound **2**


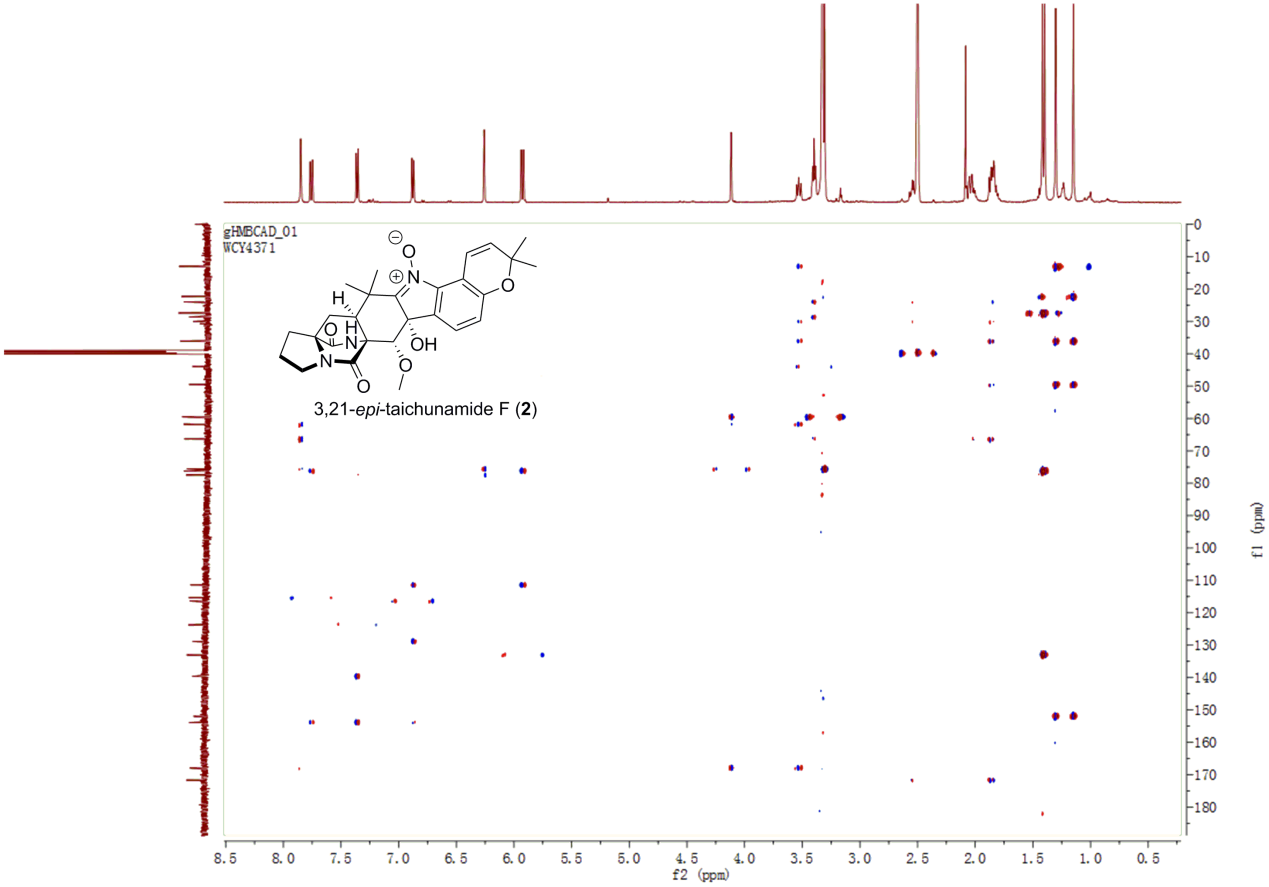


**Figure S13.** HMBC (DMSO-*d*_6_) spectrum of compound **2**

**
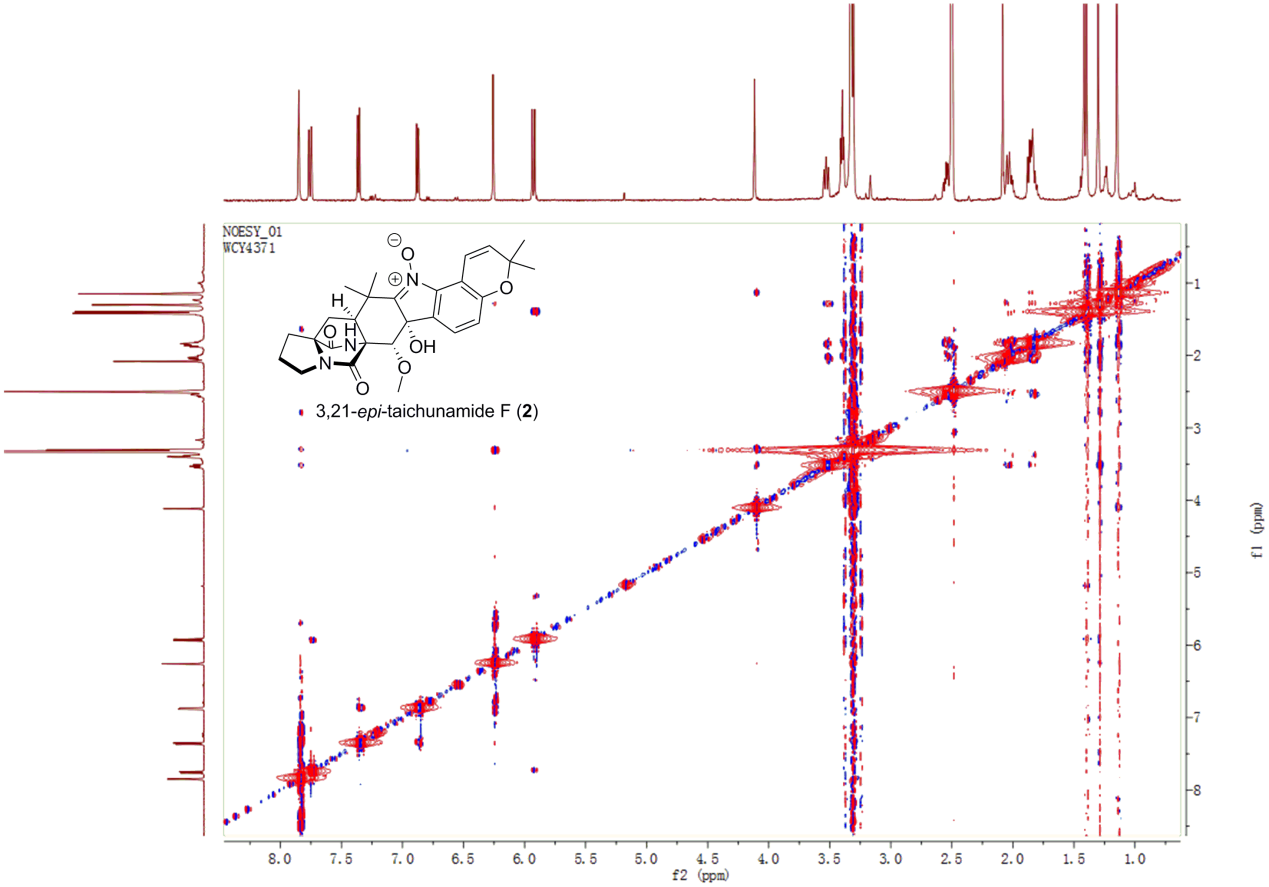
**

**Figure S14.** NOESY (DMSO-*d*_6_) spectrum of compound **2**


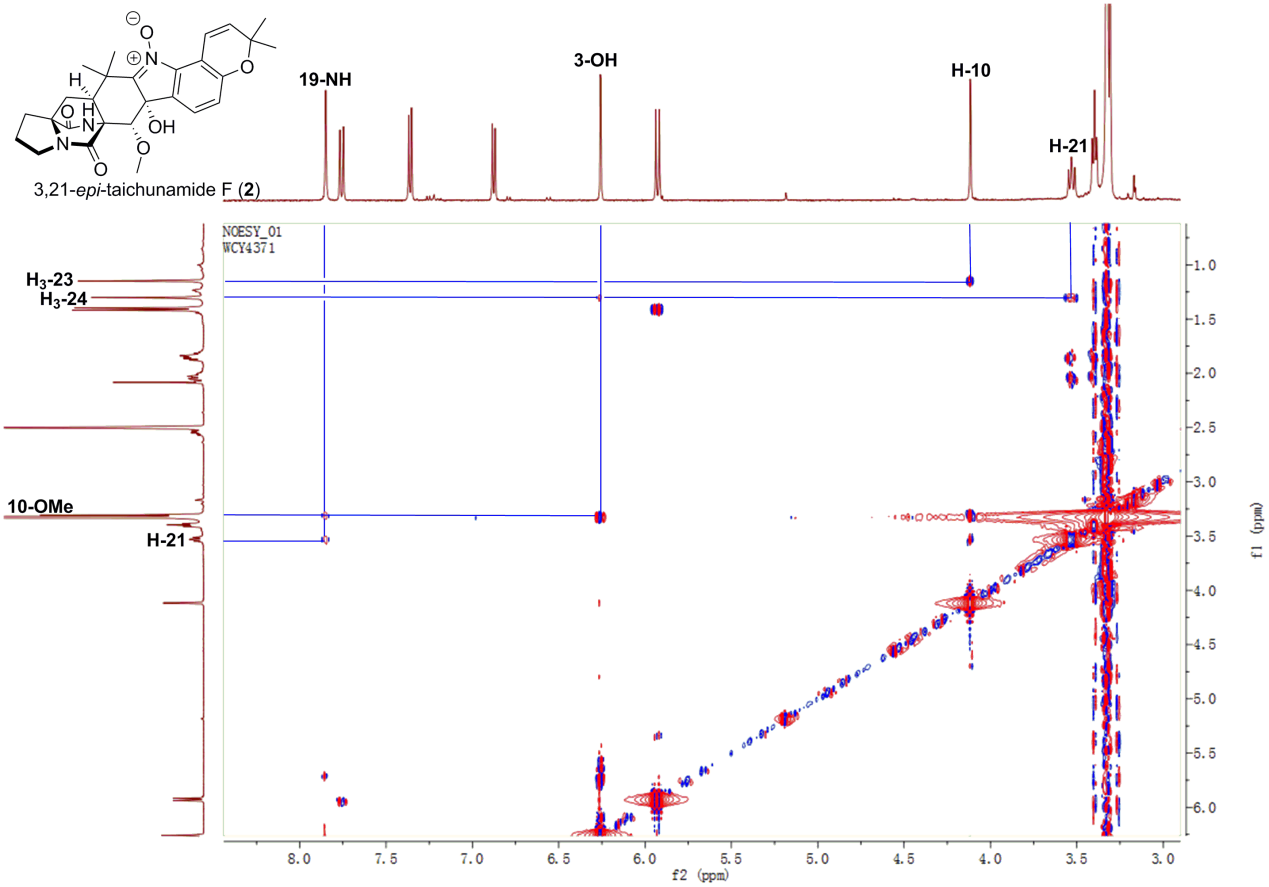


**Figure S15.** Partial NOESY (DMSO-*d*_6_) spectrum of compound **2**

**
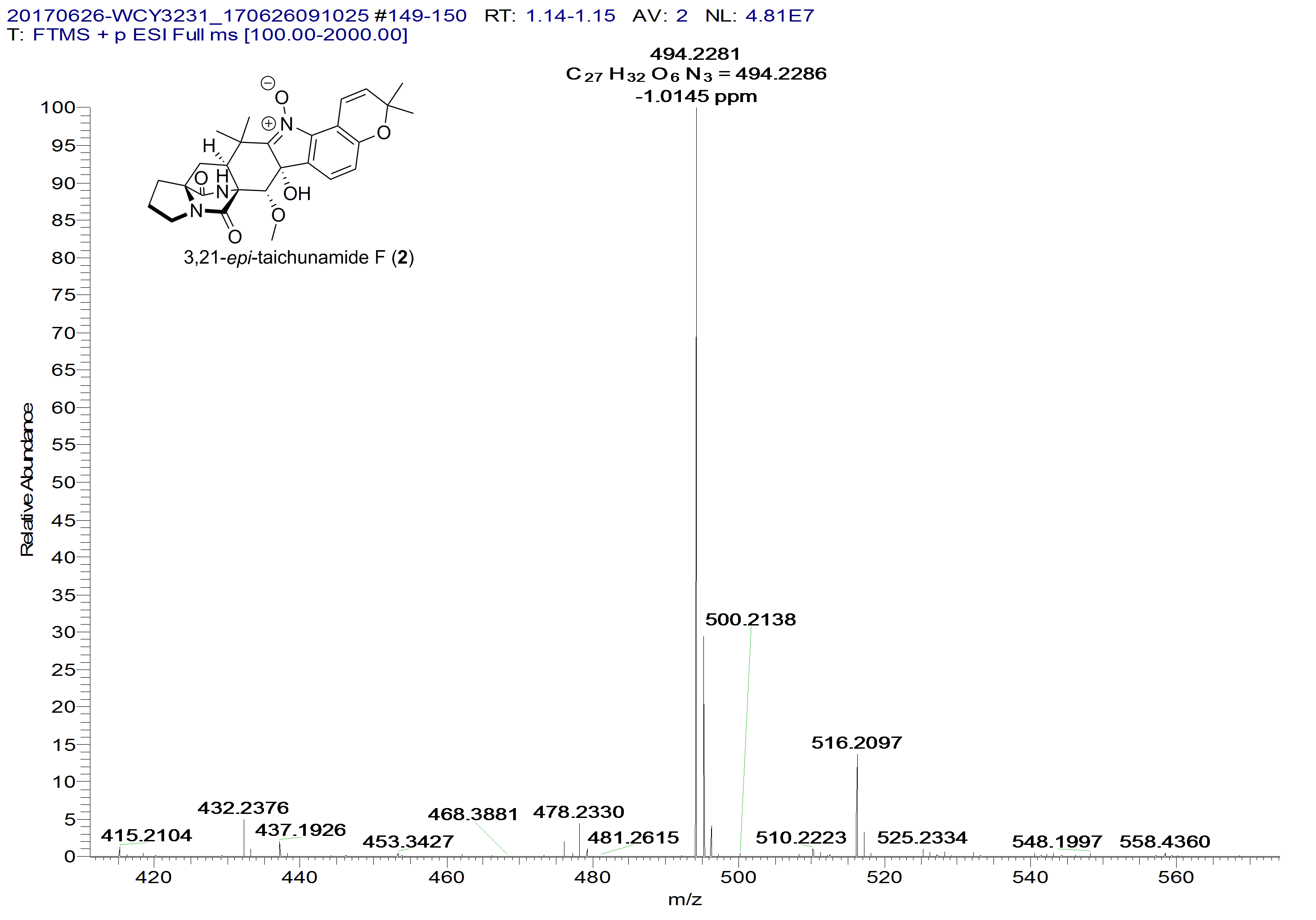
**

**Figure S16.** HRESIMS spectrum of compound **2**


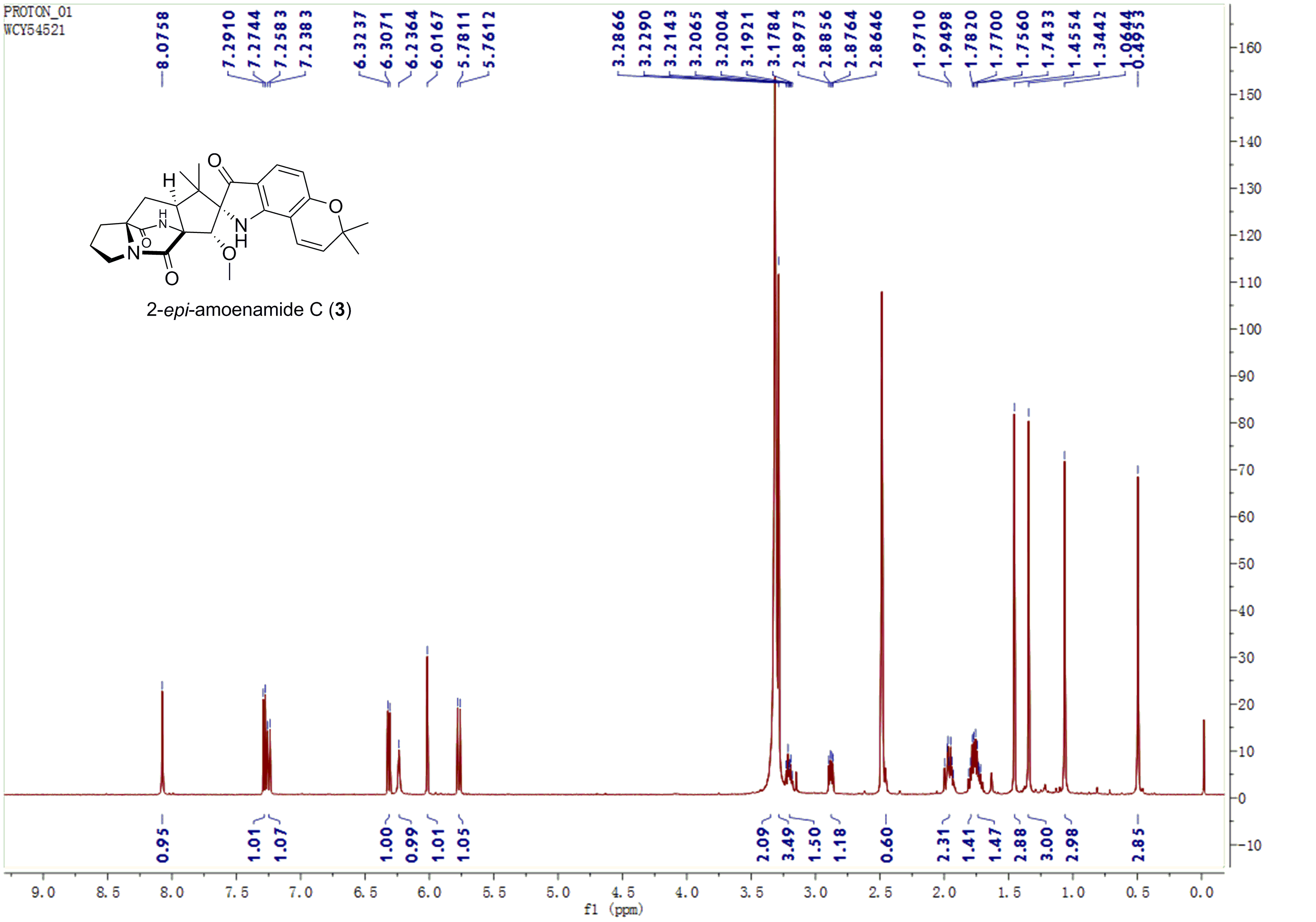


**Figure S17.** ^1^H NMR (500 MHz, DMSO-*d*_6_) spectrum of compound **3**


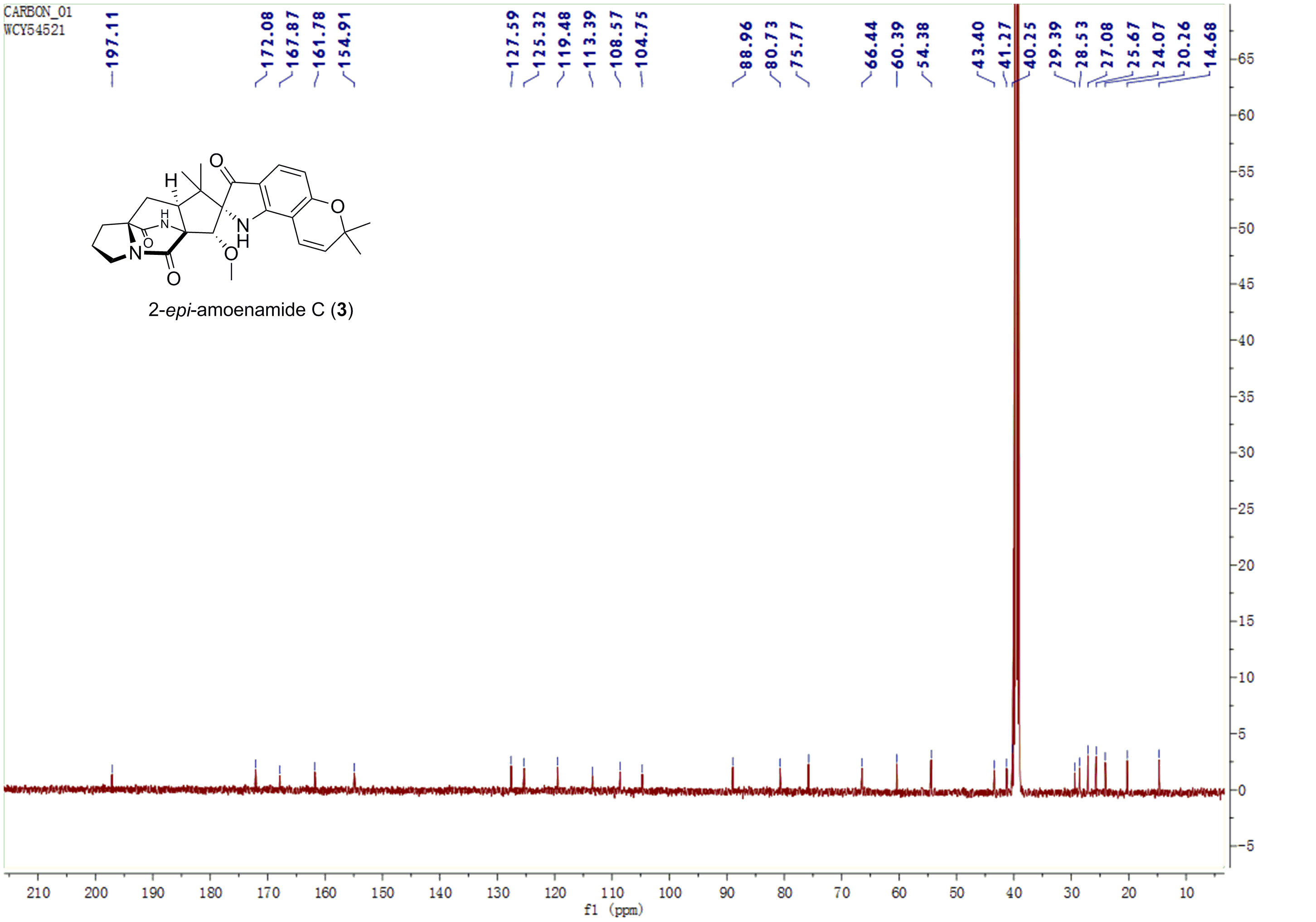


**Figure S18.** ^13^C NMR (125 MHz, DMSO-*d*_6_) spectrum of compound **3**


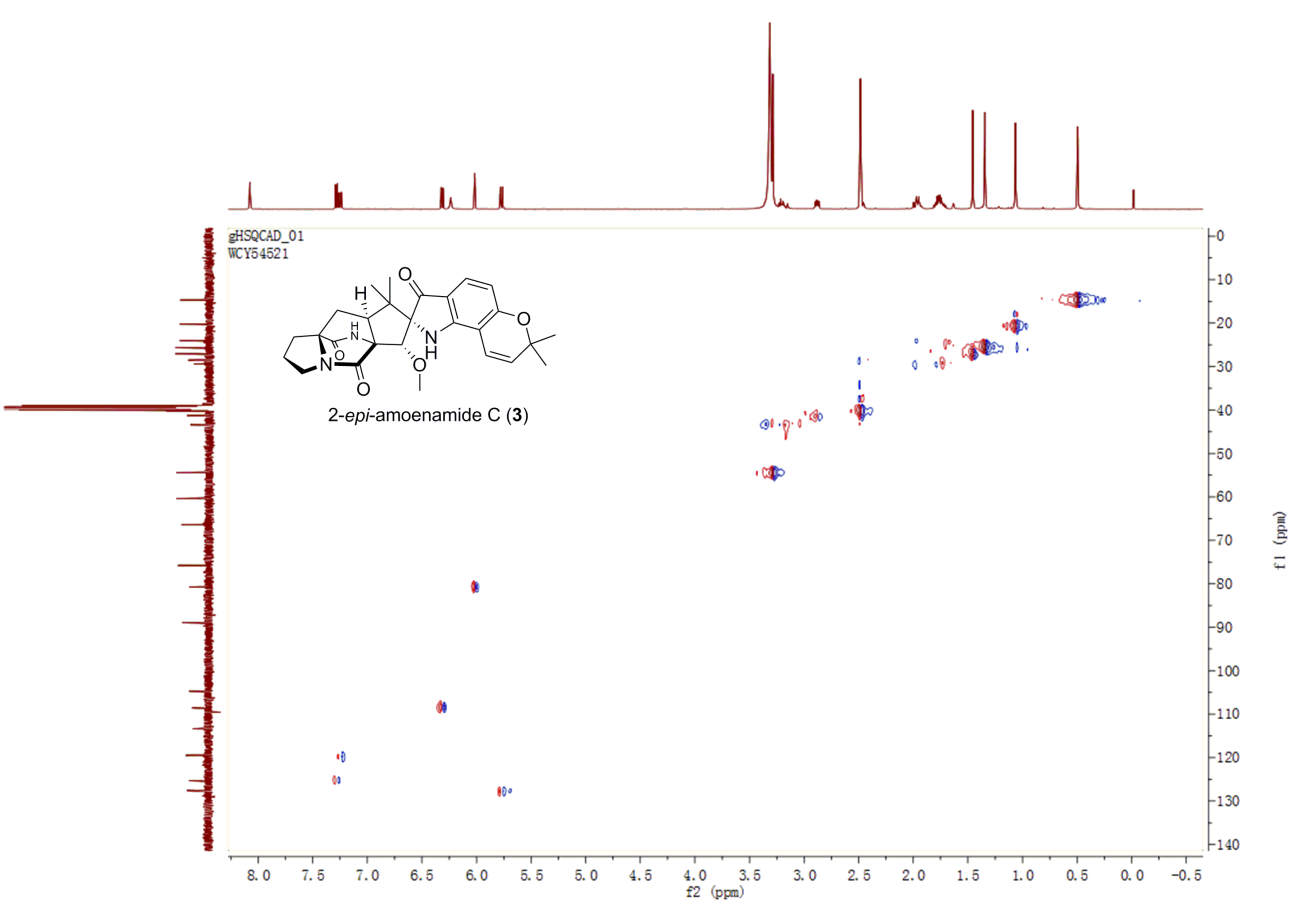


**Figure S19.** HSQC (DMSO-*d*_6_) spectrum of compound **3**


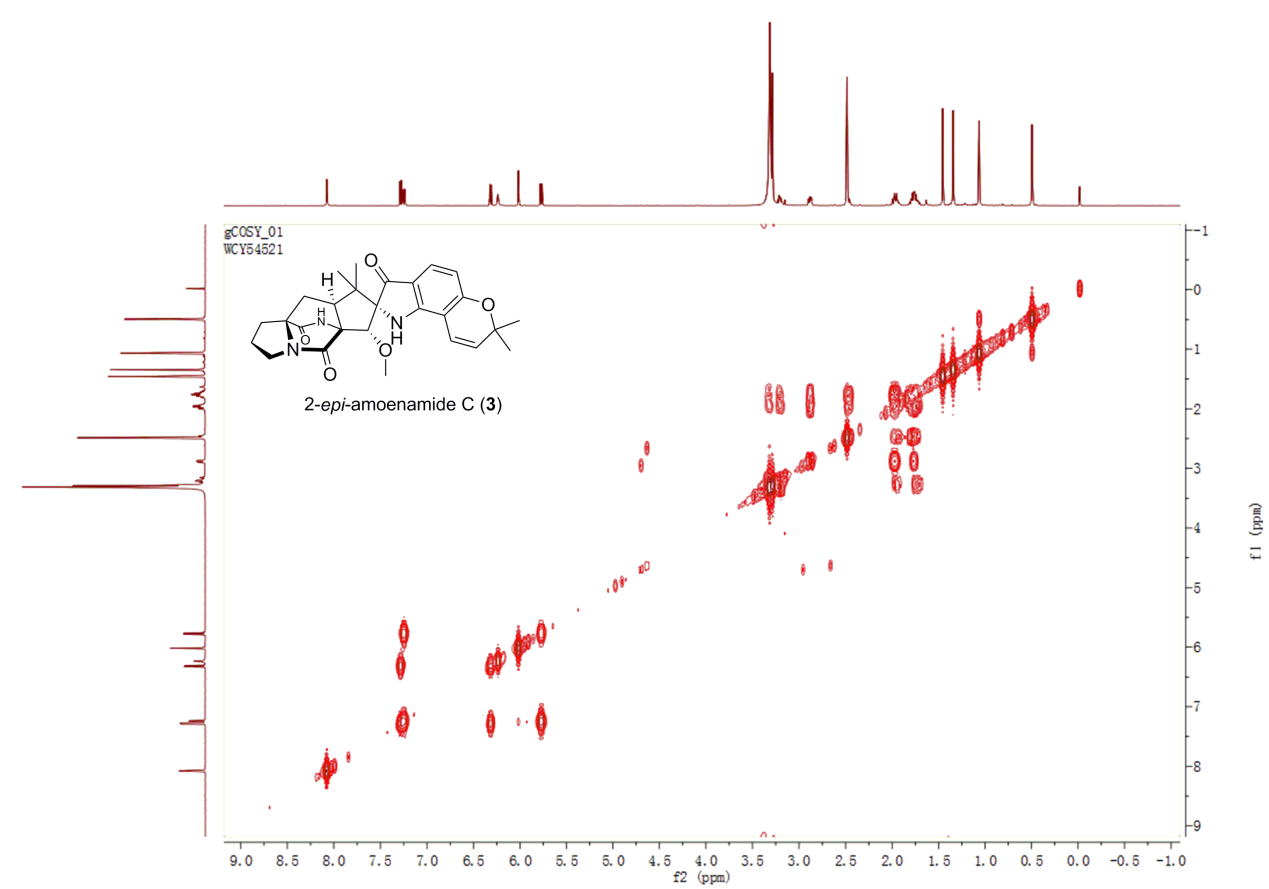


**Figure S20.** ^1^H−^1^H COSY (DMSO-*d*_6_) spectrum of compound **3**


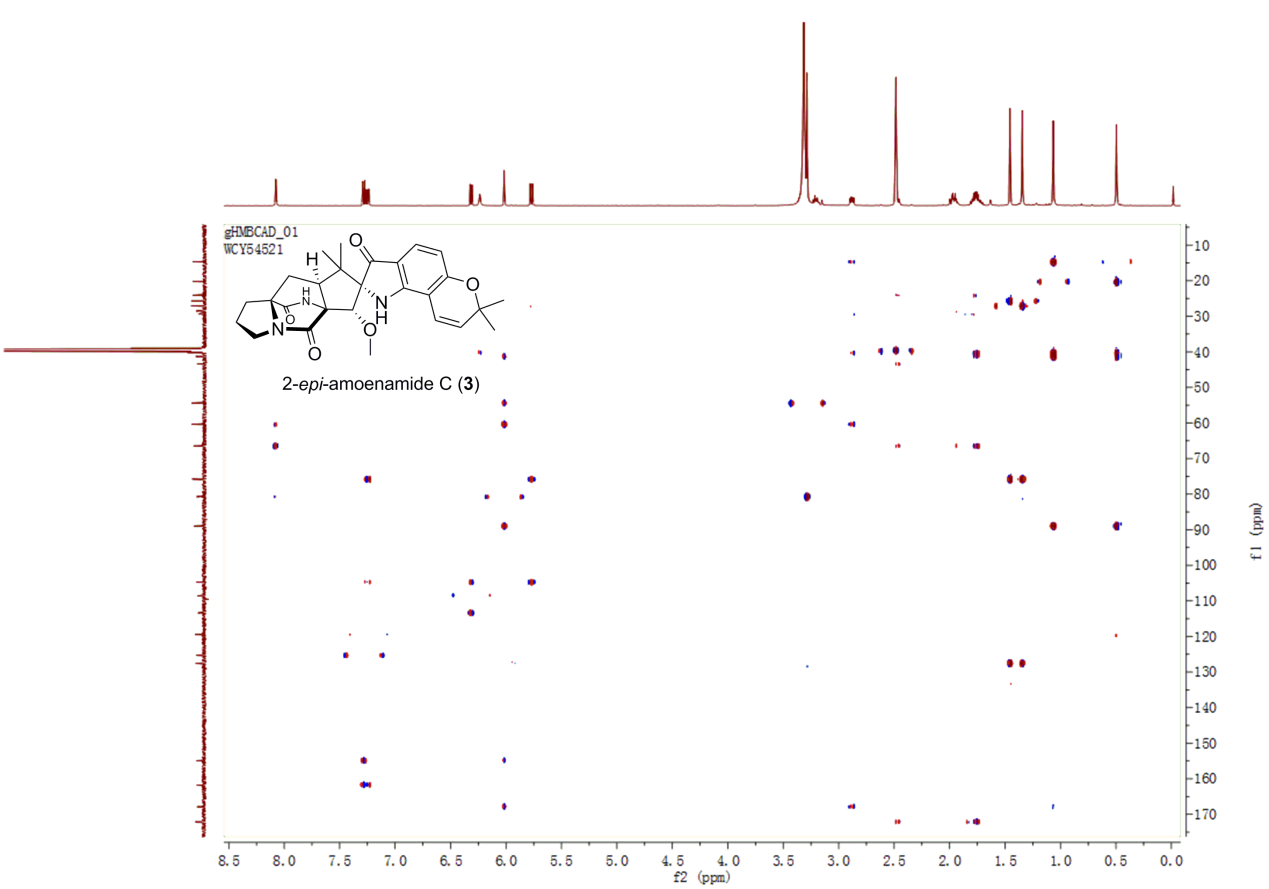


**Figure S21.** HMBC (DMSO-*d*_6_) spectrum of compound **3**

**
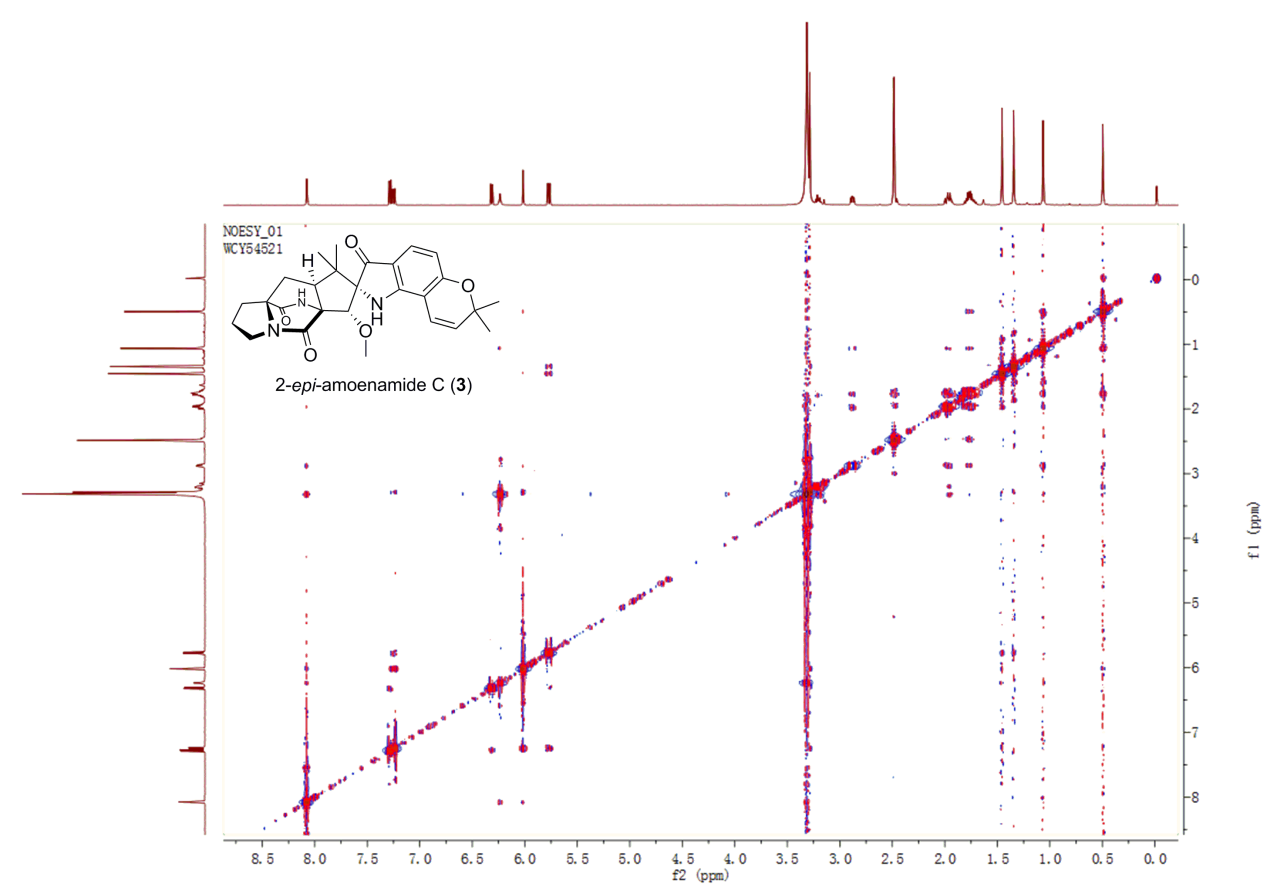
**

**Figure S22.** NOESY (DMSO-*d*_6_) spectrum of compound **3**

**
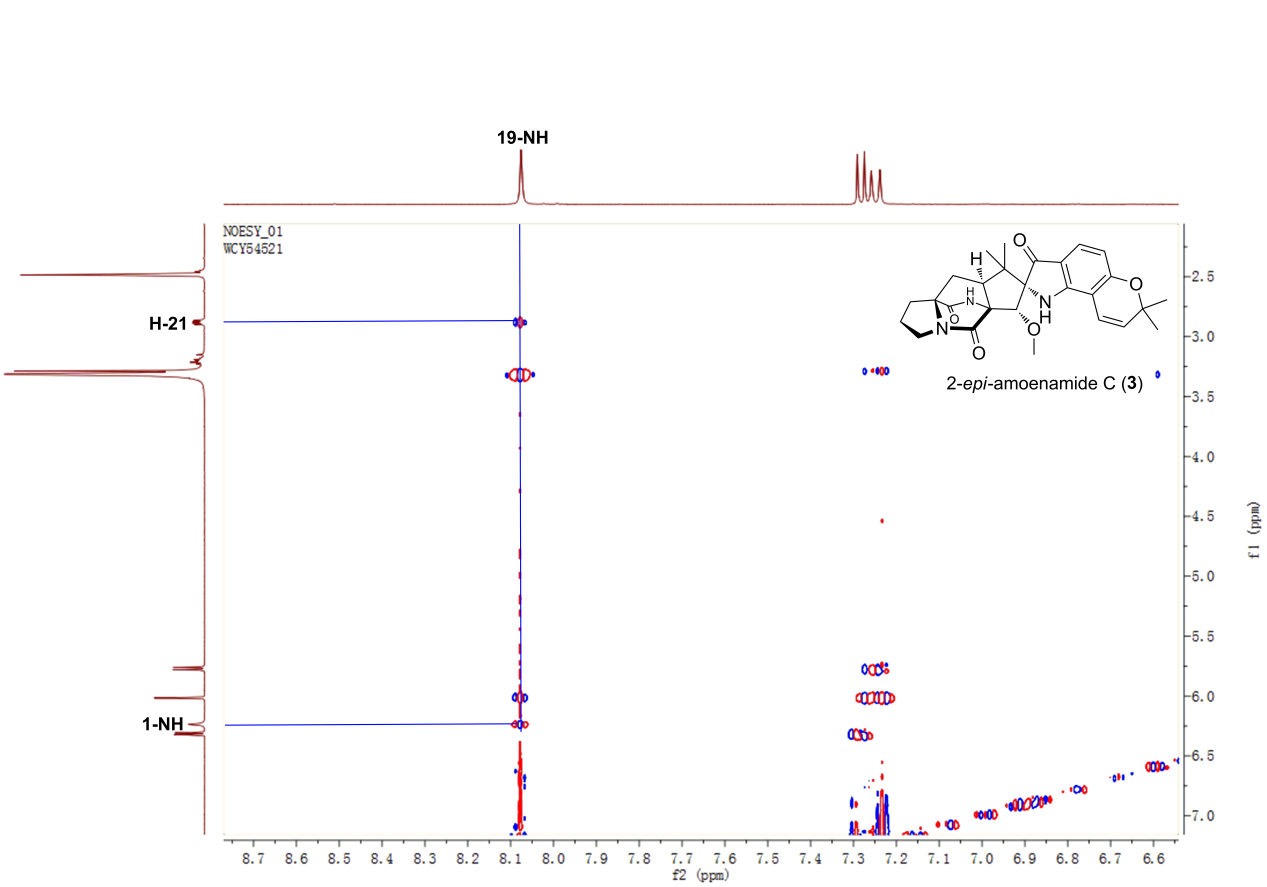
**

**Figure S23.** Partial NOESY (DMSO-*d*_6_) spectrum of compound **3**

**
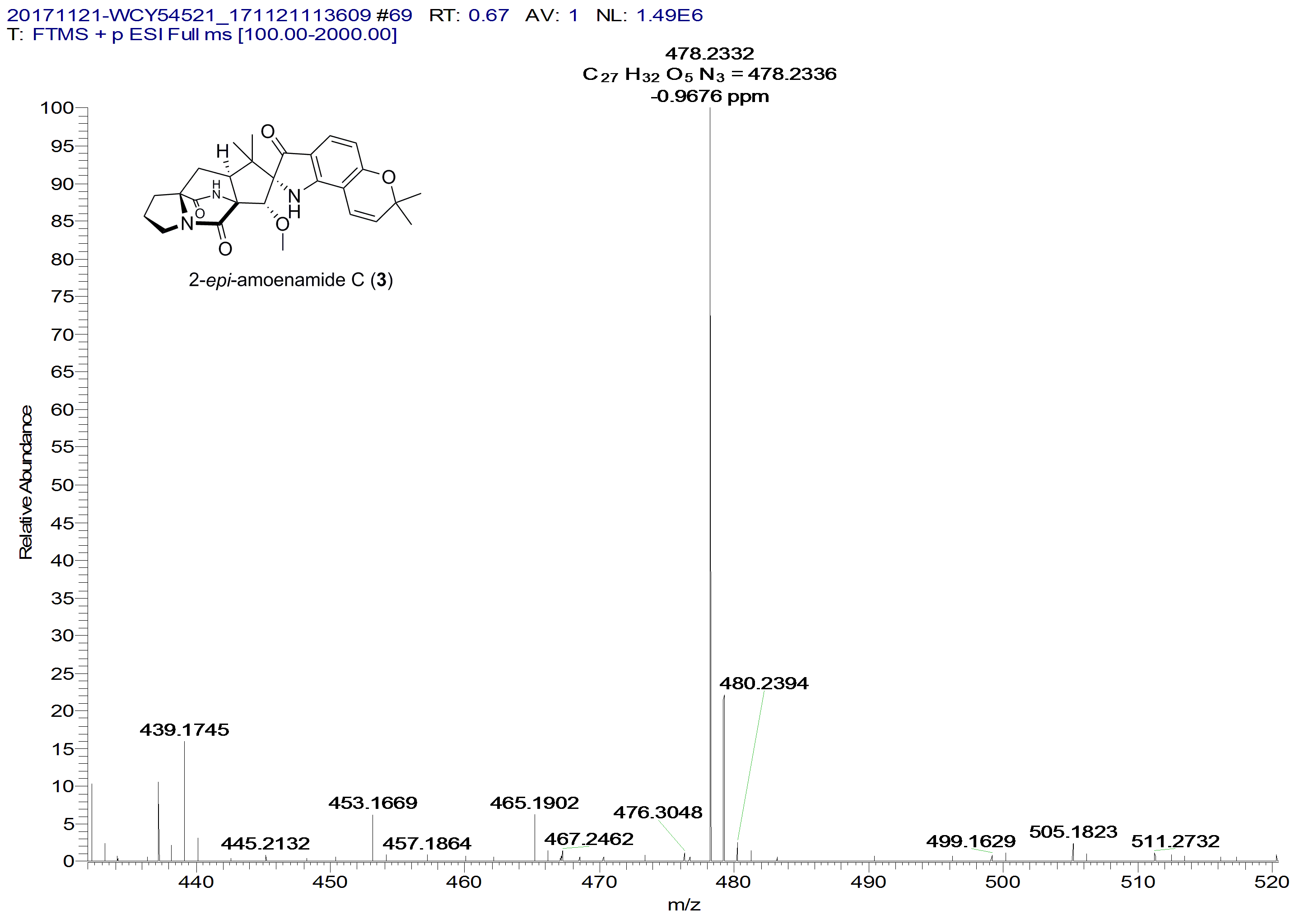
**

**Figure S24.** HRESIMS spectrum of compound **3**


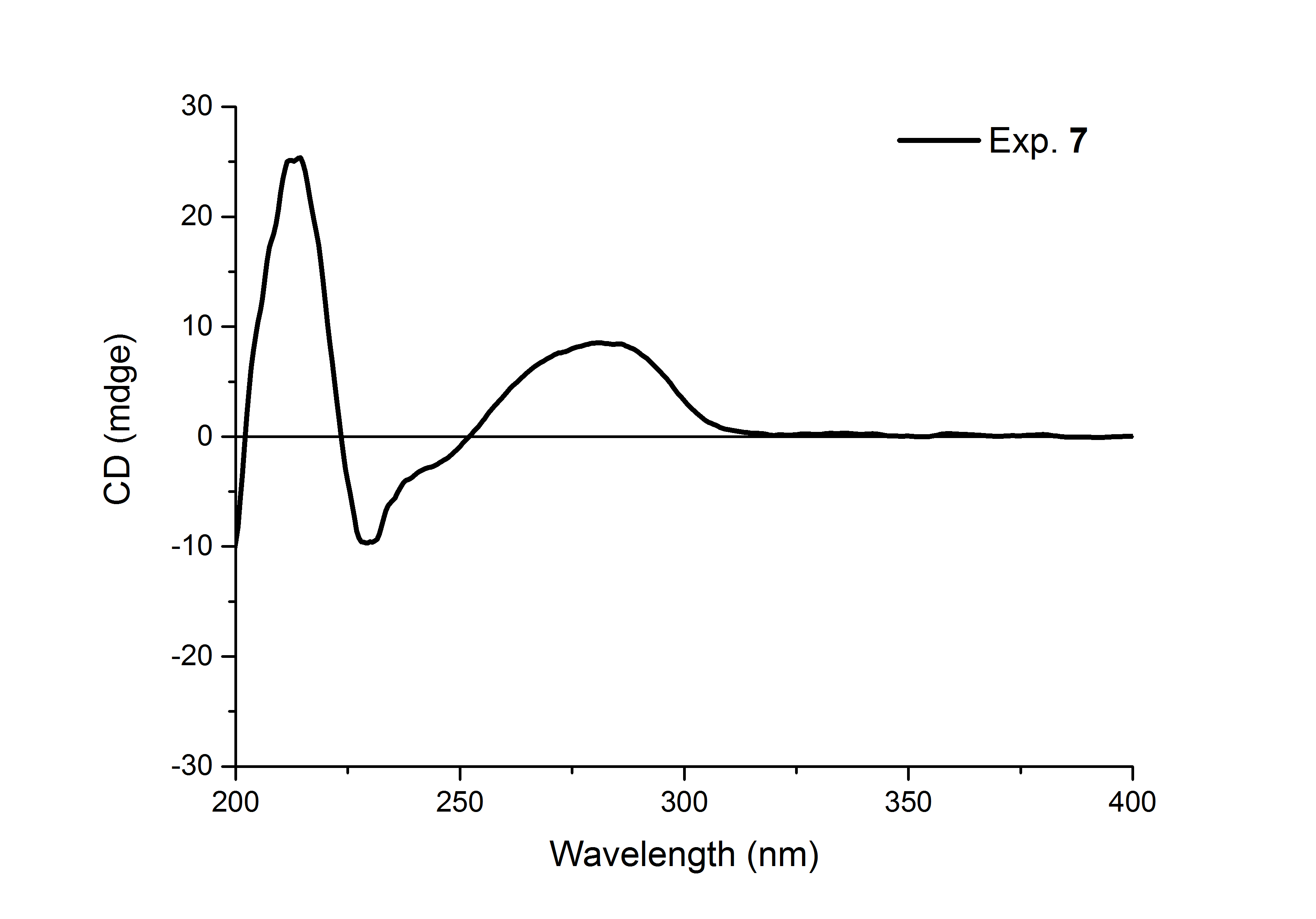


**Figure S25.** ECD spectrum of compound **7**.

**
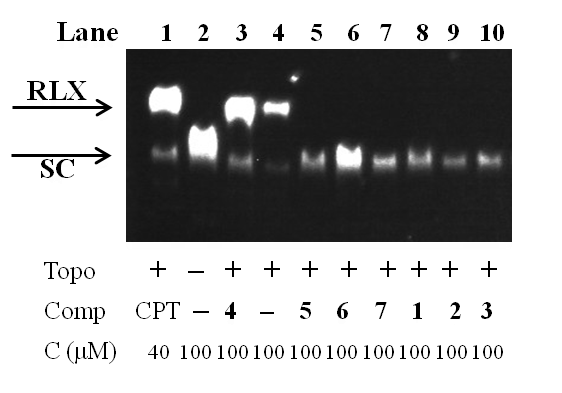
**

**Figure S26.** DNA Topo I inhibitory activity of the isolated compounds.

**Table S1.** Comparison of chemical shift differences between **1** and the reported speramide B

|  | | *δ*_H_, mult | | |  | *δ*_C_, mult | | |  |
| --- | --- | --- | --- | --- | --- | --- | --- | --- | --- |
| Position. | | **1** | speramide B^1^ | |  | **1** | speramide B^1^ | |  |
| 2 | |  | | |  |  | 98.5, C | | 91.3, C |
| 3 | |  | | |  |  | 96.4, C | | 87.4, C |
| 4 | | 6.98, d (8.0) | | | 7.00, d (8.1) |  | 125.4, CH | | 124.0, CH |
| 5 | | 6.04, d (8.0) | | | 6.13, d (8.1) |  | 105.8, CH | | 106.3, CH |
| 5a | |  | | |  |  | 154.7, C | | 153.7, C |
| 9a | |  | | |  |  | 102.8, C | | 104.6, C |
| 9b | |  | | |  |  | 148.4, C | | 145.8, C |
| 3a | |  | | |  |  | 119.5, C | | 122.9, C |
| 12 | | 2.35, t (12.3) ; 2.67, dd (12.6, 7.0) | | | 1.60, dd (7.6, 12.5); 2.49, overlapped |  | 39.3, CH_2_ | | 34.9, CH_2_ |
| 13 | | 4.63, dd (11.8,7.0) | | | 4.01, dd (11.4, 7.8) |  | 63.2, CH | | 57.2, CH |
| 14 | |  | | |  |  | 166.8, C | | 167.1, C |
| 16 | | 3.38, m | | | 3.42, m; 3.31, m |  | 44.9, CH_2_ | | 44.3, CH_2_ |
| 17 | | 1.86, m; 2.06, m | | | 1.95, m; 1.79, m |  | 20.9, CH_2_ | | 20.6, CH_2_ |
| 18 | | 2.06, m | | | 2.00, m; 1.95, m |  | 36.6, CH_2_ | | 36.7, CH_2_ |
| 19 | |  | | |  |  | 89.0, C | | 89.8, C |
| 20 | |  | | |  |  | 166.0, C | | 170.3, C |
| 22 | | 3.41, m | | | 4.82, dd (17.0, 2.4);  4.81, dd (11.0, 2.4) |  | 60.8, CH_2_ | | 110.8, CH_2_ |
| 23 | | 3.57, dd (7.3, 3.0) | | | 6.30, dd (17.0, 11.0) |  | 91.7, CH | | 145.6, CH |
| 24 | |  | | |  |  | 46.9, C | | 44.8, C |
| 25 | | 0.75, s | | | 1.17, s |  | 17.8, CH_3_ | | 24.7, CH_3_ |
| 26 | | 1.30, s | | | 1.21, s |  | 21.8, CH_3_ | | 24.0, CH_3_ |
| 9 | | 6.85, d (9.5) | | | 6.54, d (9.8) |  | 118.6, CH | | 117.5, CH |
| 8 | | 5.55, d (9.5) | | | 5.61, d (9.8) |  | 127.8, CH | | 128.2, CH |
| 7 | |  | | |  |  | 75.8, C | |  |
| 10 | | 1.33, s or 1.34, s | | | 1.33, s |  | 28.3, CH_3_ | | 27.5, CH_3_ |
| 11 | | 1.34, s or 1.33, s | | | 1.36, s |  | 28.1, CH_3_ | | 28.0, CH_3_ |
| 1-NH | | 6.85, s | | | 6.72, s |  |  | |  |
| 3-OH | | 4.59, br s | | | 5.60, s |  |  | |  |
| 19-OH | | 6.74, s | | | 6.51, s |  |  | |  |

**Table S2.** Comparison of chemical shift differences between **2** and the reported taichunamide F

|  | | *δ*_H_, mult | | |  | *δ*_C_, mult | | |  |
| --- | --- | --- | --- | --- | --- | --- | --- | --- | --- |
| Position. | | **2** | taichunamide F^2^ | |  | **2** | | taichunamide F^2^ |  |
| 2 | |  | | |  |  | 151.9, C | 152.8, C | |
| 3 | |  | | |  |  | 77.5, C | 77.8, C | |
| 4 | | 7.36, d (8.0) | | | 7.42, d (8.0) |  | 123.7, CH | 122.7, CH | |
| 5 | | 6.88, d (8.0) | | | 6.82, d (8.0) |  | 116.4, CH | 115.6, CH | |
| 5a | |  | | |  |  | 153.9, C | 153.7, C | |
| 9a | |  | | |  |  | 111.4, C | 111.5, C | |
| 9b | |  | | |  |  | 139.6, C | 140.2, C | |
| 3a | |  | | |  |  | 128.9, C | 128.8, C | |
| 12 | | 4.12, s | | | 4.71, s |  | 75.6, CH | 76.4, CH | |
| 13 | |  | | |  |  | 61.8, C | 61.4, C | |
| 14 | |  | | |  |  | 168.0, C | 168.2, C | |
| 16 | | 3.40, t (6.4) | | | 3.33, m |  | 43.9, CH_2_ | 43.7, CH_2_ | |
| 17 | | 2.03, m; 1.84, m | | | 1.78, m; 1.95, m |  | 24.0, CH_2_ | 23.9, CH_2_ | |
| 18 | | 2.55, m; 1.84, m | | | 2.50, m; 1.79, m |  | 28.7, CH_2_ | 28.2, CH_2_ | |
| 19 | |  | | |  |  | 66.3, C | 66.6, C | |
| 20 | |  | | |  |  | 171.6, C | 171.8, C | |
| 22 | | 2.03, m; 1.84, m | | | 2.09, m; 1.73, m |  | 30.0, CH_2_ | 31.2, CH_2_ | |
| 23 | | 3.53, dd (9.9, 8.0) | | | 3.06, dd (10.3, 6.5) |  | 49.5, CH | 41.4, CH | |
| 24 | |  | | |  |  | 36.1, C | 36.1, C | |
| 25 | | 1.15, s | | | 1.29, s |  | 13.1, CH_3_ | 13.6, CH_3_ | |
| 26 | | 1.30, s | | | 1.30, s |  | 22.4, CH_3_ | 22.0, CH_3_ | |
| 9 | | 7.76, d (10.2) | | | 7.77, d (9.8) |  | 115.4, CH | 115.6, CH | |
| 8 | | 5.93, d (10.2) | | | 5.91, d (9.8) |  | 133.0, CH | 132.8, CH | |
| 7 | |  | | |  |  | 76.2, C | 75.8, C | |
| 10 | | 1.42, s | | | 1.38, s |  | 27.5, CH_3_ | 27.4, CH_3_ | |
| 11 | | 1.40, s | | | 1.40, s |  | 27.4, CH_3_ | 27.4, CH_3_ | |
| 12-OMe | | 3.31, s | | | 3.02, s |  | 59.5, CH_3_ | 61.6, CH_3_ | |
| 1-NH | |  | | | 10.03, brs |  |  |  | |
| 3-OH | | 4.59, br s | | |  |  |  |  | |

**Table S3.** Comparison of partial chemical shift differences between **3** and the reported amoenamide C

|  | | *δ*_H_, mult | | |  | *δ*_C_, mult | |  |
| --- | --- | --- | --- | --- | --- | --- | --- | --- |
| Position. | | **3** | amoenamide C ^2^ | |  | **3** | amoenamide C ^2^ |  |
| 2 | |  | | |  |  | 89.0, C | 83.2, C |
| 3 | |  | | |  |  | 197.1, C | 197.8, C |
| 4 | | 7.28, d (8.3) | | | 7.11, d (8.3) |  | 125.3, CH | 125.3, CH |
| 5 | | 6.32, d (8.3) | | | 6.07, d (8.3) |  | 108.6, CH | 107.8, CH |
| 5a | |  | | |  |  | 161.8, C | 160.0, C |
| 9a | |  | | |  |  | 104.7, C | 103.4, C |
| 9b | |  | | |  |  | 154.9, C | 158.6, C |
| 3a | |  | | |  |  | 113.4, C | 115.0, C |
| 12 | | 6.02, s | | | 4.89, s |  | 80.7, CH | 82.0, CH |
| 13 | |  | | |  |  | 60.4, C | 63.9, C |
| 14 | |  | | |  |  | 167.9, C | 169.1, C |
| 16 | | 3.20, m; 3.33, m | | | 3.33, m |  | 43.4, CH_2_ | 44.0, CH_2_ |
| 17 | | 1.74, m; 1.96, m | | | 1.78, m; 1.95, m |  | 24.1, CH_2_ | 24.8, CH_2_ |
| 18 | | 2.45, m | | | 2.50, m; 1.79, m |  | 28.5, CH_2_ | 30.4, CH_2_ |
| 19 | |  | | |  |  | 66.4, C | 68.9, C |
| 20 | |  | | |  |  | 172.1, C | 173.4, C |
| 22 | | 1.78, m; 1.98, m | | | 2.09, m; 1.73, m |  | 29.4, CH_2_ | 29.5, CH_2_ |
| 23 | | 2.88, dd (10.5, 5.9) | | | 3.63, t (9.9) |  | 41.3, CH | 54.4, CH |
| 24 | |  | | |  |  | 40.2, C | 43.4, C |
| 25 | | 0.50, s | | | 1.29, s |  | 14.7, CH_3_ | 21.4, CH_3_ |
| 26 | | 1.06, s | | | 1.30, s |  | 20.3, CH_3_ | 19.0, CH_3_ |
| 9 | | 7.25, d (10.0) | | | 7.77, d (9.8) |  | 119.5, CH | 116.8, CH |
| 8 | | 5.77, d (10.0) | | | 5.91, d (9.8) |  | 127.6, CH | 127.6, CH |
| 7 | |  | | |  |  | 75.8, C | 77.6, C |
| 10 | | 1.46, s | | | 1.38, s |  | 27.1, CH_3_ | 28.5, CH_3_ |
| 11 | | 1.34, s | | | 1.40, s |  | 25.7, CH_3_ | 28.4, CH_3_ |
| 12-OMe | | 3.29, s | | | 3.10, s |  | 54.4, CH_3_ | 59.9, CH_3_ |
| 1-NH | | 6.24, s | | | 7.69, s |  |  |  |
| 19-NH | | 8.08, s | | |  |  |  |  |

1. Y. W. Chang, C. M. Yuan, J. Zhang, S. Liu, P. Cao, H. M. Hua, Y. T. Di and X. J. Hao, Tetrahedron Lett., 2016, 57, 4952−4955.
2. Kagiyama, I.; Kato, H.; Nehira, T.; Frisvad, J. C.; Sherman, D. H.; Williams, R. M.; Tsukamoto, S. *Angew. Chem. Int. Ed.* **2016**, *55*, 1128−1132.
3. Zhang, P.; Yuan, X. L.; Du, Y. M.; Zhang, H. B.; Shen, G. M.; Zhang, Z. F.; Liang, Y. J.; Zhao, D. L.; Xu, K. *J. Agric. Food Chem.* **2019**, *67*, 11994−12001.

**ECD calculation details of compound 1**

1. Methods

Monte Carlo conformational searches were carried out by means of the Spartan’s 14 software using Merck Molecular Force Field (MMFF). The conformers with Boltzmann-population of over 5% were chosen for ECD calculations, and then the conformers were initially optimized at B3LYP/6-31g level in gas. The theoretical calculation of ECD was conducted in MeOH using Time-dependent Density functional theory (TD-DFT) at the B3LYP/6-31+g (d, p) level for all conformers of compounds **1**. Rotatory strengths for a total of 30 excited states were calculated. ECD spectra were generated using the program SpecDis 1.6 (University of Würzburg, Würzburg, Germany) and GraphPad Prism 5 (University of California San Diego, USA) from dipole-length rotational strengths by applying Gaussian band shapes with sigma = 0.3 eV.

2. Results

Table S4.1.Gibbs free energies*^a^* and equilibrium populations*^b^* of low-energy conformers of **1A**.

| Conformers | In gas | |
| --- | --- | --- |
|  | *G^a^* | *P* (%)^b^ |
| **1a** | -1019431.92333567 | 67.87 |
| **1b** | -1019431.27825539 | 22.82 |
| **1c** | -1019430.74738193 | 9.31 |
|  |  |  |
|  |  |  |
|  |  |  |
| *^a^*B3LYP/6-31G(d,p), in kcal/mol. *^b^*From *G* values at 298.15K. | | |

Table S4.2**.**Cartesian coordinates for the low-energy reoptimized MMFF conformers of **1A** at B3LYP/6-31G(d,p) level of theory in gas.

| **1a** | | Standard Orientation  (Ångstroms) | | | |
| --- | --- | --- | --- | --- | --- |
| Center number | Atomic number | Atomic Type | X | Y | Z |
| 1. | 6. | 0. | -2.054737 | 0.590218 | 1.147890 |
| 2. | 7. | 0. | -1.523153 | 0.197707 | -0.179759 |
| 3. | 6. | 0. | -1.208853 | 1.122391 | -1.117774 |
| 4. | 6. | 0. | -1.540909 | 2.574989 | -0.727700 |
| 5. | 7. | 0. | -1.244349 | 2.826804 | 0.664229 |
| 6. | 6. | 0. | -1.444073 | 1.907157 | 1.646885 |
| 7. | 6. | 0. | -0.691801 | 3.631320 | -1.450298 |
| 8. | 6. | 0. | -0.734728 | 4.820303 | -0.474818 |
| 9. | 6. | 0. | -0.665831 | 4.154745 | 0.910397 |
| 10. | 6. | 0. | -1.752832 | -0.597415 | 2.066916 |
| 11. | 6. | 0. | -0.837280 | -1.539220 | 1.271592 |
| 12. | 6. | 0. | -1.155548 | -1.256711 | -0.269474 |
| 13. | 6. | 0. | 0.644115 | -1.228471 | 1.311053 |
| 14. | 6. | 0. | 1.146705 | -1.188713 | 0.004984 |
| 15. | 7. | 0. | 0.135451 | -1.414390 | -0.936459 |
| 16. | 6. | 0. | 1.500937 | -1.092029 | 2.399074 |
| 17. | 6. | 0. | 2.863833 | -0.877702 | 2.178888 |
| 18. | 6. | 0. | 3.351711 | -0.816949 | 0.869281 |
| 19. | 6. | 0. | 2.509299 | -0.984262 | -0.252993 |
| 20. | 8. | 0. | 4.694944 | -0.654773 | 0.707696 |
| 21. | 6. | 0. | 5.205097 | -0.113968 | -0.549579 |
| 22. | 6. | 0. | 4.405874 | -0.643160 | -1.721538 |
| 23. | 6. | 0. | 3.126203 | -1.011003 | -1.571606 |
| 24. | 8. | 0. | -1.193602 | 2.122494 | 2.825143 |
| 25. | 8. | 0. | -0.722975 | 0.882996 | -2.229868 |
| 26. | 1. | 0. | -3.134628 | 0.751817 | 1.060941 |
| 27. | 6. | 0. | -2.283600 | -2.119251 | -0.968345 |
| 28. | 6. | 0. | -3.489591 | -2.273920 | -0.034913 |
| 29. | 8. | 0. | -4.359493 | -1.144489 | 0.177735 |
| 30. | 6. | 0. | -1.764950 | -3.547027 | -1.256039 |
| 31. | 6. | 0. | -2.706549 | -1.471675 | -2.301827 |
| 32. | 8. | 0. | -1.088204 | -2.869264 | 1.726757 |
| 33. | 6. | 0. | -4.891345 | -2.301098 | -0.482405 |
| 34. | 8. | 0. | -2.947390 | 2.790024 | -0.923342 |
| 35. | 6. | 0. | 5.119801 | 1.419694 | -0.477533 |
| 36. | 6. | 0. | 6.659327 | -0.586006 | -0.603624 |
| 37. | 1. | 0. | -1.085281 | 3.856145 | -2.443684 |
| 38. | 1. | 0. | 0.326644 | 3.251467 | -1.573847 |
| 39. | 1. | 0. | -1.683564 | 5.353056 | -0.585019 |
| 40. | 1. | 0. | 0.077813 | 5.532759 | -0.634510 |
| 41. | 1. | 0. | -1.234096 | 4.682268 | 1.680726 |
| 42. | 1. | 0. | 0.363065 | 4.045589 | 1.273110 |
| 43. | 1. | 0. | -2.670514 | -1.125638 | 2.327627 |
| 44. | 1. | 0. | -1.285276 | -0.251799 | 2.990299 |
| 45. | 1. | 0. | 0.195907 | -0.874364 | -1.795682 |
| 46. | 1. | 0. | 1.114828 | -1.128088 | 3.413796 |
| 47. | 1. | 0. | 3.558702 | -0.749574 | 3.000876 |
| 48. | 1. | 0. | 4.900179 | -0.681050 | -2.688121 |
| 49. | 1. | 0. | 2.545346 | -1.370880 | -2.416323 |
| 50. | 1. | 0. | -3.264499 | -2.823707 | 0.876687 |
| 51. | 1. | 0. | -0.919593 | -3.520018 | -1.945447 |
| 52. | 1. | 0. | -1.449751 | -4.053333 | -0.342637 |
| 53. | 1. | 0. | -2.566046 | -4.138929 | -1.711229 |
| 54. | 1. | 0. | -3.280750 | -0.557885 | -2.131159 |
| 55. | 1. | 0. | -3.328963 | -2.168387 | -2.870958 |
| 56. | 1. | 0. | -1.843373 | -1.218002 | -2.917506 |
| 57. | 1. | 0. | -0.285294 | -3.382735 | 1.563022 |
| 58. | 1. | 0. | -5.619019 | -2.871965 | 0.094418 |
| 59. | 1. | 0. | -5.145090 | -2.158524 | -1.530086 |
| 60. | 1. | 0. | -3.145844 | 2.586655 | -1.849603 |
| 61. | 1. | 0. | 5.521577 | 1.869277 | -1.391338 |
| 62. | 1. | 0. | 4.079376 | 1.738699 | -0.370926 |
| 63. | 1. | 0. | 5.692319 | 1.792202 | 0.377862 |
| 64. | 1. | 0. | 6.705357 | -1.677672 | -0.633493 |
| 65. | 1. | 0. | 7.202169 | -0.237944 | 0.279447 |
| 66. | 1. | 0. | 7.154765 | -0.189594 | -1.495232 |
|  |  |  |  |  |  |
|  |  |  |  |  |  |
|  |  |  |  |  |  |

| **1b** | | Standard Orientation  (Ångstroms) | | | |
| --- | --- | --- | --- | --- | --- |
| Center number | Atom number | Type | X | Y | Z |
| 1. | 6. | 0. | -2.239319 | 0.612156 | 1.228209 |
| 2. | 7. | 0. | -1.757270 | 0.195920 | -0.110147 |
| 3. | 6. | 0. | -1.608166 | 1.100793 | -1.111293 |
| 4. | 6. | 0. | -2.124724 | 2.514334 | -0.781848 |
| 5. | 7. | 0. | -1.764161 | 2.898203 | 0.562614 |
| 6. | 6. | 0. | -1.768618 | 2.026950 | 1.607608 |
| 7. | 6. | 0. | -1.502318 | 3.630055 | -1.632494 |
| 8. | 6. | 0. | -1.638869 | 4.863396 | -0.722511 |
| 9. | 6. | 0. | -1.363774 | 4.307266 | 0.684897 |
| 10. | 6. | 0. | -1.749506 | -0.467863 | 2.194090 |
| 11. | 6. | 0. | -0.817547 | -1.405322 | 1.402935 |
| 12. | 6. | 0. | -1.191383 | -1.197537 | -0.149128 |
| 13. | 6. | 0. | 0.645896 | -1.001767 | 1.390233 |
| 14. | 6. | 0. | 1.107427 | -0.905978 | 0.076151 |
| 15. | 7. | 0. | 0.093542 | -1.211011 | -0.842501 |
| 16. | 6. | 0. | 1.521190 | -0.816596 | 2.454381 |
| 17. | 6. | 0. | 2.856152 | -0.490271 | 2.201923 |
| 18. | 6. | 0. | 3.300122 | -0.371051 | 0.881767 |
| 19. | 6. | 0. | 2.441234 | -0.590031 | -0.218519 |
| 20. | 8. | 0. | 4.623459 | -0.102802 | 0.684124 |
| 21. | 6. | 0. | 5.046655 | 0.498560 | -0.575688 |
| 22. | 6. | 0. | 4.262338 | -0.081969 | -1.734029 |
| 23. | 6. | 0. | 3.021108 | -0.553944 | -1.553319 |
| 24. | 8. | 0. | -1.472476 | 2.347068 | 2.749637 |
| 25. | 8. | 0. | -1.138300 | 0.863545 | -2.228863 |
| 26. | 1. | 0. | -3.335736 | 0.664434 | 1.213251 |
| 27. | 6. | 0. | -2.194452 | -2.232710 | -0.797555 |
| 28. | 6. | 0. | -1.412228 | -3.497648 | -1.198480 |
| 29. | 8. | 0. | -0.688931 | -4.275241 | -0.225593 |
| 30. | 6. | 0. | -2.771072 | -1.703623 | -2.133452 |
| 31. | 6. | 0. | -3.391783 | -2.522700 | 0.129742 |
| 32. | 8. | 0. | -1.010631 | -2.695826 | 1.945090 |
| 33. | 6. | 0. | -1.823501 | -4.874547 | -0.875279 |
| 34. | 8. | 0. | -3.560898 | 2.503928 | -0.868325 |
| 35. | 6. | 0. | 4.830963 | 2.018220 | -0.475965 |
| 36. | 6. | 0. | 6.534271 | 0.156340 | -0.679063 |
| 37. | 1. | 0. | -2.001996 | 3.732133 | -2.598234 |
| 38. | 1. | 0. | -0.453281 | 3.388836 | -1.826440 |
| 39. | 1. | 0. | -2.660109 | 5.251365 | -0.776421 |
| 40. | 1. | 0. | -0.952670 | 5.669317 | -0.992177 |
| 41. | 1. | 0. | -1.938561 | 4.799785 | 1.473280 |
| 42. | 1. | 0. | -0.304253 | 4.365979 | 0.959904 |
| 43. | 1. | 0. | -2.581842 | -1.058709 | 2.578729 |
| 44. | 1. | 0. | -1.243408 | -0.005560 | 3.042972 |
| 45. | 1. | 0. | 0.080289 | -0.678334 | -1.707338 |
| 46. | 1. | 0. | 1.169650 | -0.910029 | 3.477893 |
| 47. | 1. | 0. | 3.562897 | -0.324946 | 3.007138 |
| 48. | 1. | 0. | 4.732486 | -0.072381 | -2.713340 |
| 49. | 1. | 0. | 2.449311 | -0.954229 | -2.386260 |
| 50. | 1. | 0. | -0.831710 | -3.362626 | -2.109097 |
| 51. | 1. | 0. | -3.426739 | -0.847270 | -1.958793 |
| 52. | 1. | 0. | -1.996629 | -1.394201 | -2.836984 |
| 53. | 1. | 0. | -3.371787 | -2.490945 | -2.601655 |
| 54. | 1. | 0. | -3.088137 | -3.014172 | 1.053594 |
| 55. | 1. | 0. | -4.124241 | -3.147130 | -0.391047 |
| 56. | 1. | 0. | -3.900920 | -1.589228 | 0.388563 |
| 57. | 1. | 0. | -0.614669 | -3.357220 | 1.347511 |
| 58. | 1. | 0. | -1.576521 | -5.685153 | -1.559513 |
| 59. | 1. | 0. | -2.681997 | -5.054685 | -0.233047 |
| 60. | 1. | 0. | -3.792812 | 2.279374 | -1.781816 |
| 61. | 1. | 0. | 5.163916 | 2.513889 | -1.393713 |
| 62. | 1. | 0. | 3.770484 | 2.242767 | -0.332513 |
| 63. | 1. | 0. | 5.394743 | 2.427541 | 0.368439 |
| 64. | 1. | 0. | 6.672940 | -0.926736 | -0.728283 |
| 65. | 1. | 0. | 7.070451 | 0.537115 | 0.194562 |
| 66. | 1. | 0. | 6.967714 | 0.606787 | -1.577362 |
|  |  |  |  |  |  |
|  |  |  |  |  |  |
|  |  |  |  |  |  |

| **1c** | | Standard Orientation  (Ångstroms) | | | |
| --- | --- | --- | --- | --- | --- |
| Center number | Atom number | Type | X | Y | Z |
| 1. | 6. | 0. | -2.158133 | 0.926474 | 1.225061 |
| 2. | 7. | 0. | -1.701172 | 0.320815 | -0.048567 |
| 3. | 6. | 0. | -1.432148 | 1.088755 | -1.134785 |
| 4. | 6. | 0. | -1.777184 | 2.580740 | -0.961238 |
| 5. | 7. | 0. | -1.387328 | 3.053123 | 0.347910 |
| 6. | 6. | 0. | -1.517673 | 2.303006 | 1.474716 |
| 7. | 6. | 0. | -1.012377 | 3.520986 | -1.903097 |
| 8. | 6. | 0. | -1.004330 | 4.847405 | -1.123227 |
| 9. | 6. | 0. | -0.815986 | 4.407413 | 0.338668 |
| 10. | 6. | 0. | -1.815612 | -0.100413 | 2.306418 |
| 11. | 6. | 0. | -0.926772 | -1.155330 | 1.634221 |
| 12. | 6. | 0. | -1.308868 | -1.127868 | 0.081050 |
| 13. | 6. | 0. | 0.551492 | -0.835720 | 1.564750 |
| 14. | 6. | 0. | 1.003785 | -1.006115 | 0.250740 |
| 15. | 7. | 0. | -0.041277 | -1.385476 | -0.598037 |
| 16. | 6. | 0. | 1.447199 | -0.523087 | 2.582954 |
| 17. | 6. | 0. | 2.799293 | -0.346135 | 2.279238 |
| 18. | 6. | 0. | 3.237368 | -0.499803 | 0.959667 |
| 19. | 6. | 0. | 2.354563 | -0.846758 | -0.088247 |
| 20. | 8. | 0. | 4.572534 | -0.367708 | 0.724636 |
| 21. | 6. | 0. | 5.037455 | -0.046675 | -0.622355 |
| 22. | 6. | 0. | 4.195303 | -0.755316 | -1.661738 |
| 23. | 6. | 0. | 2.922963 | -1.088802 | -1.406950 |
| 24. | 8. | 0. | -1.196178 | 2.697162 | 2.586955 |
| 25. | 8. | 0. | -0.979060 | 0.681359 | -2.208693 |
| 26. | 1. | 0. | -3.239463 | 1.105790 | 1.173058 |
| 27. | 6. | 0. | -2.445371 | -2.086925 | -0.435079 |
| 28. | 6. | 0. | -1.924900 | -3.533660 | -0.421898 |
| 29. | 8. | 0. | -2.735941 | -4.508554 | -1.103299 |
| 30. | 6. | 0. | -2.899407 | -1.696325 | -1.857404 |
| 31. | 6. | 0. | -3.694314 | -2.055140 | 0.475041 |
| 32. | 8. | 0. | -1.151009 | -2.393176 | 2.308726 |
| 33. | 6. | 0. | -1.408442 | -4.254751 | -1.588857 |
| 34. | 8. | 0. | -3.201469 | 2.736487 | -1.078154 |
| 35. | 6. | 0. | 4.957705 | 1.478324 | -0.801490 |
| 36. | 6. | 0. | 6.487656 | -0.533667 | -0.649191 |
| 37. | 1. | 0. | -1.486557 | 3.585429 | -2.884752 |
| 38. | 1. | 0. | 0.000652 | 3.135398 | -2.049225 |
| 39. | 1. | 0. | -1.968513 | 5.350599 | -1.239451 |
| 40. | 1. | 0. | -0.220143 | 5.531580 | -1.455068 |
| 41. | 1. | 0. | -1.333567 | 5.044020 | 1.060674 |
| 42. | 1. | 0. | 0.239584 | 4.362843 | 0.631153 |
| 43. | 1. | 0. | -2.713471 | -0.588064 | 2.687500 |
| 44. | 1. | 0. | -1.314566 | 0.389202 | 3.143190 |
| 45. | 1. | 0. | -0.017479 | -1.012238 | -1.542408 |
| 46. | 1. | 0. | 1.099436 | -0.394691 | 3.604077 |
| 47. | 1. | 0. | 3.523644 | -0.086085 | 3.042453 |
| 48. | 1. | 0. | 4.653266 | -0.952017 | -2.626924 |
| 49. | 1. | 0. | 2.312498 | -1.578860 | -2.160426 |
| 50. | 1. | 0. | -1.595650 | -3.869098 | 0.558300 |
| 51. | 1. | 0. | -3.467713 | -0.763228 | -1.834319 |
| 52. | 1. | 0. | -2.069414 | -1.555804 | -2.550793 |
| 53. | 1. | 0. | -3.552450 | -2.483241 | -2.242488 |
| 54. | 1. | 0. | -3.472344 | -2.411078 | 1.482416 |
| 55. | 1. | 0. | -4.455733 | -2.712085 | 0.045304 |
| 56. | 1. | 0. | -4.123195 | -1.049880 | 0.534443 |
| 57. | 1. | 0. | -0.333566 | -2.905672 | 2.243417 |
| 58. | 1. | 0. | -0.693892 | -5.065070 | -1.443391 |
| 59. | 1. | 0. | -1.354061 | -3.763762 | -2.558296 |
| 60. | 1. | 0. | -3.454541 | 2.430462 | -1.961956 |
| 61. | 1. | 0. | 5.326471 | 1.767365 | -1.790891 |
| 62. | 1. | 0. | 3.922300 | 1.817954 | -0.711262 |
| 63. | 1. | 0. | 5.562076 | 1.982370 | -0.040692 |
| 64. | 1. | 0. | 6.529461 | -1.615764 | -0.500811 |
| 65. | 1. | 0. | 7.062684 | -0.050286 | 0.145360 |
| 66. | 1. | 0. | 6.952069 | -0.293573 | -1.610513 |
|  |  |  |  |  |  |
|  |  |  |  |  |  |
|  |  |  |  |  |  |

**ECD calculation details of compound 2**

1. Methods

Monte Carlo conformational searches were carried out by means of the Spartan’s 10 software using Merck Molecular Force Field (MMFF). The conformers with Boltzmann-population of over 5% were chosen for ECD calculations, and then the conformers were initially optimized at B3LYP/6-31+g (d, p) level in MeOH using the CPCM polarizable conductor calculation model. The theoretical calculation of ECD was conducted in MeOH using Time-dependent Density functional theory (TD-DFT) at the B3LYP/6-311+g (d, p) level for all conformers of compound **2**. Rotatory strengths for a total of 50 excited states were calculated. ECD spectra were generated using the program SpecDis 1.6 (University of Würzburg, Würzburg, Germany) and GraphPad Prism 5 (University of California San Diego, USA) from dipole-length rotational strengths by applying Gauwcyian band shapes with sigma = 0.3 eV.

2. Results

| Table S5.1.Gibbs free energies*^a^* and equilibrium populations*^b^* of low-energy conformers of **2**.   \| Conformers \| In MeOH \| \| \| --- \| --- \| --- \| \| ∆*G* \| *P* (%) \| \| **2a** \| 0 \| 44.72 \| \| **2b** \| 1.25 \| 16.41 \| \| **2c** \| 0.34 \| 27.96 \| \| **2d** \| 1.33 \| 10.92 \| \| *^a^*B3LYP/6-31+G(d,p), in kcal/mol. *^b^*From ∆*G* values at 298.15K. \| \| \| \|  \| \| \| |
| --- | --- | --- | --- | --- | --- | --- | --- | --- | --- | --- | --- | --- | --- | --- | --- | --- | --- | --- | --- | --- | --- | --- | --- |

Table S5.2**.**Cartesian coordinates for the low-energy reoptimized MMFF conformers of **2** at B3LYP/6-311+G(d,p) level of theory in CH_3_OH.

| **2a** | | Standard Orientation  (Ångstroms) | | | |
| --- | --- | --- | --- | --- | --- |
| Center number | Atomic number | Atomic Type | X | Y | Z |
| 1. | 6. | 0. | -2.370732 | 2.054989 | -0.767815 |
| 2. | 6. | 0. | -3.748663 | 2.049933 | -0.513283 |
| 3. | 6. | 0. | -4.414573 | 0.855081 | -0.227594 |
| 4. | 6. | 0. | -3.744811 | -0.397794 | -0.204981 |
| 5. | 8. | 0. | -5.764555 | 0.919287 | -0.048017 |
| 6. | 6. | 0. | -6.435965 | -0.123379 | 0.739506 |
| 7. | 6. | 0. | -5.821666 | -1.470284 | 0.435569 |
| 8. | 6. | 0. | -4.550075 | -1.595571 | 0.023034 |
| 9. | 6. | 0. | -7.898760 | -0.058371 | 0.298789 |
| 10. | 6. | 0. | -6.285441 | 0.224551 | 2.228978 |
| 11. | 6. | 0. | 2.235115 | -0.812012 | -0.927762 |
| 12. | 6. | 0. | 2.184335 | 0.519349 | -0.078672 |
| 13. | 6. | 0. | 3.671138 | -1.393490 | -0.821505 |
| 14. | 6. | 0. | 4.574463 | -0.393854 | -0.055229 |
| 15. | 7. | 0. | 3.975983 | -0.206893 | 1.290187 |
| 16. | 6. | 0. | 2.720175 | 0.260302 | 1.362949 |
| 17. | 8. | 0. | 2.087170 | 0.478526 | 2.403477 |
| 18. | 6. | 0. | 6.004548 | -0.851588 | 0.247102 |
| 19. | 6. | 0. | 6.286790 | -0.329103 | 1.672087 |
| 20. | 6. | 0. | 4.928013 | -0.425592 | 2.386562 |
| 21. | 6. | 0. | 4.461395 | 0.969064 | -0.778106 |
| 22. | 7. | 0. | 3.181407 | 1.402490 | -0.706244 |
| 23. | 8. | 0. | 5.384257 | 1.560087 | -1.343684 |
| 24. | 6. | 0. | 0.779030 | 1.202119 | -0.043158 |
| 25. | 6. | 0. | -0.227547 | 0.508372 | -0.984922 |
| 26. | 6. | 0. | -0.199939 | -0.986578 | -0.735636 |
| 27. | 6. | 0. | 1.065024 | -1.813190 | -0.672021 |
| 28. | 6. | 0. | -1.683144 | 0.849589 | -0.718548 |
| 29. | 6. | 0. | -2.372588 | -0.329633 | -0.452889 |
| 30. | 7. | 0. | -1.415545 | -1.427734 | -0.474115 |
| 31. | 6. | 0. | 1.150217 | -2.535819 | 0.697654 |
| 32. | 6. | 0. | 1.063620 | -2.872122 | -1.805746 |
| 33. | 8. | 0. | -1.772239 | -2.638754 | -0.225497 |
| 34. | 8. | 0. | 0.126425 | 0.768981 | -2.355892 |
| 35. | 8. | 0. | 0.833364 | 2.567556 | -0.442067 |
| 36. | 6. | 0. | 0.950420 | 3.519167 | 0.626522 |
| 37. | 1. | 0. | 2.096918 | -0.456015 | -1.950222 |
| 38. | 1. | 0. | -1.859671 | 2.986158 | -0.986264 |
| 39. | 1. | 0. | -4.320153 | 2.971961 | -0.530776 |
| 40. | 1. | 0. | -6.442069 | -2.345674 | 0.607428 |
| 41. | 1. | 0. | -4.097123 | -2.562033 | -0.152263 |
| 42. | 1. | 0. | -8.491869 | -0.779929 | 0.868303 |
| 43. | 1. | 0. | -8.303258 | 0.941883 | 0.479337 |
| 44. | 1. | 0. | -7.993976 | -0.291630 | -0.765554 |
| 45. | 1. | 0. | -6.786154 | -0.530681 | 2.843066 |
| 46. | 1. | 0. | -6.737104 | 1.199908 | 2.437642 |
| 47. | 1. | 0. | -5.230050 | 0.252241 | 2.516088 |
| 48. | 1. | 0. | 4.088824 | -1.585411 | -1.813885 |
| 49. | 1. | 0. | 3.694314 | -2.335526 | -0.268885 |
| 50. | 1. | 0. | 6.047754 | -1.945288 | 0.223536 |
| 51. | 1. | 0. | 6.705993 | -0.463736 | -0.493772 |
| 52. | 1. | 0. | 7.061286 | -0.905344 | 2.183299 |
| 53. | 1. | 0. | 6.611630 | 0.715924 | 1.633768 |
| 54. | 1. | 0. | 4.782033 | 0.328146 | 3.163700 |
| 55. | 1. | 0. | 4.769481 | -1.415999 | 2.830022 |
| 56. | 1. | 0. | 2.875971 | 2.268108 | -1.136383 |
| 57. | 1. | 0. | 0.407590 | 1.123250 | 0.983713 |
| 58. | 1. | 0. | 0.286683 | -3.191269 | 0.820150 |
| 59. | 1. | 0. | 2.052071 | -3.151818 | 0.749104 |
| 60. | 1. | 0. | 1.165268 | -1.833551 | 1.534575 |
| 61. | 1. | 0. | 0.209962 | -3.544528 | -1.694134 |
| 62. | 1. | 0. | 1.979320 | -3.470659 | -1.763034 |
| 63. | 1. | 0. | 1.008897 | -2.395997 | -2.789633 |
| 64. | 1. | 0. | 0.214504 | 1.732259 | -2.443818 |
| 65. | 1. | 0. | 0.111873 | 3.420760 | 1.324787 |
| 66. | 1. | 0. | 0.926453 | 4.505304 | 0.160821 |
| 67. | 1. | 0. | 1.894049 | 3.393875 | 1.168632 |
|  |  |  |  |  |  |
|  |  |  |  |  |  |

| **2b** | | Standard Orientation  (Ångstroms) | | | |
| --- | --- | --- | --- | --- | --- |
| Center number | Atomic number | Atomic Type | X | Y | Z |
| 1. | 6. | 0. | -2.369132 | 2.001423 | -0.922296 |
| 2. | 6. | 0. | -3.748117 | 2.013360 | -0.676536 |
| 3. | 6. | 0. | -4.413894 | 0.841494 | -0.305723 |
| 4. | 6. | 0. | -3.744430 | -0.406792 | -0.189491 |
| 5. | 8. | 0. | -5.764064 | 0.918224 | -0.136705 |
| 6. | 6. | 0. | -6.441053 | -0.063834 | 0.721357 |
| 7. | 6. | 0. | -5.822458 | -1.428623 | 0.526835 |
| 8. | 6. | 0. | -4.550063 | -1.583955 | 0.127717 |
| 9. | 6. | 0. | -7.899830 | -0.036137 | 0.263514 |
| 10. | 6. | 0. | -6.304969 | 0.397261 | 2.181212 |
| 11. | 6. | 0. | 2.229286 | -0.858542 | -0.881836 |
| 12. | 6. | 0. | 2.175394 | 0.506229 | -0.088359 |
| 13. | 6. | 0. | 3.672470 | -1.418977 | -0.775441 |
| 14. | 6. | 0. | 4.579490 | -0.364034 | -0.090852 |
| 15. | 7. | 0. | 4.017106 | -0.123452 | 1.261407 |
| 16. | 6. | 0. | 2.751847 | 0.314576 | 1.348433 |
| 17. | 8. | 0. | 2.138001 | 0.551969 | 2.396341 |
| 18. | 6. | 0. | 6.026102 | -0.782610 | 0.190062 |
| 19. | 6. | 0. | 6.338102 | -0.194922 | 1.583242 |
| 20. | 6. | 0. | 5.001206 | -0.283740 | 2.338727 |
| 21. | 6. | 0. | 4.421084 | 0.965073 | -0.869612 |
| 22. | 7. | 0. | 3.136264 | 1.378916 | -0.781107 |
| 23. | 8. | 0. | 5.319885 | 1.548546 | -1.480668 |
| 24. | 6. | 0. | 0.758037 | 1.161098 | -0.045952 |
| 25. | 6. | 0. | -0.224301 | 0.456105 | -1.002000 |
| 26. | 6. | 0. | -0.200521 | -1.032750 | -0.674197 |
| 27. | 6. | 0. | 1.066686 | -1.853489 | -0.572745 |
| 28. | 6. | 0. | -1.682635 | 0.802316 | -0.782598 |
| 29. | 6. | 0. | -2.371962 | -0.358216 | -0.439518 |
| 30. | 7. | 0. | -1.416641 | -1.458208 | -0.393133 |
| 31. | 6. | 0. | 1.159693 | -2.507288 | 0.830139 |
| 32. | 6. | 0. | 1.067075 | -2.968114 | -1.651384 |
| 33. | 8. | 0. | -1.779439 | -2.655846 | -0.087115 |
| 34. | 8. | 0. | 0.206400 | 0.743866 | -2.342013 |
| 35. | 8. | 0. | 0.790226 | 2.544359 | -0.355704 |
| 36. | 6. | 0. | 0.922762 | 3.418026 | 0.773293 |
| 37. | 1. | 0. | 2.076291 | -0.544072 | -1.915225 |
| 38. | 1. | 0. | -1.850513 | 2.910731 | -1.203987 |
| 39. | 1. | 0. | -4.321240 | 2.930271 | -0.765150 |
| 40. | 1. | 0. | -6.441505 | -2.288928 | 0.766369 |
| 41. | 1. | 0. | -4.095099 | -2.560395 | 0.029876 |
| 42. | 1. | 0. | -8.497494 | -0.711286 | 0.883041 |
| 43. | 1. | 0. | -8.307254 | 0.974504 | 0.360321 |
| 44. | 1. | 0. | -7.984916 | -0.353088 | -0.779852 |
| 45. | 1. | 0. | -6.809731 | -0.310537 | 2.846356 |
| 46. | 1. | 0. | -6.760551 | 1.384360 | 2.310993 |
| 47. | 1. | 0. | -5.252346 | 0.450033 | 2.474977 |
| 48. | 1. | 0. | 4.068944 | -1.665516 | -1.764524 |
| 49. | 1. | 0. | 3.721221 | -2.325235 | -0.166836 |
| 50. | 1. | 0. | 6.089082 | -1.875381 | 0.212500 |
| 51. | 1. | 0. | 6.699504 | -0.414863 | -0.586183 |
| 52. | 1. | 0. | 7.136417 | -0.735934 | 2.096361 |
| 53. | 1. | 0. | 6.642997 | 0.852819 | 1.491921 |
| 54. | 1. | 0. | 4.863997 | 0.497897 | 3.089492 |
| 55. | 1. | 0. | 4.871431 | -1.258079 | 2.825240 |
| 56. | 1. | 0. | 2.805299 | 2.235257 | -1.211419 |
| 57. | 1. | 0. | 0.378534 | 1.014992 | 0.971499 |
| 58. | 1. | 0. | 0.303328 | -3.165632 | 0.984731 |
| 59. | 1. | 0. | 2.068896 | -3.109721 | 0.909884 |
| 60. | 1. | 0. | 1.167672 | -1.765741 | 1.632634 |
| 61. | 1. | 0. | 0.216089 | -3.636821 | -1.503328 |
| 62. | 1. | 0. | 1.985236 | -3.560092 | -1.579971 |
| 63. | 1. | 0. | 1.009039 | -2.543718 | -2.658548 |
| 64. | 1. | 0. | -0.459749 | 0.407657 | -2.959542 |
| 65. | 1. | 0. | 0.867013 | 4.434131 | 0.379482 |
| 66. | 1. | 0. | 1.882436 | 3.276927 | 1.282617 |
| 67. | 1. | 0. | 0.106598 | 3.256092 | 1.487069 |

| **2c** | | Standard Orientation  (Ångstroms) | | | |
| --- | --- | --- | --- | --- | --- |
| Center number | Atomic number | Atomic Type | X | Y | Z |
| 1. | 6. | 0. | -2.394388 | 2.106339 | -0.615832 |
| 2. | 6. | 0. | -3.772496 | 2.056466 | -0.366207 |
| 3. | 6. | 0. | -4.416494 | 0.831606 | -0.172045 |
| 4. | 6. | 0. | -3.722495 | -0.406452 | -0.238686 |
| 5. | 8. | 0. | -5.767869 | 0.856852 | 0.005798 |
| 6. | 6. | 0. | -6.423068 | -0.254227 | 0.708946 |
| 7. | 6. | 0. | -5.779844 | -1.562968 | 0.312795 |
| 8. | 6. | 0. | -4.504668 | -1.633127 | -0.101485 |
| 9. | 6. | 0. | -7.883724 | -0.186103 | 0.261447 |
| 10. | 6. | 0. | -6.290480 | -0.013626 | 2.221262 |
| 11. | 6. | 0. | 2.264636 | -0.659261 | -0.973441 |
| 12. | 6. | 0. | 2.189752 | 0.610188 | -0.033752 |
| 13. | 6. | 0. | 3.711897 | -1.220472 | -0.910047 |
| 14. | 6. | 0. | 4.595707 | -0.262575 | -0.064628 |
| 15. | 7. | 0. | 3.983582 | -0.192799 | 1.285819 |
| 16. | 6. | 0. | 2.719952 | 0.245398 | 1.386126 |
| 17. | 8. | 0. | 2.070384 | 0.350105 | 2.434428 |
| 18. | 6. | 0. | 6.028058 | -0.734082 | 0.214701 |
| 19. | 6. | 0. | 5.943870 | -1.442090 | 1.584176 |
| 20. | 6. | 0. | 4.876438 | -0.650331 | 2.357148 |
| 21. | 6. | 0. | 4.462094 | 1.141959 | -0.688357 |
| 22. | 7. | 0. | 3.175109 | 1.550241 | -0.591800 |
| 23. | 8. | 0. | 5.377900 | 1.784919 | -1.206772 |
| 24. | 6. | 0. | 0.772907 | 1.265148 | 0.045324 |
| 25. | 6. | 0. | -0.221628 | 0.619145 | -0.942237 |
| 26. | 6. | 0. | -0.166760 | -0.889160 | -0.797959 |
| 27. | 6. | 0. | 1.112115 | -1.695792 | -0.791141 |
| 28. | 6. | 0. | -1.683691 | 0.913855 | -0.654142 |
| 29. | 6. | 0. | -2.351477 | -0.294134 | -0.476225 |
| 30. | 7. | 0. | -1.374311 | -1.369924 | -0.572338 |
| 31. | 6. | 0. | 1.208818 | -2.514927 | 0.522297 |
| 32. | 6. | 0. | 1.129919 | -2.670507 | -1.998268 |
| 33. | 8. | 0. | -1.708923 | -2.601931 | -0.411424 |
| 34. | 8. | 0. | 0.127007 | 0.980062 | -2.291860 |
| 35. | 8. | 0. | 0.803807 | 2.655072 | -0.259944 |
| 36. | 6. | 0. | 0.909346 | 3.532177 | 0.871701 |
| 37. | 1. | 0. | 2.120256 | -0.235444 | -1.968876 |
| 38. | 1. | 0. | -1.901427 | 3.060998 | -0.763089 |
| 39. | 1. | 0. | -4.361145 | 2.966483 | -0.317348 |
| 40. | 1. | 0. | -6.382848 | -2.460682 | 0.419050 |
| 41. | 1. | 0. | -4.031970 | -2.575381 | -0.343599 |
| 42. | 1. | 0. | -8.465529 | -0.961456 | 0.768490 |
| 43. | 1. | 0. | -8.310788 | 0.788741 | 0.514576 |
| 44. | 1. | 0. | -7.965859 | -0.338788 | -0.818469 |
| 45. | 1. | 0. | -6.779159 | -0.822116 | 2.774260 |
| 46. | 1. | 0. | -6.764465 | 0.934119 | 2.496815 |
| 47. | 1. | 0. | -5.237982 | 0.014952 | 2.518763 |
| 48. | 1. | 0. | 4.133559 | -1.319223 | -1.914117 |
| 49. | 1. | 0. | 3.753433 | -2.205460 | -0.439038 |
| 50. | 1. | 0. | 6.400830 | -1.387997 | -0.577057 |
| 51. | 1. | 0. | 6.688091 | 0.136793 | 0.266113 |
| 52. | 1. | 0. | 5.618730 | -2.480137 | 1.460317 |
| 53. | 1. | 0. | 6.903584 | -1.452061 | 2.105667 |
| 54. | 1. | 0. | 5.306263 | 0.211987 | 2.881556 |
| 55. | 1. | 0. | 4.318894 | -1.250595 | 3.079667 |
| 56. | 1. | 0. | 2.858071 | 2.434878 | -0.971966 |
| 57. | 1. | 0. | 0.402874 | 1.110365 | 1.064116 |
| 58. | 1. | 0. | 0.363624 | -3.201603 | 0.587537 |
| 59. | 1. | 0. | 2.127524 | -3.107485 | 0.535671 |
| 60. | 1. | 0. | 1.197488 | -1.877018 | 1.409087 |
| 61. | 1. | 0. | 0.288115 | -3.364004 | -1.936959 |
| 62. | 1. | 0. | 2.055955 | -3.254447 | -1.996347 |
| 63. | 1. | 0. | 1.067811 | -2.126138 | -2.945642 |
| 64. | 1. | 0. | 0.201037 | 1.948297 | -2.312235 |
| 65. | 1. | 0. | 0.872012 | 4.547782 | 0.475430 |
| 66. | 1. | 0. | 1.855219 | 3.382327 | 1.403719 |
| 67. | 1. | 0. | 0.072895 | 3.374357 | 1.561500 |

| **2d** | | Standard Orientation  (Ångstroms) | | | |
| --- | --- | --- | --- | --- | --- |
| Center number | Atomic number | Atomic Type | X | Y | Z |
| 1. | 6. | 0. | -2.392310 | 2.066581 | -0.762332 |
| 2. | 6. | 0. | -3.771441 | 2.034884 | -0.518665 |
| 3. | 6. | 0. | -4.417079 | 0.825926 | -0.243512 |
| 4. | 6. | 0. | -3.725486 | -0.415600 | -0.224311 |
| 5. | 8. | 0. | -5.768549 | 0.865185 | -0.072894 |
| 6. | 6. | 0. | -6.429610 | -0.195934 | 0.699219 |
| 7. | 6. | 0. | -5.788223 | -1.529204 | 0.392104 |
| 8. | 6. | 0. | -4.511244 | -1.628897 | -0.010409 |
| 9. | 6. | 0. | -7.888499 | -0.153561 | 0.242877 |
| 10. | 6. | 0. | -6.301282 | 0.143435 | 2.192676 |
| 11. | 6. | 0. | 2.257147 | -0.709139 | -0.933703 |
| 12. | 6. | 0. | 2.179833 | 0.598109 | -0.048195 |
| 13. | 6. | 0. | 3.710270 | -1.250818 | -0.868048 |
| 14. | 6. | 0. | 4.598998 | -0.229438 | -0.104874 |
| 15. | 7. | 0. | 4.025852 | -0.102412 | 1.258405 |
| 16. | 6. | 0. | 2.752994 | 0.304979 | 1.371977 |
| 17. | 8. | 0. | 2.124328 | 0.429897 | 2.430809 |
| 18. | 6. | 0. | 6.047829 | -0.659284 | 0.154939 |
| 19. | 6. | 0. | 6.011782 | -1.311702 | 1.553760 |
| 20. | 6. | 0. | 4.953659 | -0.503544 | 2.322727 |
| 21. | 6. | 0. | 4.419122 | 1.142229 | -0.791089 |
| 22. | 7. | 0. | 3.128123 | 1.530700 | -0.676177 |
| 23. | 8. | 0. | 5.309544 | 1.778050 | -1.360750 |
| 24. | 6. | 0. | 0.751834 | 1.225059 | 0.038269 |
| 25. | 6. | 0. | -0.220686 | 0.569226 | -0.961017 |
| 26. | 6. | 0. | -0.169473 | -0.938408 | -0.738793 |
| 27. | 6. | 0. | 1.111108 | -1.742410 | -0.699355 |
| 28. | 6. | 0. | -1.684534 | 0.872617 | -0.715695 |
| 29. | 6. | 0. | -2.353420 | -0.322615 | -0.463394 |
| 30. | 7. | 0. | -1.378140 | -1.405482 | -0.494395 |
| 31. | 6. | 0. | 1.217242 | -2.500394 | 0.648933 |
| 32. | 6. | 0. | 1.127658 | -2.770748 | -1.860943 |
| 33. | 8. | 0. | -1.719408 | -2.628748 | -0.278206 |
| 34. | 8. | 0. | 0.200092 | 0.955554 | -2.279586 |
| 35. | 8. | 0. | 0.759397 | 2.626469 | -0.177500 |
| 36. | 6. | 0. | 0.905616 | 3.421030 | 1.006751 |
| 37. | 1. | 0. | 2.099958 | -0.325819 | -1.942983 |
| 38. | 1. | 0. | -1.889908 | 3.004278 | -0.970829 |
| 39. | 1. | 0. | -4.360481 | 2.945816 | -0.535838 |
| 40. | 1. | 0. | -6.394179 | -2.416661 | 0.553298 |
| 41. | 1. | 0. | -4.039007 | -2.585637 | -0.188180 |
| 42. | 1. | 0. | -8.474633 | -0.890303 | 0.800058 |
| 43. | 1. | 0. | -8.313168 | 0.837685 | 0.426720 |
| 44. | 1. | 0. | -7.967819 | -0.379910 | -0.824265 |
| 45. | 1. | 0. | -6.796248 | -0.624473 | 2.795593 |
| 46. | 1. | 0. | -6.771246 | 1.109627 | 2.403444 |
| 47. | 1. | 0. | -5.249745 | 0.186299 | 2.491794 |
| 48. | 1. | 0. | 4.110409 | -1.409049 | -1.873331 |
| 49. | 1. | 0. | 3.774296 | -2.203176 | -0.335795 |
| 50. | 1. | 0. | 6.414321 | -1.337558 | -0.619100 |
| 51. | 1. | 0. | 6.690050 | 0.226326 | 0.153894 |
| 52. | 1. | 0. | 5.698108 | -2.358054 | 1.480097 |
| 53. | 1. | 0. | 6.985218 | -1.287538 | 2.048707 |
| 54. | 1. | 0. | 5.384740 | 0.385408 | 2.799377 |
| 55. | 1. | 0. | 4.424497 | -1.081092 | 3.084095 |
| 56. | 1. | 0. | 2.783809 | 2.402542 | -1.062528 |
| 57. | 1. | 0. | 0.376592 | 1.004912 | 1.044029 |
| 58. | 1. | 0. | 0.373162 | -3.184139 | 0.752035 |
| 59. | 1. | 0. | 2.137238 | -3.090630 | 0.681850 |
| 60. | 1. | 0. | 1.213154 | -1.822355 | 1.505504 |
| 61. | 1. | 0. | 0.286395 | -3.461365 | -1.767335 |
| 62. | 1. | 0. | 2.054229 | -3.353295 | -1.834036 |
| 63. | 1. | 0. | 1.064265 | -2.270393 | -2.832380 |
| 64. | 1. | 0. | -0.453413 | 0.635648 | -2.919154 |
| 65. | 1. | 0. | 0.111271 | 3.193458 | 1.727173 |
| 66. | 1. | 0. | 0.822538 | 4.461204 | 0.687751 |
| 67. | 1. | 0. | 1.880705 | 3.263081 | 1.481151 |

**ECD calculation details of compound 3**

1. Methods

In general, conformational analyses were carried out via random searching in the Sybyl-X 2.0 using the MMFF94S force field with an energy cutoff of 2.5 kcal/mol. The results showed tei lowest energy conformer for both compounds. Subsequently, the conformers were re-optimized using DFT at the PBE0-D3/def2-SVP level in MeOH using the polarizable conductor calculation model (SMD) by the GAUSSIAN 09 program. The energies, oscillator strengths, and rotational strengths (velocity) of the first 30 electronic excitations were calculated using the TDDFT methodology at the PBE0-D3/def2-TZVP level in MeOH. The ECD spectra were simulated by the overlapping Gaussian function (half the bandwidth at 1/e peak height, sigma = 0.30 for all). To get the ﬁnal spectra, the simulated spectra of the conformers were averaged according to the Boltzmann distribution theory and their relative Gibbs free energy (∆G). By comparing the experiment spectra with the calculated model molecules, the absolute configuration of the only chiral center was determined to be.

2. Results

Table S1.2.1.Gibbs free energies^a^ and equilibrium populations^b^ of low-energy conformers of **3**.

| Conformers | ∆G | P(%)/100 |
| --- | --- | --- |
| **3a** | 0.00088 | 6.47 |
| **3b** | 0.00073 | 7.53 |
| **3c** | 0.00074 | 7.46 |
| **3d** | 0.0007 | 7.81 |
| **3e** | 4e-05 | 15.65 |
| **3f** | 0.0 | 16.37 |
| **3g** | 5e-05 | 15.48 |
| **3h** | 5e-05 | 15.48 |
| **3i** | 0.00071 | 7.75 |

*^a^*PBE0-D3/def2-TZVP, in kcal/mol. *^b^*From ∆G values at 298.15K.

Table S1.2.2.Cartesian coordinates for the low-energy reoptimized random reseach conformers of **3** at PBE0-D3/def2-SVP level of theory in CH_3_OH.

| **3a** | | Standard Orientation (Ångstroms) | | | |
| --- | --- | --- | --- | --- | --- |
| Center number | Atomic number | Atomic Type | X | Y | Z |
| 0 | 6 | 0 | 13.103834 | -3.750627 | 2.687239 |
| 1 | 6 | 0 | 11.296341 | -4.301341 | -1.668188 |
| 2 | 6 | 0 | 6.262176 | -3.171875 | 1.809949 |
| 3 | 6 | 0 | 8.466948 | -4.364 | 2.070535 |
| 4 | 6 | 0 | 10.852803 | -3.296048 | 0.983543 |
| 5 | 8 | 0 | 10.669683 | -0.505919 | 0.794459 |
| 6 | 6 | 0 | 6.160316 | -0.721078 | 0.618941 |
| 7 | 6 | 0 | 8.436866 | 0.563102 | 0.181538 |
| 8 | 6 | 0 | 8.534009 | 3.041048 | -0.772063 |
| 9 | 7 | 0 | 1.513642 | -0.257031 | 0.193603 |
| 10 | 6 | 0 | 3.942976 | 0.576809 | -0.002809 |
| 11 | 6 | 0 | 4.01908 | 3.06422 | -0.959282 |
| 12 | 6 | 0 | 1.472089 | 3.971008 | -1.124906 |
| 13 | 6 | 0 | -2.100006 | 2.340178 | 1.79643 |
| 14 | 6 | 0 | -0.262384 | 1.743085 | -0.452857 |
| 15 | 6 | 0 | -2.070825 | 0.87672 | -2.678026 |
| 16 | 6 | 0 | -6.827705 | 2.618446 | 0.795342 |
| 17 | 6 | 0 | -4.526167 | 0.874157 | 1.224522 |
| 18 | 6 | 0 | -3.900605 | -0.73114 | -1.122027 |
| 19 | 6 | 0 | -6.3112 | -1.898598 | -2.167186 |
| 20 | 6 | 0 | -12.790002 | -0.577938 | 0.289183 |
| 21 | 6 | 0 | -11.431342 | 1.921211 | -0.146806 |
| 22 | 7 | 0 | -8.77998 | 1.208703 | 0.087772 |
| 23 | 6 | 0 | -8.372088 | -1.546045 | -0.142462 |
| 24 | 6 | 0 | -10.978298 | -2.550752 | -0.780358 |
| 25 | 8 | 0 | -6.866858 | 4.925338 | 1.164003 |
| 26 | 1 | 0 | -2.770691 | -2.279766 | -0.363541 |
| 27 | 6 | 0 | -7.334118 | -2.397 | 2.45063 |
| 28 | 7 | 0 | -5.388956 | -0.917539 | 3.134974 |
| 29 | 8 | 0 | -8.186932 | -4.160515 | 3.714168 |
| 30 | 6 | 0 | 6.324287 | 4.294764 | -1.324442 |
| 31 | 8 | 0 | 0.714752 | 6.115755 | -1.642947 |
| 32 | 6 | 0 | -0.705341 | -0.698236 | -4.651108 |
| 33 | 6 | 0 | -3.346061 | 3.095144 | -3.992207 |
| 34 | 8 | 0 | -1.179038 | 1.664382 | 4.19441 |
| 35 | 6 | 0 | 0.588032 | 3.426807 | 5.21252 |
| 36 | 1 | 0 | 12.769362 | -2.999265 | 4.571871 |
| 37 | 1 | 0 | 13.457603 | -5.769919 | 2.853693 |
| 38 | 1 | 0 | 14.792615 | -2.870128 | 1.910832 |
| 39 | 1 | 0 | 13.024341 | -3.517787 | -2.466934 |
| 40 | 1 | 0 | 11.476924 | -6.350967 | -1.621951 |
| 41 | 1 | 0 | 9.7208 | -3.831672 | -2.90451 |
| 42 | 1 | 0 | 4.544387 | -4.012399 | 2.533218 |
| 43 | 1 | 0 | 8.597283 | -6.186172 | 2.986407 |
| 44 | 1 | 0 | 10.357512 | 3.915488 | -1.046475 |
| 45 | 1 | 0 | 1.045798 | -1.512566 | 1.541135 |
| 46 | 1 | 0 | -2.52574 | 4.358213 | 1.734284 |
| 47 | 1 | 0 | -6.979692 | -0.964181 | -3.872893 |
| 48 | 1 | 0 | -6.058979 | -3.891 | -2.598106 |
| 49 | 1 | 0 | -14.633449 | -0.620406 | -0.61457 |
| 50 | 1 | 0 | -13.060907 | -0.879204 | 2.307826 |
| 51 | 1 | 0 | -11.894553 | 3.375216 | 1.228398 |
| 52 | 1 | 0 | -11.788257 | 2.670298 | -2.034152 |
| 53 | 1 | 0 | -11.273568 | -4.42771 | -0.00377 |
| 54 | 1 | 0 | -11.165431 | -2.673653 | -2.826632 |
| 55 | 1 | 0 | -4.429483 | -1.163393 | 4.76373 |
| 56 | 1 | 0 | 6.365985 | 6.210306 | -2.036069 |
| 57 | 1 | 0 | -2.030123 | -1.266125 | -6.121916 |
| 58 | 1 | 0 | 0.794275 | 0.377036 | -5.564717 |
| 59 | 1 | 0 | 0.118963 | -2.400183 | -3.853217 |
| 60 | 1 | 0 | -4.864822 | 2.407845 | -5.200806 |
| 61 | 1 | 0 | -4.146555 | 4.490154 | -2.721934 |
| 62 | 1 | 0 | -2.013691 | 4.082148 | -5.204872 |
| 63 | 1 | 0 | 2.310893 | 3.519988 | 4.085654 |
| 64 | 1 | 0 | -0.2451 | 5.310437 | 5.33556 |
| 65 | 1 | 0 | 1.064013 | 2.763911 | 7.0956 |

| **3b** | | Standard Orientation (Ångstroms) | | | |
| --- | --- | --- | --- | --- | --- |
| Center number | Atomic number | Atomic Type | X | Y | Z |
| 0 | 6 | 0 | 12.939528 | -4.710795 | 1.390879 |
| 1 | 6 | 0 | 11.103924 | -3.635335 | -2.853951 |
| 2 | 6 | 0 | 6.102997 | -3.741735 | 0.807413 |
| 3 | 6 | 0 | 8.289247 | -4.98559 | 0.644786 |
| 4 | 6 | 0 | 10.691093 | -3.634762 | -0.011125 |
| 5 | 8 | 0 | 10.557064 | -0.967759 | 0.8153 |
| 6 | 6 | 0 | 6.04889 | -1.026807 | 0.534787 |
| 7 | 6 | 0 | 8.348648 | 0.290271 | 0.593899 |
| 8 | 6 | 0 | 8.488562 | 2.942221 | 0.567325 |
| 9 | 7 | 0 | 1.416727 | -0.367184 | 0.234973 |
| 10 | 6 | 0 | 3.857192 | 0.442051 | 0.376821 |
| 11 | 6 | 0 | 3.979032 | 3.104005 | 0.351393 |
| 12 | 6 | 0 | 1.443075 | 4.056652 | 0.463113 |
| 13 | 6 | 0 | -2.257445 | 1.567164 | 2.499539 |
| 14 | 6 | 0 | -0.326259 | 1.761056 | 0.26436 |
| 15 | 6 | 0 | -2.037567 | 1.757972 | -2.20116 |
| 16 | 6 | 0 | -6.933219 | 2.282983 | 1.462422 |
| 17 | 6 | 0 | -4.663368 | 0.444868 | 1.350475 |
| 18 | 6 | 0 | -3.936949 | -0.258796 | -1.378085 |
| 19 | 6 | 0 | -6.302934 | -0.947727 | -2.863867 |
| 20 | 6 | 0 | -12.877557 | -0.439119 | -0.328706 |
| 21 | 6 | 0 | -11.499575 | 2.035967 | 0.183323 |
| 22 | 7 | 0 | -8.854676 | 1.256589 | 0.209379 |
| 23 | 6 | 0 | -8.454009 | -1.260465 | -0.931022 |
| 24 | 6 | 0 | -11.036338 | -1.93389 | -1.972883 |
| 25 | 8 | 0 | -6.981174 | 4.307344 | 2.626964 |
| 26 | 1 | 0 | -2.846114 | -1.993116 | -1.158616 |
| 27 | 6 | 0 | -7.546271 | -2.983717 | 1.250499 |
| 28 | 7 | 0 | -5.631217 | -1.869909 | 2.493144 |
| 29 | 8 | 0 | -8.461639 | -5.061361 | 1.779522 |
| 30 | 6 | 0 | 6.302446 | 4.347136 | 0.463042 |
| 31 | 8 | 0 | 0.722005 | 6.258895 | 0.721503 |
| 32 | 6 | 0 | -0.589721 | 0.94626 | -4.543812 |
| 33 | 6 | 0 | -3.247824 | 4.319523 | -2.701021 |
| 34 | 8 | 0 | -1.445367 | 0.063418 | 4.536479 |
| 35 | 6 | 0 | 0.306414 | 1.301823 | 6.166835 |
| 36 | 1 | 0 | 14.64023 | -3.634393 | 0.967683 |
| 37 | 1 | 0 | 12.624234 | -4.678926 | 3.423049 |
| 38 | 1 | 0 | 13.25208 | -6.660297 | 0.817155 |
| 39 | 1 | 0 | 9.524519 | -2.740058 | -3.821559 |
| 40 | 1 | 0 | 12.836347 | -2.634 | -3.339238 |
| 41 | 1 | 0 | 11.256967 | -5.568778 | -3.540876 |
| 42 | 1 | 0 | 4.366509 | -4.746346 | 1.204603 |
| 43 | 1 | 0 | 8.395304 | -7.011709 | 0.890048 |
| 44 | 1 | 0 | 10.32612 | 3.828929 | 0.63478 |
| 45 | 1 | 0 | 0.899376 | -2.007346 | 1.04268 |
| 46 | 1 | 0 | -2.671137 | 3.479632 | 3.152017 |
| 47 | 1 | 0 | -6.883394 | 0.526245 | -4.173161 |
| 48 | 1 | 0 | -6.043195 | -2.678684 | -3.937977 |
| 49 | 1 | 0 | -14.691222 | -0.136269 | -1.242496 |
| 50 | 1 | 0 | -13.211249 | -1.429926 | 1.444892 |
| 51 | 1 | 0 | -11.99609 | 2.9125 | 1.973049 |
| 52 | 1 | 0 | -11.809546 | 3.409764 | -1.321326 |
| 53 | 1 | 0 | -11.360919 | -3.959141 | -1.917849 |
| 54 | 1 | 0 | -11.153701 | -1.318301 | -3.933234 |
| 55 | 1 | 0 | -4.722938 | -2.707085 | 3.944732 |
| 56 | 1 | 0 | 6.376865 | 6.390001 | 0.465088 |
| 57 | 1 | 0 | -1.843371 | 0.985728 | -6.177516 |
| 58 | 1 | 0 | 0.973403 | 2.225647 | -4.939313 |
| 59 | 1 | 0 | 0.163543 | -0.955638 | -4.370837 |
| 60 | 1 | 0 | -1.851168 | 5.658196 | -3.389055 |
| 61 | 1 | 0 | -4.685028 | 4.140162 | -4.162374 |
| 62 | 1 | 0 | -4.130562 | 5.162747 | -1.05451 |
| 63 | 1 | 0 | 0.663665 | 0.022542 | 7.730904 |
| 64 | 1 | 0 | 2.090227 | 1.701373 | 5.21627 |
| 65 | 1 | 0 | -0.48209 | 3.066403 | 6.889119 |

| **3c** | | Standard Orientation (Ångstroms) | | | |
| --- | --- | --- | --- | --- | --- |
| Center number | Atomic number | Atomic Type | X | Y | Z |
| 0 | 6 | 0 | 13.087612 | -4.596031 | 1.295868 |
| 1 | 6 | 0 | 11.228305 | -3.604107 | -2.959409 |
| 2 | 6 | 0 | 6.246274 | -3.652467 | 0.729056 |
| 3 | 6 | 0 | 8.434351 | -4.894745 | 0.578235 |
| 4 | 6 | 0 | 10.829837 | -3.551787 | -0.114999 |
| 5 | 8 | 0 | 10.694147 | -0.869774 | 0.662026 |
| 6 | 6 | 0 | 6.184867 | -0.943185 | 0.405327 |
| 7 | 6 | 0 | 8.482218 | 0.379332 | 0.428384 |
| 8 | 6 | 0 | 8.616025 | 3.030662 | 0.349896 |
| 9 | 7 | 0 | 1.550721 | -0.298823 | 0.108822 |
| 10 | 6 | 0 | 3.989234 | 0.517309 | 0.225832 |
| 11 | 6 | 0 | 4.105531 | 3.178665 | 0.144996 |
| 12 | 6 | 0 | 1.567882 | 4.128171 | 0.240052 |
| 13 | 6 | 0 | -2.139921 | 1.677127 | 2.313334 |
| 14 | 6 | 0 | -0.196612 | 1.824538 | 0.085592 |
| 15 | 6 | 0 | -1.89452 | 1.766716 | -2.389268 |
| 16 | 6 | 0 | -6.809412 | 2.371303 | 1.234827 |
| 17 | 6 | 0 | -4.539737 | 0.530188 | 1.17571 |
| 18 | 6 | 0 | -3.798317 | -0.232224 | -1.532698 |
| 19 | 6 | 0 | -6.155864 | -0.952431 | -3.016721 |
| 20 | 6 | 0 | -12.745249 | -0.382588 | -0.537304 |
| 21 | 6 | 0 | -11.366863 | 2.101601 | -0.073343 |
| 22 | 7 | 0 | -8.724079 | 1.31788 | -0.006188 |
| 23 | 6 | 0 | -8.317976 | -1.222892 | -1.0898 |
| 24 | 6 | 0 | -10.894448 | -1.918185 | -2.132552 |
| 25 | 8 | 0 | -6.863268 | 4.421252 | 2.353803 |
| 26 | 1 | 0 | -2.708598 | -1.961028 | -1.269935 |
| 27 | 6 | 0 | -7.421719 | -2.900171 | 1.132099 |
| 28 | 7 | 0 | -5.515202 | -1.758664 | 2.363192 |
| 29 | 8 | 0 | -8.337461 | -4.968198 | 1.697631 |
| 30 | 6 | 0 | 6.426543 | 4.428633 | 0.225036 |
| 31 | 8 | 0 | 0.841834 | 6.333797 | 0.454827 |
| 32 | 6 | 0 | -0.435907 | 0.902703 | -4.706025 |
| 33 | 6 | 0 | -3.100792 | 4.317641 | -2.949611 |
| 34 | 8 | 0 | -1.339204 | 0.218226 | 4.386946 |
| 35 | 6 | 0 | 0.41321 | 1.487854 | 5.9925 |
| 36 | 1 | 0 | 12.78121 | -4.52758 | 3.328512 |
| 37 | 1 | 0 | 13.402853 | -6.55488 | 0.756784 |
| 38 | 1 | 0 | 14.783252 | -3.522895 | 0.84495 |
| 39 | 1 | 0 | 12.956601 | -2.609265 | -3.471927 |
| 40 | 1 | 0 | 11.38018 | -5.54959 | -3.61179 |
| 41 | 1 | 0 | 9.642268 | -2.728829 | -3.934414 |
| 42 | 1 | 0 | 4.51449 | -4.653743 | 1.154329 |
| 43 | 1 | 0 | 8.545596 | -6.915826 | 0.860428 |
| 44 | 1 | 0 | 10.45195 | 3.922364 | 0.392787 |
| 45 | 1 | 0 | 1.035963 | -1.927795 | 0.939814 |
| 46 | 1 | 0 | -2.557841 | 3.602924 | 2.922456 |
| 47 | 1 | 0 | -6.727847 | 0.493802 | -4.360005 |
| 48 | 1 | 0 | -5.89129 | -2.705606 | -4.052772 |
| 49 | 1 | 0 | -14.551529 | -0.098281 | -1.471343 |
| 50 | 1 | 0 | -13.09406 | -1.330752 | 1.256732 |
| 51 | 1 | 0 | -11.877262 | 3.022925 | 1.689641 |
| 52 | 1 | 0 | -11.660948 | 3.438094 | -1.614343 |
| 53 | 1 | 0 | -11.22191 | -3.941178 | -2.03192 |
| 54 | 1 | 0 | -10.997912 | -1.349673 | -4.107772 |
| 55 | 1 | 0 | -4.60931 | -2.570433 | 3.830671 |
| 56 | 1 | 0 | 6.49653 | 6.471287 | 0.185266 |
| 57 | 1 | 0 | -1.682415 | 0.906559 | -6.345742 |
| 58 | 1 | 0 | 1.13003 | 2.171619 | -5.123251 |
| 59 | 1 | 0 | 0.314361 | -0.995835 | -4.48733 |
| 60 | 1 | 0 | -1.700953 | 5.636652 | -3.668886 |
| 61 | 1 | 0 | -4.538777 | 4.109137 | -4.406357 |
| 62 | 1 | 0 | -3.97972 | 5.200011 | -1.321674 |
| 63 | 1 | 0 | 2.204568 | 1.849361 | 5.04081 |
| 64 | 1 | 0 | -0.365462 | 3.277105 | 6.663002 |
| 65 | 1 | 0 | 0.754173 | 0.248691 | 7.592081 |

| **3d** | | Standard Orientation (Ångstroms) | | | |
| --- | --- | --- | --- | --- | --- |
| Center number | Atomic number | Atomic Type | X | Y | Z |
| 0 | 6 | 0 | 13.105797 | -4.083451 | 2.629747 |
| 1 | 6 | 0 | 11.081092 | -4.446075 | -1.648023 |
| 2 | 6 | 0 | 6.242652 | -3.299628 | 2.036093 |
| 3 | 6 | 0 | 8.427981 | -4.542468 | 2.218671 |
| 4 | 6 | 0 | 10.792466 | -3.50771 | 1.050876 |
| 5 | 8 | 0 | 10.677015 | -0.715755 | 0.960036 |
| 6 | 6 | 0 | 6.1633 | -0.838196 | 0.860826 |
| 7 | 6 | 0 | 8.456432 | 0.407033 | 0.392457 |
| 8 | 6 | 0 | 8.580828 | 2.888789 | -0.543346 |
| 9 | 7 | 0 | 1.519594 | -0.297067 | 0.505899 |
| 10 | 6 | 0 | 3.959158 | 0.499239 | 0.279988 |
| 11 | 6 | 0 | 4.065657 | 2.988555 | -0.665949 |
| 12 | 6 | 0 | 1.527958 | 3.934463 | -0.813307 |
| 13 | 6 | 0 | -2.110863 | 2.30585 | 2.051402 |
| 14 | 6 | 0 | -0.231253 | 1.718485 | -0.158245 |
| 15 | 6 | 0 | -1.991085 | 0.868069 | -2.434444 |
| 16 | 6 | 0 | -6.803597 | 2.659835 | 0.914609 |
| 17 | 6 | 0 | -4.539996 | 0.877277 | 1.403337 |
| 18 | 6 | 0 | -3.867045 | -0.732643 | -0.926023 |
| 19 | 6 | 0 | -6.263631 | -1.871474 | -2.041478 |
| 20 | 6 | 0 | -12.786259 | -0.459259 | 0.279184 |
| 21 | 6 | 0 | -11.393889 | 2.027449 | -0.122901 |
| 22 | 7 | 0 | -8.751259 | 1.281006 | 0.126079 |
| 23 | 6 | 0 | -8.378698 | -1.477512 | -0.084585 |
| 24 | 6 | 0 | -10.982799 | -2.444472 | -0.786124 |
| 25 | 8 | 0 | -6.82553 | 4.961435 | 1.311114 |
| 26 | 1 | 0 | -2.768024 | -2.290892 | -0.145193 |
| 27 | 6 | 0 | -7.426951 | -2.35726 | 2.536246 |
| 28 | 7 | 0 | -5.492708 | -0.893052 | 3.292001 |
| 29 | 8 | 0 | -8.32446 | -4.130318 | 3.754151 |
| 30 | 6 | 0 | 6.384266 | 4.18325 | -1.057436 |
| 31 | 8 | 0 | 0.799895 | 6.091913 | -1.313994 |
| 32 | 6 | 0 | -0.581188 | -0.704596 | -4.378825 |
| 33 | 6 | 0 | -3.225648 | 3.096766 | -3.773018 |
| 34 | 8 | 0 | -1.248182 | 1.572172 | 4.457202 |
| 35 | 6 | 0 | 0.557375 | 3.268827 | 5.518556 |
| 36 | 1 | 0 | 12.880238 | -3.384677 | 4.551031 |
| 37 | 1 | 0 | 13.405221 | -6.11596 | 2.713696 |
| 38 | 1 | 0 | 14.780971 | -3.221969 | 1.803609 |
| 39 | 1 | 0 | 12.785156 | -3.673857 | -2.507582 |
| 40 | 1 | 0 | 11.215393 | -6.499049 | -1.667544 |
| 41 | 1 | 0 | 9.457027 | -3.905456 | -2.789447 |
| 42 | 1 | 0 | 4.527909 | -4.097621 | 2.813868 |
| 43 | 1 | 0 | 8.555073 | -6.368196 | 3.128269 |
| 44 | 1 | 0 | 10.415117 | 3.734081 | -0.841068 |
| 45 | 1 | 0 | 1.037349 | -1.52932 | 1.869887 |
| 46 | 1 | 0 | -2.508996 | 4.329033 | 2.026033 |
| 47 | 1 | 0 | -6.856402 | -0.936256 | -3.773695 |
| 48 | 1 | 0 | -6.02712 | -3.869899 | -2.451738 |
| 49 | 1 | 0 | -14.620376 | -0.47479 | -0.64349 |
| 50 | 1 | 0 | -13.078323 | -0.774452 | 2.292678 |
| 51 | 1 | 0 | -11.851757 | 3.474768 | 1.260629 |
| 52 | 1 | 0 | -11.729954 | 2.795463 | -2.005544 |
| 53 | 1 | 0 | -11.312211 | -4.325618 | -0.035605 |
| 54 | 1 | 0 | -11.136109 | -2.535507 | -2.836307 |
| 55 | 1 | 0 | -4.539666 | -1.210087 | 4.912515 |
| 56 | 1 | 0 | 6.445757 | 6.101818 | -1.760287 |
| 57 | 1 | 0 | -1.853511 | -1.205511 | -5.918382 |
| 58 | 1 | 0 | 0.984949 | 0.35224 | -5.197829 |
| 59 | 1 | 0 | 0.16296 | -2.442945 | -3.579901 |
| 60 | 1 | 0 | -4.740743 | 2.423572 | -4.993213 |
| 61 | 1 | 0 | -4.01781 | 4.505691 | -2.512652 |
| 62 | 1 | 0 | -1.868241 | 4.061684 | -4.975743 |
| 63 | 1 | 0 | 2.301728 | 3.313422 | 4.421768 |
| 64 | 1 | 0 | -0.213282 | 5.178971 | 5.640091 |
| 65 | 1 | 0 | 0.978836 | 2.580459 | 7.405089 |

| **3e** | | Standard Orientation (Ångstroms) | | | |
| --- | --- | --- | --- | --- | --- |
| Center number | Atomic number | Atomic Type | X | Y | Z |
| 0 | 6 | 0 | 11.179112 | -2.494141 | 3.786765 |
| 1 | 6 | 0 | 12.975982 | -4.740305 | 0.010356 |
| 2 | 6 | 0 | 6.137293 | -3.698704 | 0.37637 |
| 3 | 6 | 0 | 8.338965 | -4.824395 | 0.865917 |
| 4 | 6 | 0 | 10.731663 | -3.321962 | 1.073284 |
| 5 | 8 | 0 | 10.561123 | -1.009405 | -0.490959 |
| 6 | 6 | 0 | 6.058289 | -1.021982 | -0.145984 |
| 7 | 6 | 0 | 8.341679 | 0.241922 | -0.61159 |
| 8 | 6 | 0 | 8.445831 | 2.790521 | -1.350334 |
| 9 | 7 | 0 | 1.417702 | -0.394814 | -0.092046 |
| 10 | 6 | 0 | 3.851557 | 0.404818 | -0.402749 |
| 11 | 6 | 0 | 3.940181 | 2.979658 | -1.075978 |
| 12 | 6 | 0 | 1.416747 | 3.965189 | -0.931577 |
| 13 | 6 | 0 | -2.054224 | 2.051085 | 1.97126 |
| 14 | 6 | 0 | -0.332714 | 1.707056 | -0.409627 |
| 15 | 6 | 0 | -2.253487 | 1.14349 | -2.645254 |
| 16 | 6 | 0 | -6.812421 | 2.527747 | 1.219977 |
| 17 | 6 | 0 | -4.532587 | 0.703377 | 1.334908 |
| 18 | 6 | 0 | -4.036344 | -0.620289 | -1.210418 |
| 19 | 6 | 0 | -6.512998 | -1.620473 | -2.280414 |
| 20 | 6 | 0 | -12.849694 | -0.501035 | 0.630804 |
| 21 | 6 | 0 | -11.468251 | 2.016422 | 0.434427 |
| 22 | 7 | 0 | -8.820028 | 1.247998 | 0.415126 |
| 23 | 6 | 0 | -8.48119 | -1.465514 | -0.144191 |
| 24 | 6 | 0 | -11.133581 | -2.349477 | -0.771794 |
| 25 | 8 | 0 | -6.789543 | 4.767187 | 1.883275 |
| 26 | 1 | 0 | -2.902761 | -2.263077 | -0.69969 |
| 27 | 6 | 0 | -7.352769 | -2.646657 | 2.280742 |
| 28 | 7 | 0 | -5.361907 | -1.276361 | 3.067132 |
| 29 | 8 | 0 | -8.171202 | -4.554435 | 3.340394 |
| 30 | 6 | 0 | 6.246501 | 4.164321 | -1.560452 |
| 31 | 8 | 0 | 0.694948 | 6.171637 | -1.143073 |
| 32 | 6 | 0 | -0.996269 | -0.186028 | -4.858326 |
| 33 | 6 | 0 | -3.553776 | 3.521458 | -3.616865 |
| 34 | 8 | 0 | -1.042744 | 1.059035 | 4.221747 |
| 35 | 6 | 0 | 0.875352 | 2.604089 | 5.317468 |
| 36 | 1 | 0 | 12.902926 | -1.377657 | 3.930975 |
| 37 | 1 | 0 | 9.600225 | -1.37304 | 4.481235 |
| 38 | 1 | 0 | 11.370408 | -4.144281 | 5.001135 |
| 39 | 1 | 0 | 14.670052 | -3.575844 | 0.081596 |
| 40 | 1 | 0 | 13.319071 | -6.439158 | 1.1165 |
| 41 | 1 | 0 | 12.636436 | -5.298413 | -1.939885 |
| 42 | 1 | 0 | 4.402862 | -4.779026 | 0.290404 |
| 43 | 1 | 0 | 8.46734 | -6.835563 | 1.206092 |
| 44 | 1 | 0 | 10.270426 | 3.634151 | -1.707353 |
| 45 | 1 | 0 | 1.034207 | -1.701578 | 1.234603 |
| 46 | 1 | 0 | -2.435923 | 4.065203 | 2.198595 |
| 47 | 1 | 0 | -7.225958 | -0.489882 | -3.842379 |
| 48 | 1 | 0 | -6.321221 | -3.556061 | -2.940213 |
| 49 | 1 | 0 | -14.73842 | -0.408759 | -0.169445 |
| 50 | 1 | 0 | -13.019125 | -1.049616 | 2.608067 |
| 51 | 1 | 0 | -11.822943 | 3.288718 | 2.007446 |
| 52 | 1 | 0 | -11.92267 | 3.004042 | -1.316264 |
| 53 | 1 | 0 | -11.422738 | -4.303919 | -0.218043 |
| 54 | 1 | 0 | -11.42408 | -2.209448 | -2.804265 |
| 55 | 1 | 0 | -4.29126 | -1.792176 | 4.558272 |
| 56 | 1 | 0 | 6.300869 | 6.143631 | -2.068566 |
| 57 | 1 | 0 | -2.381955 | -0.517777 | -6.34506 |
| 58 | 1 | 0 | 0.50331 | 0.970394 | -5.666728 |
| 59 | 1 | 0 | -0.192667 | -1.997423 | -4.322984 |
| 60 | 1 | 0 | -5.166031 | 3.005344 | -4.787299 |
| 61 | 1 | 0 | -4.228027 | 4.77128 | -2.139716 |
| 62 | 1 | 0 | -2.275083 | 4.615792 | -4.793962 |
| 63 | 1 | 0 | 2.569797 | 2.664668 | 4.145848 |
| 64 | 1 | 0 | 0.189312 | 4.527303 | 5.614122 |
| 65 | 1 | 0 | 1.352103 | 1.762391 | 7.127092 |

| **3f** | | Standard Orientation (Ångstroms) | | | |
| --- | --- | --- | --- | --- | --- |
| Center number | Atomic number | Atomic Type | X | Y | Z |
| 0 | 6 | 0 | 10.99638 | -3.263971 | 3.285253 |
| 1 | 6 | 0 | 13.081944 | -4.425554 | -0.816798 |
| 2 | 6 | 0 | 6.203528 | -3.532641 | -0.596829 |
| 3 | 6 | 0 | 8.398295 | -4.745242 | -0.334195 |
| 4 | 6 | 0 | 10.743495 | -3.347145 | 0.42512 |
| 5 | 8 | 0 | 10.617579 | -0.704152 | -0.482826 |
| 6 | 6 | 0 | 6.107209 | -0.81849 | -0.33818 |
| 7 | 6 | 0 | 8.389094 | 0.530922 | -0.338489 |
| 8 | 6 | 0 | 8.488976 | 3.184317 | -0.325635 |
| 9 | 7 | 0 | 1.460692 | -0.234265 | -0.268742 |
| 10 | 6 | 0 | 3.892297 | 0.618733 | -0.257991 |
| 11 | 6 | 0 | 3.974449 | 3.279231 | -0.164846 |
| 12 | 6 | 0 | 1.439286 | 4.178642 | 0.175136 |
| 13 | 6 | 0 | -2.043886 | 1.522192 | 2.373292 |
| 14 | 6 | 0 | -0.304337 | 1.860697 | -0.001231 |
| 15 | 6 | 0 | -2.202873 | 1.945786 | -2.320617 |
| 16 | 6 | 0 | -6.781091 | 2.287045 | 1.726642 |
| 17 | 6 | 0 | -4.534002 | 0.454184 | 1.361939 |
| 18 | 6 | 0 | -4.021249 | -0.123046 | -1.442423 |
| 19 | 6 | 0 | -6.495049 | -0.759139 | -2.769802 |
| 20 | 6 | 0 | -12.855477 | -0.350221 | 0.283336 |
| 21 | 6 | 0 | -11.432377 | 2.098711 | 0.797799 |
| 22 | 7 | 0 | -8.796476 | 1.318697 | 0.579152 |
| 23 | 6 | 0 | -8.496519 | -1.146271 | -0.69573 |
| 24 | 6 | 0 | -11.15367 | -1.767247 | -1.567419 |
| 25 | 8 | 0 | -6.732415 | 4.258081 | 2.978669 |
| 26 | 1 | 0 | -2.909825 | -1.857831 | -1.392089 |
| 27 | 6 | 0 | -7.43101 | -2.965608 | 1.330553 |
| 28 | 7 | 0 | -5.422245 | -1.908452 | 2.471586 |
| 29 | 8 | 0 | -8.308896 | -5.065845 | 1.83261 |
| 30 | 6 | 0 | 6.281599 | 4.556732 | -0.214802 |
| 31 | 8 | 0 | 0.704739 | 6.353714 | 0.581362 |
| 32 | 6 | 0 | -0.941306 | 1.243748 | -4.802539 |
| 33 | 6 | 0 | -3.466813 | 4.514628 | -2.62305 |
| 34 | 8 | 0 | -1.060686 | -0.096315 | 4.238776 |
| 35 | 6 | 0 | 0.794302 | 1.063325 | 5.812945 |
| 36 | 1 | 0 | 12.683702 | -2.220545 | 3.836371 |
| 37 | 1 | 0 | 9.353042 | -2.370105 | 4.141387 |
| 38 | 1 | 0 | 11.141697 | -5.175586 | 4.0321 |
| 39 | 1 | 0 | 14.7408 | -3.31152 | -0.32961 |
| 40 | 1 | 0 | 13.387832 | -6.352933 | -0.169273 |
| 41 | 1 | 0 | 12.881773 | -4.456209 | -2.863534 |
| 42 | 1 | 0 | 4.495264 | -4.547525 | -1.081139 |
| 43 | 1 | 0 | 8.549517 | -6.768158 | -0.582495 |
| 44 | 1 | 0 | 10.313779 | 4.098844 | -0.360253 |
| 45 | 1 | 0 | 1.046877 | -1.90018 | 0.547453 |
| 46 | 1 | 0 | -2.407314 | 3.388989 | 3.171055 |
| 47 | 1 | 0 | -7.166393 | 0.759278 | -3.981798 |
| 48 | 1 | 0 | -6.318112 | -2.450532 | -3.921031 |
| 49 | 1 | 0 | -14.734984 | -0.006714 | -0.468567 |
| 50 | 1 | 0 | -13.04866 | -1.423447 | 2.029305 |
| 51 | 1 | 0 | -11.782072 | 2.891673 | 2.65986 |
| 52 | 1 | 0 | -11.854337 | 3.540474 | -0.612962 |
| 53 | 1 | 0 | -11.482529 | -3.792473 | -1.58413 |
| 54 | 1 | 0 | -11.417077 | -1.05806 | -3.481484 |
| 55 | 1 | 0 | -4.399314 | -2.815887 | 3.799941 |
| 56 | 1 | 0 | 6.329443 | 6.598869 | -0.136196 |
| 57 | 1 | 0 | 0.576406 | 2.553019 | -5.271373 |
| 58 | 1 | 0 | -0.16452 | -0.655761 | -4.765479 |
| 59 | 1 | 0 | -2.322745 | 1.336085 | -6.327474 |
| 60 | 1 | 0 | -2.153782 | 5.879535 | -3.417282 |
| 61 | 1 | 0 | -5.046082 | 4.362726 | -3.933108 |
| 62 | 1 | 0 | -4.176361 | 5.313036 | -0.873612 |
| 63 | 1 | 0 | 1.285174 | -0.309476 | 7.256866 |
| 64 | 1 | 0 | 2.494985 | 1.557063 | 4.759901 |
| 65 | 1 | 0 | 0.037521 | 2.7612 | 6.707822 |

| **3g** | | Standard Orientation (Ångstroms) | | | |
| --- | --- | --- | --- | --- | --- |
| Center number | Atomic number | Atomic Type | X | Y | Z |
| 0 | 6 | 0 | 11.076348 | -3.365935 | 3.181295 |
| 1 | 6 | 0 | 13.233338 | -4.183186 | -0.967587 |
| 2 | 6 | 0 | 6.34727 | -3.369066 | -0.800286 |
| 3 | 6 | 0 | 8.547779 | -4.580091 | -0.583641 |
| 4 | 6 | 0 | 10.866751 | -3.225485 | 0.319849 |
| 5 | 8 | 0 | 10.730587 | -0.520134 | -0.378608 |
| 6 | 6 | 0 | 6.219307 | -0.682254 | -0.344865 |
| 7 | 6 | 0 | 8.48747 | 0.681692 | -0.19396 |
| 8 | 6 | 0 | 8.560163 | 3.327533 | 0.021435 |
| 9 | 7 | 0 | 1.56691 | -0.145299 | -0.327744 |
| 10 | 6 | 0 | 3.989054 | 0.725779 | -0.203809 |
| 11 | 6 | 0 | 4.042591 | 3.371958 | 0.0925 |
| 12 | 6 | 0 | 1.492733 | 4.220102 | 0.455191 |
| 13 | 6 | 0 | -1.980901 | 1.366003 | 2.399729 |
| 14 | 6 | 0 | -0.223547 | 1.905387 | 0.079548 |
| 15 | 6 | 0 | -2.103512 | 2.149828 | -2.244052 |
| 16 | 6 | 0 | -6.738788 | 2.026606 | 1.782857 |
| 17 | 6 | 0 | -4.429516 | 0.305578 | 1.28466 |
| 18 | 6 | 0 | -3.885064 | -0.02059 | -1.55636 |
| 19 | 6 | 0 | -6.333803 | -0.608401 | -2.950566 |
| 20 | 6 | 0 | -12.7171 | -0.669001 | 0.06894 |
| 21 | 6 | 0 | -11.374966 | 1.775029 | 0.781902 |
| 22 | 7 | 0 | -8.716146 | 1.084444 | 0.551641 |
| 23 | 6 | 0 | -8.325694 | -1.248633 | -0.928809 |
| 24 | 6 | 0 | -10.956857 | -1.887097 | -1.867354 |
| 25 | 8 | 0 | -6.758853 | 3.897496 | 3.181838 |
| 26 | 1 | 0 | -2.734717 | -1.727529 | -1.638818 |
| 27 | 6 | 0 | -7.202867 | -3.197818 | 0.940316 |
| 28 | 7 | 0 | -5.238767 | -2.172336 | 2.1828 |
| 29 | 8 | 0 | -8.007899 | -5.362927 | 1.254955 |
| 30 | 6 | 0 | 6.337405 | 4.669053 | 0.188404 |
| 31 | 8 | 0 | 0.728928 | 6.351637 | 1.01095 |
| 32 | 6 | 0 | -0.799071 | 1.686835 | -4.759482 |
| 33 | 6 | 0 | -3.419418 | 4.70766 | -2.342324 |
| 34 | 8 | 0 | -0.982246 | -0.34421 | 4.172893 |
| 35 | 6 | 0 | 0.828664 | 0.751246 | 5.841681 |
| 36 | 1 | 0 | 11.229583 | -5.329066 | 3.777902 |
| 37 | 1 | 0 | 12.743945 | -2.352863 | 3.83847 |
| 38 | 1 | 0 | 9.410946 | -2.557439 | 4.078767 |
| 39 | 1 | 0 | 14.874258 | -3.095778 | -0.371673 |
| 40 | 1 | 0 | 13.549156 | -6.152219 | -0.468114 |
| 41 | 1 | 0 | 13.061091 | -4.056218 | -3.013152 |
| 42 | 1 | 0 | 4.658789 | -4.360919 | -1.389756 |
| 43 | 1 | 0 | 8.721968 | -6.578459 | -0.975015 |
| 44 | 1 | 0 | 10.376211 | 4.256987 | 0.098004 |
| 45 | 1 | 0 | 1.158547 | -1.874451 | 0.348259 |
| 46 | 1 | 0 | -2.401542 | 3.167999 | 3.310683 |
| 47 | 1 | 0 | -7.055667 | 0.992242 | -4.020076 |
| 48 | 1 | 0 | -6.101194 | -2.180844 | -4.251041 |
| 49 | 1 | 0 | -14.600412 | -0.327772 | -0.674195 |
| 50 | 1 | 0 | -12.8897 | -1.875543 | 1.728056 |
| 51 | 1 | 0 | -11.775253 | 2.423795 | 2.68896 |
| 52 | 1 | 0 | -11.816046 | 3.30065 | -0.531755 |
| 53 | 1 | 0 | -11.219959 | -3.914937 | -2.037945 |
| 54 | 1 | 0 | -11.234095 | -1.046526 | -3.725514 |
| 55 | 1 | 0 | -4.202326 | -3.147941 | 3.451005 |
| 56 | 1 | 0 | 6.362926 | 6.699373 | 0.424772 |
| 57 | 1 | 0 | -2.157576 | 1.896796 | -6.293061 |
| 58 | 1 | 0 | 0.708838 | 3.050446 | -5.083164 |
| 59 | 1 | 0 | 0.005716 | -0.197715 | -4.878856 |
| 60 | 1 | 0 | -4.962335 | 4.641863 | -3.702107 |
| 61 | 1 | 0 | -4.193566 | 5.321555 | -0.546022 |
| 62 | 1 | 0 | -2.122768 | 6.169875 | -2.972078 |
| 63 | 1 | 0 | 0.032916 | 2.386478 | 6.81592 |
| 64 | 1 | 0 | 1.313667 | -0.692643 | 7.216653 |
| 65 | 1 | 0 | 2.540782 | 1.324774 | 4.848761 |

| **3h** | | Standard Orientation (Ångstroms) | | | |
| --- | --- | --- | --- | --- | --- |
| Center number | Atomic number | Atomic Type | X | Y | Z |
| 0 | 6 | 0 | 10.9514 | -3.66027 | 3.157388 |
| 1 | 6 | 0 | 13.079563 | -4.441685 | -1.013733 |
| 2 | 6 | 0 | 6.210138 | -3.502702 | -0.802967 |
| 3 | 6 | 0 | 8.388687 | -4.758321 | -0.620156 |
| 4 | 6 | 0 | 10.735704 | -3.464345 | 0.299889 |
| 5 | 8 | 0 | 10.647564 | -0.744493 | -0.349463 |
| 6 | 6 | 0 | 6.134268 | -0.823066 | -0.295766 |
| 7 | 6 | 0 | 8.42837 | 0.495286 | -0.131343 |
| 8 | 6 | 0 | 8.551878 | 3.134842 | 0.131923 |
| 9 | 7 | 0 | 1.492627 | -0.200303 | -0.246079 |
| 10 | 6 | 0 | 3.931731 | 0.623729 | -0.117947 |
| 11 | 6 | 0 | 4.03627 | 3.26292 | 0.224594 |
| 12 | 6 | 0 | 1.504556 | 4.152289 | 0.612667 |
| 13 | 6 | 0 | -2.021485 | 1.334335 | 2.513909 |
| 14 | 6 | 0 | -0.256577 | 1.878425 | 0.199215 |
| 15 | 6 | 0 | -2.132701 | 2.197686 | -2.118726 |
| 16 | 6 | 0 | -6.759025 | 2.132555 | 1.907105 |
| 17 | 6 | 0 | -4.497207 | 0.356979 | 1.384917 |
| 18 | 6 | 0 | -3.961274 | 0.057561 | -1.460575 |
| 19 | 6 | 0 | -6.423833 | -0.452783 | -2.861468 |
| 20 | 6 | 0 | -12.806192 | -0.378939 | 0.16105 |
| 21 | 6 | 0 | -11.399833 | 2.020738 | 0.900516 |
| 22 | 7 | 0 | -8.760442 | 1.262026 | 0.661694 |
| 23 | 6 | 0 | -8.43315 | -1.062731 | -0.847913 |
| 24 | 6 | 0 | -11.080708 | -1.619023 | -1.79229 |
| 25 | 8 | 0 | -6.729535 | 3.982432 | 3.333306 |
| 26 | 1 | 0 | -2.849708 | -1.673599 | -1.566941 |
| 27 | 6 | 0 | -7.365082 | -3.06448 | 0.996597 |
| 28 | 7 | 0 | -5.372419 | -2.110091 | 2.249345 |
| 29 | 8 | 0 | -8.230799 | -5.209564 | 1.286066 |
| 30 | 6 | 0 | 6.35563 | 4.514579 | 0.333421 |
| 31 | 8 | 0 | 0.783215 | 6.28686 | 1.212117 |
| 32 | 6 | 0 | -0.839371 | 1.743644 | -4.641677 |
| 33 | 6 | 0 | -3.388359 | 4.786984 | -2.179296 |
| 34 | 8 | 0 | -1.058665 | -0.437564 | 4.246398 |
| 35 | 6 | 0 | 0.779641 | 0.580444 | 5.934154 |
| 36 | 1 | 0 | 12.63783 | -2.687761 | 3.827498 |
| 37 | 1 | 0 | 9.302612 | -2.840176 | 4.074693 |
| 38 | 1 | 0 | 11.072645 | -5.63616 | 3.718175 |
| 39 | 1 | 0 | 13.359787 | -6.425559 | -0.553159 |
| 40 | 1 | 0 | 12.903299 | -4.272639 | -3.055932 |
| 41 | 1 | 0 | 14.742984 | -3.397312 | -0.403423 |
| 42 | 1 | 0 | 4.501103 | -4.450991 | -1.404971 |
| 43 | 1 | 0 | 8.523933 | -6.75137 | -1.05188 |
| 44 | 1 | 0 | 10.385362 | 4.028601 | 0.216736 |
| 45 | 1 | 0 | 1.055257 | -1.928394 | 0.415039 |
| 46 | 1 | 0 | -2.394665 | 3.125166 | 3.466212 |
| 47 | 1 | 0 | -7.103467 | 1.176538 | -3.915222 |
| 48 | 1 | 0 | -6.232239 | -2.01716 | -4.178363 |
| 49 | 1 | 0 | -14.681576 | 0.019242 | -0.573763 |
| 50 | 1 | 0 | -13.005586 | -1.599622 | 1.806714 |
| 51 | 1 | 0 | -11.782493 | 2.658461 | 2.814899 |
| 52 | 1 | 0 | -11.799952 | 3.57198 | -0.396156 |
| 53 | 1 | 0 | -11.397669 | -3.636955 | -1.987519 |
| 54 | 1 | 0 | -11.337178 | -0.750109 | -3.640336 |
| 55 | 1 | 0 | -4.364848 | -3.128565 | 3.507273 |
| 56 | 1 | 0 | 6.420846 | 6.539482 | 0.606025 |
| 57 | 1 | 0 | -2.190257 | 2.017904 | -6.171792 |
| 58 | 1 | 0 | 0.704009 | 3.072816 | -4.940004 |
| 59 | 1 | 0 | -0.084272 | -0.158958 | -4.795166 |
| 60 | 1 | 0 | -2.058795 | 6.225065 | -2.796227 |
| 61 | 1 | 0 | -4.9387 | 4.776709 | -3.532361 |
| 62 | 1 | 0 | -4.139415 | 5.397369 | -0.37226 |
| 63 | 1 | 0 | 2.50017 | 1.142254 | 4.94903 |
| 64 | 1 | 0 | 0.020545 | 2.207779 | 6.949783 |
| 65 | 1 | 0 | 1.239216 | -0.90578 | 7.272392 |

| **3i** | | Standard Orientation (Ångstroms) | | | |
| --- | --- | --- | --- | --- | --- |
| Center number | Atomic number | Atomic Type | X | Y | Z |
| 0 | 6 | 0 | 13.130568 | -4.37973 | 1.547332 |
| 1 | 6 | 0 | 11.062454 | -3.892263 | -2.699782 |
| 2 | 6 | 0 | 6.262484 | -3.52398 | 1.202124 |
| 3 | 6 | 0 | 8.45124 | -4.769719 | 1.09696 |
| 4 | 6 | 0 | 10.800125 | -3.512618 | 0.13531 |
| 5 | 8 | 0 | 10.67887 | -0.758337 | 0.601673 |
| 6 | 6 | 0 | 6.163583 | -0.875491 | 0.54841 |
| 7 | 6 | 0 | 8.449322 | 0.445295 | 0.300773 |
| 8 | 6 | 0 | 8.555558 | 3.062181 | -0.136082 |
| 9 | 7 | 0 | 1.512978 | -0.30321 | 0.365901 |
| 10 | 6 | 0 | 3.950864 | 0.540321 | 0.269386 |
| 11 | 6 | 0 | 4.039747 | 3.163429 | -0.182867 |
| 12 | 6 | 0 | 1.497537 | 4.10847 | -0.118515 |
| 13 | 6 | 0 | -2.111544 | 1.944524 | 2.407401 |
| 14 | 6 | 0 | -0.246914 | 1.799031 | 0.114095 |
| 15 | 6 | 0 | -2.021427 | 1.395016 | -2.270584 |
| 16 | 6 | 0 | -6.816229 | 2.467985 | 1.390149 |
| 17 | 6 | 0 | -4.536763 | 0.644353 | 1.515935 |
| 18 | 6 | 0 | -3.872363 | -0.481738 | -1.083606 |
| 19 | 6 | 0 | -6.267459 | -1.418714 | -2.374646 |
| 20 | 6 | 0 | -12.784433 | -0.518624 | 0.202703 |
| 21 | 6 | 0 | -11.409309 | 2.008509 | 0.274136 |
| 22 | 7 | 0 | -8.760587 | 1.24695 | 0.367268 |
| 23 | 6 | 0 | -8.371374 | -1.41767 | -0.365033 |
| 24 | 6 | 0 | -10.973738 | -2.254144 | -1.225129 |
| 25 | 8 | 0 | -6.850398 | 4.653279 | 2.214042 |
| 26 | 1 | 0 | -2.753525 | -2.149835 | -0.6256 |
| 27 | 6 | 0 | -7.391709 | -2.773274 | 2.030344 |
| 28 | 7 | 0 | -5.462759 | -1.460857 | 3.039645 |
| 29 | 8 | 0 | -8.259123 | -4.758228 | 2.891209 |
| 30 | 6 | 0 | 6.350319 | 4.422114 | -0.365496 |
| 31 | 8 | 0 | 0.759995 | 6.318615 | -0.190599 |
| 32 | 6 | 0 | -0.628119 | 0.231532 | -4.494787 |
| 33 | 6 | 0 | -3.281006 | 3.832728 | -3.14653 |
| 34 | 8 | 0 | -1.22769 | 0.77662 | 4.625603 |
| 35 | 6 | 0 | 0.576746 | 2.248544 | 5.983246 |
| 36 | 1 | 0 | 12.919502 | -4.080821 | 3.571231 |
| 37 | 1 | 0 | 13.437712 | -6.386178 | 1.220864 |
| 38 | 1 | 0 | 14.794198 | -3.360839 | 0.895725 |
| 39 | 1 | 0 | 11.196769 | -5.899038 | -3.133646 |
| 40 | 1 | 0 | 9.426065 | -3.136373 | -3.691074 |
| 41 | 1 | 0 | 12.756545 | -2.958563 | -3.404941 |
| 42 | 1 | 0 | 4.559555 | -4.470745 | 1.823038 |
| 43 | 1 | 0 | 8.590358 | -6.743241 | 1.610093 |
| 44 | 1 | 0 | 10.384002 | 3.957556 | -0.288477 |
| 45 | 1 | 0 | 1.060678 | -1.727121 | 1.541776 |
| 46 | 1 | 0 | -2.524705 | 3.932413 | 2.76801 |
| 47 | 1 | 0 | -6.892447 | -0.194857 | -3.902978 |
| 48 | 1 | 0 | -6.016349 | -3.305621 | -3.145926 |
| 49 | 1 | 0 | -14.621815 | -0.371577 | -0.701954 |
| 50 | 1 | 0 | -13.0692 | -1.211279 | 2.120687 |
| 51 | 1 | 0 | -11.868994 | 3.166234 | 1.907012 |
| 52 | 1 | 0 | -11.758284 | 3.116838 | -1.427922 |
| 53 | 1 | 0 | -11.285849 | -4.245685 | -0.843969 |
| 54 | 1 | 0 | -11.137893 | -1.956292 | -3.254735 |
| 55 | 1 | 0 | -4.476441 | -2.094984 | 4.543205 |
| 56 | 1 | 0 | 6.399863 | 6.440183 | -0.68769 |
| 57 | 1 | 0 | -1.929118 | -0.009775 | -6.072943 |
| 58 | 1 | 0 | 0.900564 | 1.448006 | -5.14523 |
| 59 | 1 | 0 | 0.163123 | -1.606627 | -4.037992 |
| 60 | 1 | 0 | -4.81249 | 3.393153 | -4.448382 |
| 61 | 1 | 0 | -4.060782 | 4.966436 | -1.626885 |
| 62 | 1 | 0 | -1.944362 | 5.018382 | -4.159532 |
| 63 | 1 | 0 | -0.167518 | 4.128942 | 6.392002 |
| 64 | 1 | 0 | 0.942354 | 1.259888 | 7.743768 |
| 65 | 1 | 0 | 2.348189 | 2.436566 | 4.947098 |

**The rDNA-ITS sequence of the fungal strain *Aspergillus sclerotiorum* GDST-2013-0501**

GTTTTGAGGTGAGGGGTCCTCGGGGCCCACCTCCCACCCGTGTATACCGTACCTTGTTGCTTCGGCGGGCCCGCCGCGCAAGCGGCCGCCGGGGGGGGCGTCAAACCCCCCTCCCTAGGCGAGCGCCCGCCGGAGACACCAACGTGAACACTGTCTGAAGTTTTGTTGTCTGAGTTCGATTGTATCGCAATCAGTTAAAACTTTCAACAATGGATCTCTTGGTTCCGGCATCGATGAAGAACGCAGCGAAATGCGATAATTAATGTGAATTGCAGAATTCAGTGAATCATCGAGTCTTTGAACGCACATTGCACCCCCTGGTATTCCGGGGGGTATGCCTGTCCGAGCGTCATTGCTGCCCTCAAGCACGGCTTGTGTGTTGGGTCGTCGTCCCCCCGGGGACGGGCCCGAAAGGCAGCGGCGGCACCGCGTCCGGTCCTCGAGCGTATGGGGCTTTGTCACCCGCTCTTGTAGGCCCGGCCGGCGCTGGCCGACGCTGAAAAGCAACCAACTATTTCTCCAGGTTGACCTCGGATCAGGTAGGGATACCCGCTGAACTTAAGCATATCAATAAGGCGGAGGAAAAAAAA
